# Multi-environment GWAS uncovers markers associated to biotic stress response and genotype-by-environment interactions in stone fruit trees

**DOI:** 10.1093/hr/uhaf088

**Published:** 2025-04-22

**Authors:** Marie Serrie, Vincent Segura, Alain Blanc, Laurent Brun, Naïma Dlalah, Frédéric Gilles, Laure Heurtevin, Mathilde Le Pans, Véronique Signoret, Sabrina Viret, Jean-Marc Audergon, Bénédicte Quilot, Morgane Roth

**Affiliations:** INRAE, UR GAFL, 84140 Avignon, France; AGAP Institut, CIRAD, INRAE, Institut Agro, Université Montpellier, 34000 Montpellier, France; Geno-Vigne®, IFV, INRAE, Institut Agro, 34000 Montpellier, France; INRAE, UE A2M, 84140 Avignon, France; INRAE, UERI Gotheron, 26320 Saint-Marcel-Lès-Valence, France; INRAE, UR GAFL, 84140 Avignon, France; INRAE, UR GAFL, 84140 Avignon, France; INRAE, UR GAFL, 84140 Avignon, France; INRAE, UERI Gotheron, 26320 Saint-Marcel-Lès-Valence, France; INRAE, UR GAFL, 84140 Avignon, France; INRAE, UR GAFL, 84140 Avignon, France; INRAE, UR GAFL, 84140 Avignon, France; INRAE, UR GAFL, 84140 Avignon, France; INRAE, UR GAFL, 84140 Avignon, France

## Abstract

While breeding for improved immunity is essential to achieve sustainable fruit production, it also requires to account for genotype-by-environment interactions (G × E), which still represent a major challenge. To tackle this issue, we conducted a comprehensive study to identify genetic markers with main and environment-specific effects on pest and disease response in peach (*Prunus persica*) and apricot (*Prunus armeniaca*). Leveraging multienvironment trials (MET), we assessed the genetic architecture of resistance and tolerance to seven major pests and diseases through visual scoring of symptoms in naturally infected core collections, repeated within and between years and sites. We applied a series of genome-wide association models (GWAS) to both maximum of symptom severity and kinetic disease progression. These analyses lead to the identification of environment-shared quantitative trait loci (QTLs), environment-specific QTLs, and interactive QTLs with antagonist or differential effects across environments. We mapped 60 high-confidence QTLs encompassing a total of 87 candidate genes involved in both basal and host-specific responses, mostly consisting of the Leucine-Rich Repeat Containing Receptors (LRR-CRs) gene family. The most promising disease resistance candidate genes were found for peach leaf curl on LG4 and for apricot and peach rust on LG2 and LG4. These findings underscore the critical role of G × E in shaping the phenotypic response to biotic pressure, especially for blossom blight. Last, models including dominance effects revealed 123 specific QTLs, emphasizing the significance of non-additive genetic effects, therefore warranting further investigation. These insights will support the development of marker-assisted selection to improve the immunity of *Prunus* varieties in diverse environmental conditions.

## Introduction

Biotic stresses represent a major threat to agricultural production as a vast array of pathogens and pests, including a large range of viruses, bacteria, fungi, phytoplasma, nematodes, and insects, can negatively impact plant health [1, 2]. In stone fruit, reducing the impact of these biotic stresses in a sustainable and environment-friendly way is both urgent and tedious given their long juvenile phase and rotation cycles. One of the most promising approaches to overcoming these challenges is the development of durable resistance and tolerance through breeding, which requires an in-depth understanding of the genetic components underlying these traits [3, 4]. Notably, certain landraces and wild relatives of cultivated stone fruits harbor valuable resistance and tolerance alleles. For instance, *Prunus davidiana*, a close relative of peach (*Prunus persica*), carries genes conferring resistance to green peach aphids, powdery mildew, and Plum pox virus (PPV) [5–7]. Therefore, harnessing the natural genetic diversity of stone fruits holds great potential for improving disease resistance [8]. Genome-Wide Association Studies (GWAS) have become a powerful tool for dissecting the genetic basis of complex traits, including quantitative disease resistance and tolerance [9, 10]. By leveraging ancestral linkage disequilibrium within diverse germplasm collections, GWAS enables the identification of quantitative trait loci (QTLs) associated with traits of interest. While GWAS has been widely applied in various crop species [11], its use in stone fruit species remains limited, particularly for biotic stress-related traits [7, 12–16]. The identification of QTLs through GWAS has the potential to accelerate breeding via marker-assisted selection (MAS) [17, 18]. Yet, detecting QTLs linked to disease resistance or tolerance is challenging due to the strong influence of environmental effects and genotype by environment (G × E) interactions [19–21]. These interactions often reduce trait heritability, complicating breeding efforts by altering the expression and effect size of QTLs across different environmental conditions.

To address these challenges, Multienvironment Trials (MET) are commonly employed to evaluate phenotypic responses under diverse environmental conditions [22, 23]. Single-environment GWAS can be used as a first step to identify QTLs separately in different environments while multienvironment GWAS models allows for the detection of both main-effect QTLs and QTLs with environment-dependent effects (QTL × Environments interactions) [24, 25]. The Multitrait Mixed Model (MTMM), *e.g.* [26] has successfully been used, firstly in *Arabidopsis thaliana* for the study of flowering time QTLs under different temperatures, and later for plant architecture in barley and in production traits in common bean under contrasted abiotic stress conditions [27–29]. Another complementary approach, meta-analysis GWAS, can enhance the detection of minor-effect QTLs that might go undetected in individual studies, as well as confirm previously identify marker–trait associations [30–33]. Importantly, meta-GWAS is particularly well suited to plant breeding applications, as it allows for the integration of data from unbalanced phenotypic datasets (*i.e.* asymmetrical proportions between class of observation) without requiring replication across all environments [34].

Taking advantage of the recent availability of high-quality reference genomes [35–38] and the development of genotyping tools such as single nucleotide polymorphism (SNP) arrays for peach [39], GWAS has gradually become more widespread in *Prunus* species for determining the genetic basis of variation in complex traits with little attention to G × E variance [40–45]. Overall, it turns out that the genetic determinants of resistance and tolerance to major biotic stresses in the face of multiple environments have been little investigated in apricot and peach, and therefore warrant detailed study [13].

Furthermore, phenotypic variation is not solely driven by additive genetic effects; non-additive effects such as dominance and epistasis also play a crucial role and are vastly exploited in heterosis breeding [46, 47]. These non-additive effects are only partially captured in traditional GWAS approaches, yet they can significantly improve predictions of total genetic variance and facilitate the identification of complementary QTLs [48, 49]. Although few studies have investigated non-additive variance in fruit trees, mostly in the frame of genomic prediction [50–52], recent research on almond suggests that non-additive effects may contribute substantially to trait variation [53]. Thus, incorporating non-additive models in GWAS can therefore enhance our ability to uncover the full spectrum of genetic factors underlying complex traits [54, 55].

In this context, our study aims to elucidate the genetic architecture of pest and disease resistance and tolerance in the two closely related species peach (*P. persica* L.) and apricot (*Prunus armeniaca* L.) in multiple environments using GWAS. These species present ideal models for such research due to their i) diploid genomes with small sizes (230 and 295 Mb, respectively) [56], ii) extensive yet underutilized phenotypic diversity available in germplasm collections [57, 58], and iii) high levels of syntenic, thus facilitating comparative analysis between them [59]. To achieve this, we built a comprehensive phenotypic database comprising 206 peach and 150 apricot accessions, monitored under low phytosanitary protection in three and two sites in the South-East of France, respectively. We recorded and analyzed multiple stress factors, including leaf curl, leafhopper, powdery mildew, rust, and shot hole for peach as well as blossom blight and rust for apricot. A key strength of our study is the repeated monitoring of pest and disease symptoms within and between years and sites, allowing us to capture both the maximum of contamination and kinetic disease progression and to decipher G × E interactions.

To dissect the genetic basis of disease resistance, we implemented a multifaceted GWAS approach, aimed at i) investigating the main additive and non-additive genetic effects across environments, ii) comparing environment-specific effects with single-environment GWAS, and iii) harnessing G × E interactions effects using multienvironment GWAS and meta-analysis GWAS. Our extensive experimental network and high-frequency phenotyping under natural biotic pressure enabled us to discover QTLs controlling resistance or tolerance to seven pests and diseases, five of which have never been previously studied at the genetic level. The consistent experimental design and unified analytical approach across peach and apricot allowed for robust cross-species comparisons.

Our findings provide novel insights into the genetic basis of disease resistance in *Prunus* species and lays a foundation for more targeted breeding strategies to enhance the immunity of fruit trees across environments.

## Results

### Phenotypic response of peach and apricot accessions to natural infection in multiple orchards

The first step of our analyses aimed at evaluating the biotic performance of the two core collections and to assess the effects of environment, genotype, and G × E interactions on biotic stress responses.

#### A large variability in the phenotypic response to biotic stress in the two core collections

We scored trees at different time points for each biotic stress throughout the season to fully capture the dynamic expression of the phenotypic diversity under low phytosanitary protection. This allowed us to produce two types of variables for each tree and each biotic stress, namely the i) maximum of susceptibility (Max) and ii) the Area under the Disease Progress Curve (AUDPC).

**Figure 1:**
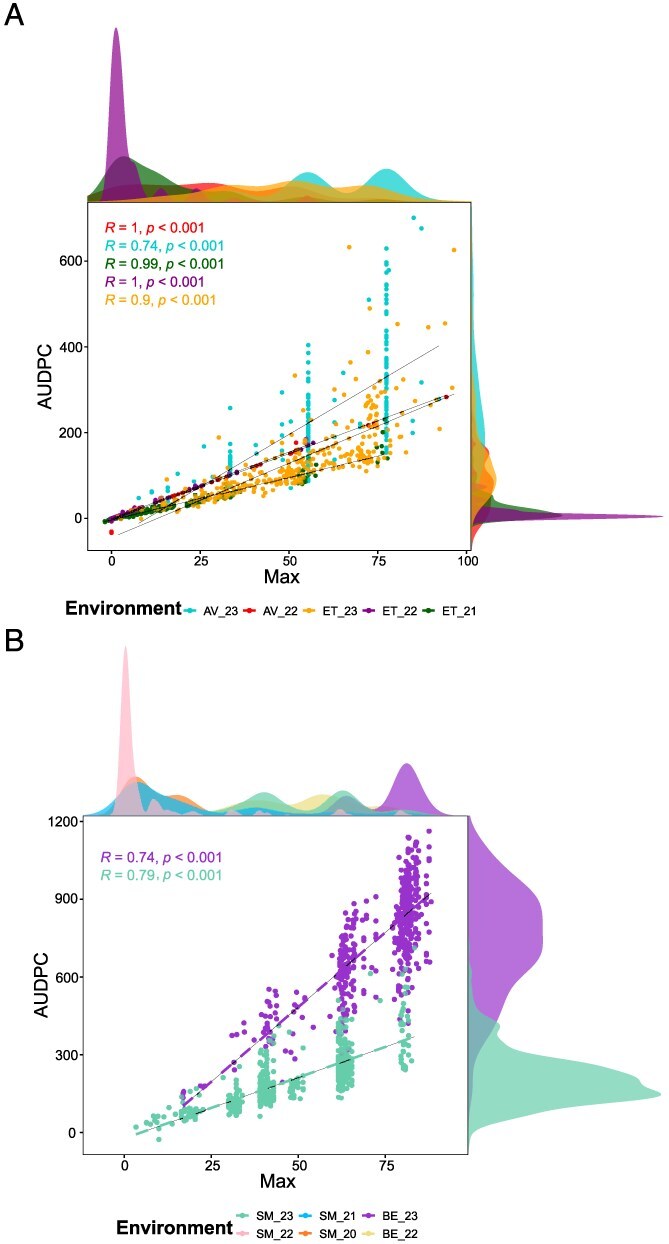
Phenotypic distribution and correlation between Max and AUDPC for (A) peach rust and (B) apricot rust. Distributions of Max are shown at the top of each graph, while the distributions of AUDPC are shown on the right. AV: Avignon, ET: Étoile-sur-Rhône, TO: Torreilles, SM: Saint-Marcel-lès-Valence, and BE: Bellegarde.

After spatial correction, we cleaned our dataset in order to retain only environments with sufficient expression of phenotypic variability for each trait, leading to the removal of 19.6% of the data (Supplementary Method 2 and Supplementary Fig. S1). In the remaining dataset the average standard deviation was of 31.1 and 295.4, respectively, for Max and AUDPC. We found the highest diversity of response for apricot rust leaf fall for both Max and AUDPC (with standard deviation of 34.1 and 321.9, respectively). For an equivalent value of Max, trees presented markedly different temporal trajectories for this disease, as illustrated by diverging onset dates and spread of symptoms, with marked environment-specific effects (Fig. 1 and Supplementary Fig. S3). While correlations between Max and AUDPC remained high for all biotic stresses (from 0.676 to 0.997) (Fig. 1 and Supplementary Fig. S2), AUDPC thus provided complementary information on the response of accessions to biotic stresses.

To characterize the stability of symptom expression across the environment, we calculated the correlations between environment-specific best linear unbiased predictors (BLUPs) for each pest or disease. The genetic correlations between environments ranged from moderate to strong, with average Pearson’s correlation coefficients of 0.68 and 0.73 for Max and AUDPC, respectively (Supplementary Table S1 and Supplementary Fig. S4). We found the lowest genetic correlation between environments for blossom blight (*r* = 0.41 in average for Max) and the highest correlations for leaf curl (*r* = 0.87 and *r* = 0.91 in average for Max and AUDPC, respectively) (Supplementary Table S1). Overall, symptoms were more correlated within a given location than within a given year for both Max and AUDPC (Supplementary Table S1).

#### Biotic stress responses are modulated by G and G × E effects

**Figure 2:**
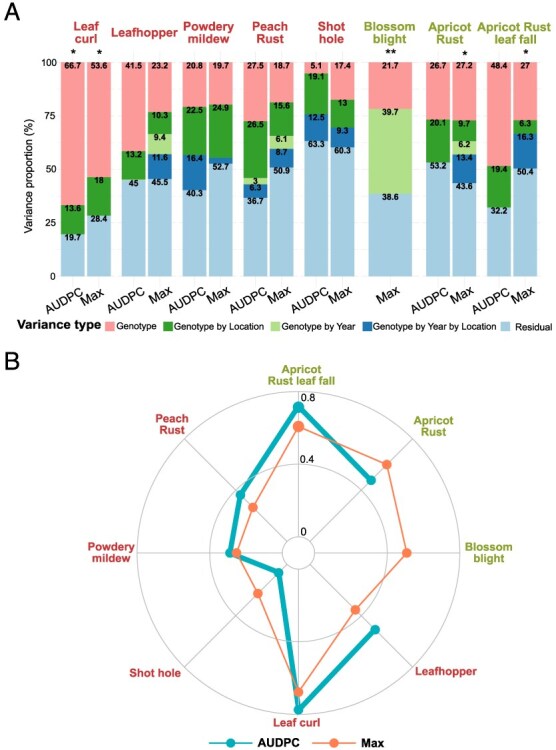
(A) Stacked bar plots with the variance of the random effects of genotype, genotype by location, genotype by year and genotype by year by location interactions, and residuals; calculated from the model presenting in Eq. (2) of Supplementary Method 3 and (B) broad sense heritability calculated from Eq. (3) Supplementary Method 3. *Biotic stress observed only one year. **Biotic stress observed only in one location

We computed a linear mixed model for each pest and disease to quantify the variance associated to genotype, genotype by location, genotype by year, and genotype by year by location interactions (Fig. 2A and Supplementary Method 3). The variance attributed to the genotype ranges from 5.1% to 66.7% of the total variance. For powdery mildew and shot hole, the genotypic variance was lower than the variance attributed to the G × L interactions (for both Max and AUDPC variables), and for blossom blight lower than the variance attributed to the G × Y interactions (39.7% of total variance). The contribution on the G × L effect on the trait variance was moderate to high (6.3%–26.5%) and was higher than the variance explained by G × Y for all biotic stresses, as already expected from previous correlation analysis (Fig. 2A). Finally, G × Y × L variance could not be assessed for all traits and remained moderate (2.7%–16.4%). Generally speaking, all responses to biotic stresses were found to be influenced by G × E interactions, with the response to leaf curl being the most stable across environments.

Overall, broad-sense heritability estimates across environments on spatially corrected Max and AUDPC variables ranged from 0.07 to 0.78 for the eight pests and diseases studied (Fig. 2B). Heritability was generally higher when considering AUDPC rather than Max.

When dissecting between additive and non-additive variance, we found that the dominance variance represented up to 16.4% of the total variance, depending on the trait, the highest proportions being found for apricot rust (Supplementary Fig. S5). Except for powdery mildew, the dominance variance was higher when considering Max as compared to AUDPC. A comparison of models for these across-environment variance decompositions using the BIC criterion suggested that the Additive-Dominant model was the best model choice for five traits (leafhopper Max and AUDPC, powdery mildew Max and AUDPC, and apricot rust Max).

### Genome-wide association analyses for biotic stress responses in the two core collections

We exploited genetic and G × E variance by applying a series of complementary GWAS models whereby phenotypes were considered i) across environments to harness global genetic effects (using G-BLUPs), ii) within-environment (environment-specific BLUPs), and iii) in multiple environments with models accounting for G × E. For each analysis, significant SNPs were grouped in QTLs by considering linkage blocks using the chromosome-specific linkage disequilibrium (LD) decay values (ranging between 508 and 1620 kb and between 131 and 473 bp, respectively, in the peach and the apricot core collection, depending on the chromosome) (Supplementary Fig. S6).

#### Complementarity between additive and non-additive QTLs identified across environments

To exploit both additive and non-additive main genetic effects, four different models were tested on G-BLUPs, representing the different modes of action of genes: additive, dominance, recessive, and overdominance (the last three have been grouped as ‘non-additive’; *see Material and Methods section*).

**Figure 3:**
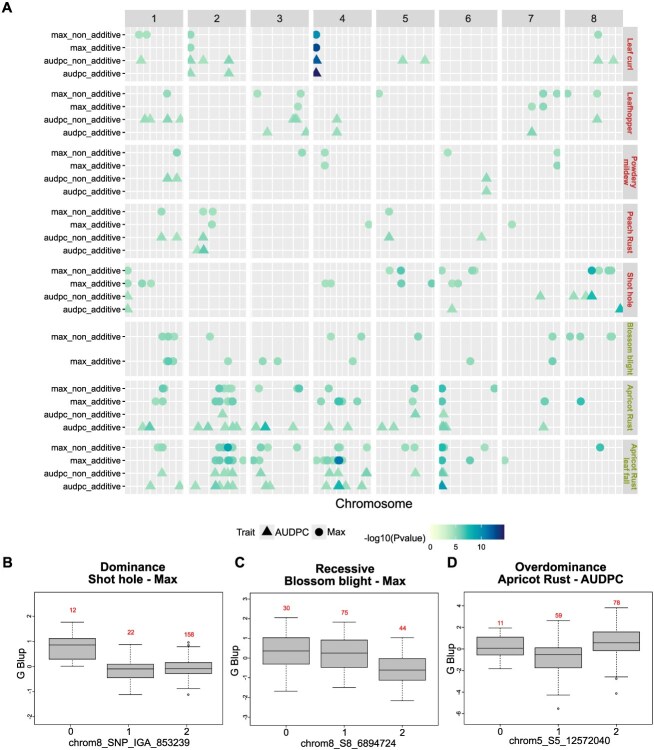
GWAS results on main additive and non-additive genetic effect with (A) QTL summary showing the physical position of GWAS-significant SNPs. Each horizontal line contains QTLs of one biotic stress, organized by additive or non-additive analysis and either for the maximum of damage score (called Max) or the AUDPC. The dot colors are proportional to the -log_10_(*P*-value) of the QTL top SNP. The *x*-axis indicates the physical positions on the peach or apricot genome. Boxplots illustrating the effect of a (B) dominant, (C) recessive, and (D) overdominant SNP with a significant association for, respectively, shot hole (Max), blossom blight (Max), and apricot rust (AUDPC).

When considering the union of QTLs obtained for Max and AUDPC (*i.e.* detected for one, the other, or both variables), we identified between 10 and 131 QTLs per trait (Fig. 3A). Within the total of 341 QTLs detected, 188 were additive, 153 were non-additive, and 30 were detected with both additive and non-additive models (Fig. 3A). Except for apricot rust and rust leaf fall, more QTLs were identified with non-additive as with additive models (between 1.2 and 3.7 times more QTLs depending on the trait). Among the non-additive models, the recessive model identified the most QTLs across all traits (between 1 and 28 with an average of 8.8) (Fig. 3C and Supplementary Fig. S7), while the overdominance model identified the least (between 0 and 19 across traits, with an average of 4.9) (Fig. 3D and Supplementary Fig. S7). However, it should be reminded that dominant and recessive models are only differentiated regarding the effects of the reference allele. Therefore, QTLs detected with both types of models can correspond to a recessive or dominant mode of action.

We identified 271 and 119 QTLs when considering Max and AUDPC traits, respectively, with a total overlap of 41 QTLs. Among the diseases studied, apricot rust had the least overlap between Max and AUDPC QTLs (<1% shared), while leaf curl showed the highest overlap (26% of QTLs) (see Fig. 3A).

Overall, the no-nadditive GWAS analysis allowed identifying complementary QTLs that were not detected by the additive GWAS analysis, with 96 and 45 QTLs being exclusive to non-additive models, respectively, for Max and AUDPC.

#### Identification of environment-specific QTLs with single-environment GWAS

**Figure 4:**
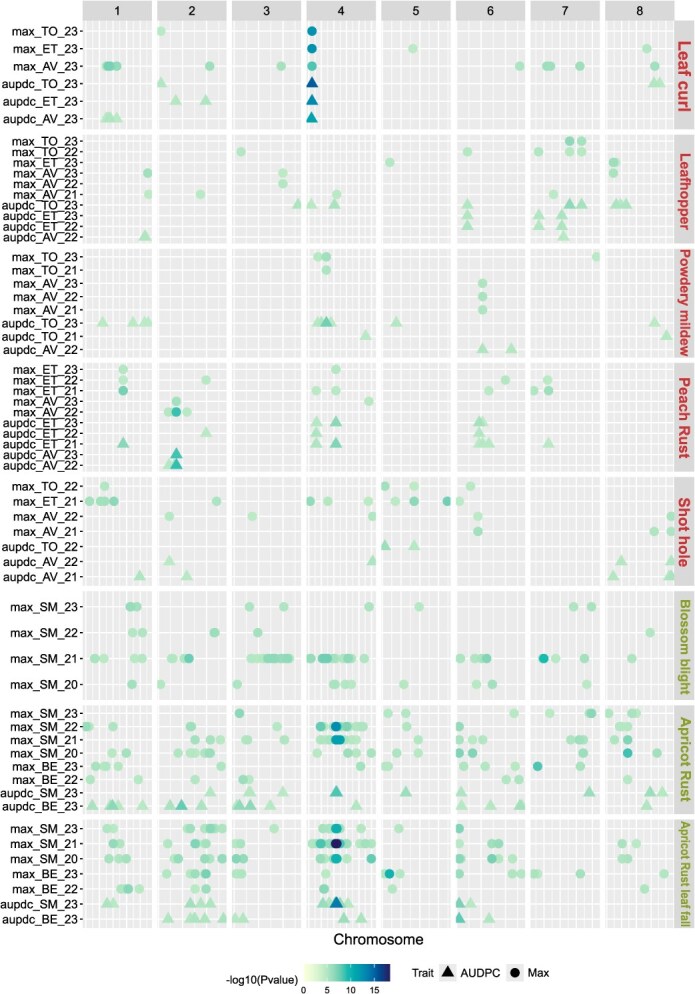
QTL summary showing the physical positions of GWAS-significant SNPs for environment-specific models. Each horizontal line contains QTLs of one biotic stress, organized by environment and either for the maximum of damage score (called Max) or the AUDPC (except for blossom blight, where AUDPC was not available). The colored dots are proportional to the -log_10_(*P*-value) of the QTL top SNP. The *x*-axis indicates the physical positions on the genome (peach or apricot depending on trait).

In order to study environment-specific effects, we implemented single-environment GWAS using single-Locus Mixed Models on environment-specific BLUPs. We identified between 33 and 568 significant SNPs depending on the trait, corresponding to a total of 801 QTLs (union of results on Max and AUDPC variables) (Fig. 4).

The identity and number of associations were greatly dependent on the target environment (62.5%–100% environment-specific QTLs depending on trait) (Fig. 4). Leaf curl was the only trait for which a QTL was identified in all investigated environments and for both AUDPC and Max variables. Apart from this QTL, located on chromosome 4, the other 25 QTLs associated with leaf curl were detected in only one environment. For apricot rust and rust leaf fall, some QTLs have also been identified in all the environments studied when considering AUDPC (representing respectively 5% and 3% of all the QTLs identified). With a total of six QTLs detected in two different environments and three QTLs detected in three different environments (total of 5 environments considered), peach rust was the trait with the highest proportion of stable QTLs. On the opposite, blossom blight was the disease with less stable QTLs as all of them were found to be environment-specific. For all traits, QTLs associated to Max appeared to be more stable than those found with AUDPC (22% and 0.09% of QTLs detected in more than one environment, respectively). Finally, among the QTLs identified, between 2 and 46 QTLs overlapped between Max and AUDPC depending on the biotic stress.

#### Modeling QTLs with G × E interaction effects using MTMM and Meta-GWAS

To statistically distinguish between stable QTLs across environments and those underlying G × E interactions, we performed i) a multienvironment analysis on environment-specific BLUPs using the fully parameterized MTMM [26] comprising three submodels (‘common model’, ‘gei model’, and ‘full model’) (Supplementary Method 6), and ii) a Meta-GWAS with the MetaGE model [33](Supplementary Method 7). MTMM disentangle common effect markers from interactive markers and MetaGE jointly analyzes single-environment GWAS results to identify QTLs with minor effects that might go undetected in single-environment analyses, as well as markers with significant effects across multiple environments.

**Figure 5:**
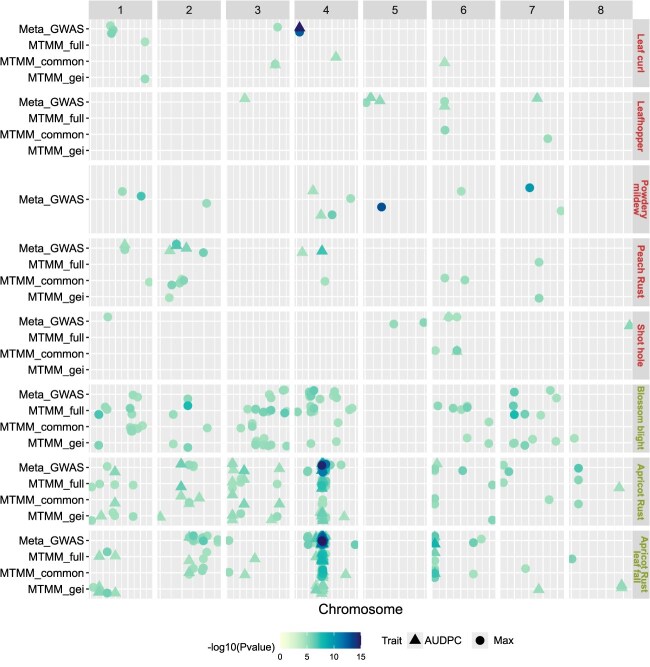
QTL summary showing the physical position of GWAS-significant SNPs for the two models dissecting the G × E interactions (MTMM and Meta-GWAS). Each horizontal line contains QTLs of one biotic stress, organized by GWAS model (Meta-GWAS and the three submodels of MTMM: full, common, and gei). The dot colors are proportional to the -log_10_(*P*-value) of the QTL top SNP and the dot shape represents the considered variable (Max or AUDPC). The *x*-axis indicates the physical positions on the peach or apricot genome.

MTMM GWAS and Meta-GWAS analyses led to the identification of respectively 209 and 226 QTLs significantly associated when considering all traits (union of Max and AUDPC), with 1–61 QTLs per trait identified with MTMM and 1–54 QTLs per trait identified with Meta-GWAS (Fig. 5). From those, 107 QTLs overlapped between MTMM and MetaGE approaches.

**Figure 6:**
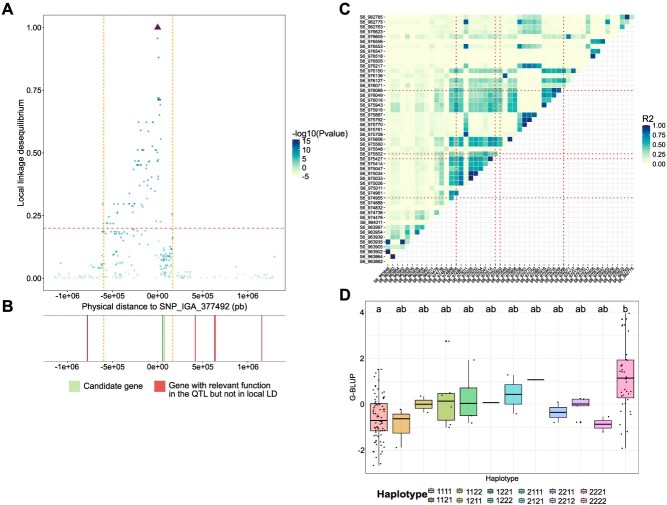
Fine characterization of two high-confidence QTLs. (A) Representation of the local LD between the top SNP of QTL ‘Leaf_curl_4’ and all other SNPs of the QTL. The *x*-axis represents the physical distance from the top SNP, while the *y*-axis represents the r^2^, corrected by the relatedness and structure, between SNPs and the top SNP. Each dot represents a SNP that is colored according the *P*-value of Meta-GWAS. The vertical orange dot lines represent the QTL interval readjusted using the local LD. (B) Genes with relevant functions located in the QTL, with candidate genes in green and genes located in the QTL but not in local LD with the top SNP in red. (B) Relevant genes located in the QTL, with candidate genes in green and relevant genes located in the QTL but not in local LD with the top SNP in red. (C) Heatmaps of pairwise LD estimates within the genomic window around candidate gene ‘PruarM.6G017900’. Top SNPs of the different high-confidence QTLs are shown with red dot line and (D) G-BLUPs distributions of apricot rust Max according to the haplotype constituted by the SNPs ‘S6_974955’, ‘S6_975427’, ‘S6_975502’, and ‘S6_976066’. Statistical differences between groups were computed by pairwise Wilcoxon tests.

Due to difficulties in computing genetic and environment variance covariance matrices needed in the MTMM model (Supplementary Method 6), MTMM analysis could not be performed on four variables (AUDPC of peach rust and leafhopper, and on both Max and AUDPC for powdery mildew). These difficulties probably owe to the amount of missing data for some environments in the datasets of those traits, which we could not alleviate. For the remaining, we identified 75 and 67 QTLs, respectively, for ‘common’ and ‘gei’ models (union of Max and AUDPC, Fig. 5). For most traits, more QTLs have been identified with the ‘common’ model than with the two other MTMM models (between 50% and 100% of QTLs detected). However, for blossom blight (Max), apricot rust (AUDPC and Max), and apricot rust leaf fall (Max), 53%–65% of QTLs were identified with the ‘gei’ model. For these ‘gei’, interactive QTLs, allelic effects of the top SNP ranged from negative to highly positive depending on environment.

When performing Meta-GWAS, we found a large amplitude of *z*-scores—a summary statistic of the marker’s *P*-value of the sign of the marker’s effects—for most SNPs. We observed only rare sign inversions, indicative of SNPs with the same qualitative effects (*i.e.* same direction), but quantitatively different effects across environments (Supplementary Fig. S8). A remarkable exception was found for blossom blight, with 61% of the significant SNPs presenting a *z*-score sign inversion between the four environments considered (Supplementary Fig. S8F). Interestingly, we detected 29 QTLs with Meta-GWAS that had not been detected with single-environment GWAS (Fig. 4 and Fig. 5).

### Identification of high-confidence QTLs and quantification of their contribution to G and G × E interactions

When considering all traits and models tested, 101 QTLs were detected for peach and 606 for apricot. To retain the most relevant QTLs, high-confidence QTLs were defined as those detected in at least 50% of the additive GWAS models tested. We retained up to 32 high-confidence QTLs depending on the trait (Supplementary Table S2). Blossom blight was the only disease for which no confidence QTLs could be identified. High-confidence QTLs overlapping between AUDPC and Max have been found for leaf curl (1 QTL located on chromosome 4), apricot rust (1 QTL located on chromosome 4), and apricot rust leaf fall (18 QTLs located on chromosomes 2, 4, and 6) (Supplementary Table S2).

For each trait, we calculated the proportions of G, G × L, and G × Y variance captured by the high-confidence QTLs (considered jointly when several confident QTLs were identified for a given pest or disease). The high-confidence QTLs captured between 9.6% and 64.6% of the additive genetic variance depending on the biotic stress (Supplementary Fig. S9). These QTLs also explained variable portions of G × L interactions (up to 54.4%) and to a lesser extent, of G × Y interactions (up to 29.3%) (Supplementary Fig. S9). Interestingly, in apricot rust high-confidence QTLs explained a higher proportion of the G × L variance than of the genetic variance (Max variable) (Supplementary Fig. S9).

### Local LD and annotation analyses within high-confidence QTLs toward identifying candidate genes

Based on the association results, we searched within high-confidence QTLs for candidate genes with predicted functions linked to host defense or response mechanisms. As a preliminary step, we readjusted peach QTL intervals to account for the strong LD observed in peach by considering the local LD between the top SNP of each high-confidence QTL and all SNPs belonging to this QTL. We considered that a SNP was independent from the top SNP when its *r*^2^ to top SNP was <0.2 (Fig. 6A and B for Leaf_curl_4 and Supplementary Fig. S10 for the other peach QTLs). This enabled us to narrow down the list of candidate genes for peach, with an average of 34.6% of genes with a relevant function located in the readjusted QTL intervals (Table 1 and Supplementary Table S3).

**Table 1:** List of candidate genes for the high-confidence QTLs.

Biotic stress	Trait	QTL name	Candidate genes	Function
Leaf curl	AUDPC and Max	Leaf_curl_4	Prupe.4G026800	LEA hydroxyproline-rich glycoprotein family
			Prupe.4G026900	
			Prupe.4G027000	
			Prupe.4G027300	Cysteine-rich receptor-like protein kinase
			Prupe.4G027400	
Leafhopper	AUDPC	Leafhopper_7_1	Prupe.7G065300	TMV resistance protein N
			Prupe.7G065400	Disease resistance protein (TIR-NBS-LRR class)
			Prupe.7G065500	
Powdery mildew	Max	Powdery_mildew_6	Prupe.6G132100	Disease resistance protein (TIR-NBS-LRR class)
Peach rust	AUDPC	Peach_Rust_2	Prupe.2G057600	TMV resistance protein N
			Prupe.2G057800	
			Prupe.2G058400	Ethylene-activated signaling pathway
			Prupe.2G058400	
			Prupe.2G059000	TMV resistance protein N
			Prupe.2G059200	
			Prupe.2G059400	
			Prupe.2G060200	Ethylene-responsive transcription factor
			Prupe.2G060400	Disease resistance protein (TIR-NBS-LRR class)
	AUDPC	Peach_Rust_4	Prupe.4G051500	Plant-type hypersensitive response
			Prupe.4G053700	MLO-like protein
			Prupe.4G054500	Transcription factor VOZ1
Shot hole	AUDPC	Shot_hole_8	Prupe.8G257400	MLO-like protein
			Prupe.8G259700	Co-chaperone protein
Apricot rust	AUDPC	Apricot_Rust_2	PruarM.2G180700	Leucine-rich receptor-like protein
	AUDPC	Apricot_Rust_3.2	PruarM.3G108000	F-box family protein
	AUDPC	Apricot_Rust_4.2	PruarM.4G199900	Leucine-rich receptor-like protein
	Max	Apricot_Rust_4.4	PruarM.4G204800	
	AUDPC	Apricot_Rust_4.5		
	AUDPC	Apricot_Rust_4.6		
	AUDPC	Apricot_Rust_4.7		
	Max	Apricot_Rust_6.1	PruarM.6G017900	Receptor-like serine/threonine-protein kinase RLK1
	Max	Apricot_Rust_6.2		
Apricot rust Leaf fall	AUDPC and Max	Apricot_Rust_leaf_fall_2.1	PruarM.2G251100	Disease resistance protein RGA2
	Max	Apricot_Rust_leaf_fall_2.2	PruarM.2G283800	TMV resistance protein N
	Max	Apricot_Rust_leaf_fall_2.3	PruarM.2G352900	WD-40 repeat family protein
	Max	Apricot_Rust_leaf_fall_2.4	PruarM.2G359800	Cysteine-rich receptor-like kinase
	Max	Apricot_Rust_leaf_fall_2.5	PruarM.2G359900	
	AUDPC	Apricot_Rust_leaf_fall_4.1	PruarM.4G156300	Lectin protein kinase family protein
	Max and AUDPC	Apricot_Rust_leaf_fall_4.6	PruarM.4G199400	Leucine-rich receptor-like protein
	Max and AUDPC	Apricot_Rust_leaf_fall_4.7	PruarM.4G199700	
	Max and AUDPC	Apricot_Rust_leaf_fall_4.8	PruarM.4G199900	
	Max and AUDPC	Apricot_Rust_leaf_fall_4.10	PruarM.4G201600	BURP domain-containing protein
	Max and AUDPC	Apricot_Rust_leaf_fall_4.16	PruarM.4G204800	Leucine-rich receptor-like protein
	Max and AUDPC	Apricot_Rust_leaf_fall_4.17		
	Max and AUDPC	Apricot_Rust_leaf_fall_4.18		
	Max	Apricot_Rust_leaf_fall_4.21 Apricot_Rust_leaf_fall_4.22	PruarM.4G206100	
	Max and AUDPC			
	Max	Apricot_Rust_leaf_fall_6.1 Apricot_Rust_leaf_fall_6.2 Apricot_Rust_leaf_fall_6.3	PruarM.6G017900	Receptor-like serine/threonine-protein kinase RLK1
	Max and AUDPC			
	Max			
	Max and AUDPC	Apricot_Rust_leaf_fall_6.5	PruarM.6G018400	Receptor-like serine/threonine-protein kinase RLK1

A total of 87 candidate genes have been identified across the 60 high-confidence QTLs, with up to nine candidate genes for the peach rust QTL on chromosome 2 (Table 1). Among the candidate genes, seven were linked to tobacco mosaic virus (TMV) resistance protein, seven to Leucine-rich receptor-like protein kinase family protein, four to cysteine-rich receptor-like protein kinase, four to TIR-NBS-LRR disease resistance protein, and two to MLO-like protein (Table 1). For some QTLs associated to apricot leaf rust, leafhopper, and shot hole no candidate genes could be clearly identified.

Interestingly, three apricot candidate genes, ‘PruarM.6G017900’, ‘PruarM.4G204800’, and ‘PruarM.4G206100’ were identified as covering several QTLs (Table 1). We calculated the local LD in these three zones (Fig. 6C and D, Supplementary Fig. S11) and observed two different patterns: for ‘PruarM.6G017900’ (Fig. 6C) and ‘PruarM.4G204800’ (Supplementary Fig. S11A) a relatively high LD block overlapped precisely with the underlying candidate gene. These results suggested that the QTLs identified in these different genes were not independent. In contrast, ‘PruarM.4G206100’ appeared to be located in a moderate LD region with no clear LD blocks (Supplementary Fig. S11B). Max G-BLUPs distributions according to the haplotype constituted by the top SNPs identified in these three genes were plotted (Fig. 6D, for top SNPs belonging to ‘PruarM.6G017900’ and Supplementary Fig. S11C and D for ‘PruarM.4G204800’ and ‘PruarM.4G206100’). Both genes ‘PruarM.6G017900’ and ‘PruarM.4G204800’, the two most frequent haplotypes, had significantly different G-BLUPs values (Wilcoxon test *P*-value <.05) (Fig. 6D and Supplementary Fig. S11B).

## Discussion

The main objective of this study was to dissect the genetic architecture of a series of traits reflecting the response to natural infections in multiple environments in peach and apricot. We specifically aimed at deciphering G × E interactions, identifying candidate genomic regions, and comparing the results obtained in these two species.

### Biotic stress response is highly influenced by G × E interactions

Our results revealed that the expression of pest and disease susceptibility was under a strong influence of the environment *per se* but also of G × E interactions (Figs 1 and 2, Supplementary Figs S1–S3). Variance decompositions (Fig. 2A) and the pairwise genetic correlations (Supplementary Table S1) indicated the prevalence of the G × E interactions over G effects for several traits. Similar patterns have been found in stone fruit diseases, such as in peach brown rot, where skin tolerance to the fungal infection appears to be under high seasonal influence [14, 16]. In a study on apricot bacterial canker, Omrani *et al.* (2019) pointed to the relevance of controlling G × E interactions for accurately estimating genetic effects in the face of environmental variations in multiyear data [13]. Relying on large experimental and phenotyping efforts, our study is the first to dissect the effects of G × Y and G × L in peach and apricot response to multiple biotic stress. We found that G × E interactions were generally driven more by location than by year effects, which was supported by phenotypic and GWAS analyses. This stronger effect of location might result from the combination of multiple factors such as the local strain of pathogen, the local climate (especially humidity and temperature), or the soil conditions. With single-environment GWAS, we identified a total of 567 environment-specific QTLs and 135 environment-shared QTLs with a higher overlap between QTLs identified from each location than from QTLs identified from a given year. Interestingly, the QTLs identified with the multienvironment models (MTMM and Meta-GWAS) were mostly interactive, which change in direction (‘antagonist’) or intensity (‘differential’) according to the environment. Our results thus indicate that data on specific climatic conditions have the potential to reveal additional regions linked to biotic stress response. We thus recommend evaluating the same panel—or highly overlapping panels including several common genotypes—in multiple geographic areas with contrasted environmental conditions. It should be noted that though single-environment GWAS seem to catch G × E interaction QTLs, they can only do so in the light of a comparison between several single-environment analyses.

Main effect QTLs remain to be validated. While functional validation is difficult in fruit trees [60], empirical validations of resistance/tolerance alleles in different genetic backgrounds by performing additional phenotyping of hybrids intercrossed with accessions bearing favorable alleles is a pragmatic strategy. It should be reminded that the functional validations of G × E interaction QTLs are still rare and requires challenging tests such as gene cloning [61]. Finally, a better characterization of the environments through envirotyping could allow to identify which environmental variables modulate G × L effects (and thus G × E QTLs) and to group locations accordingly for common adapted varieties [24]. Nevertheless, G × Y QTLs are impossible to validate in practice. Overall, while the path to MAS for G × E QTLs seems difficult, quantifying G × E variance and disentangling (true) main effect QTLs from G × E QTLs is a major step to better harness robust QTLs that can be transmitted to the next generation.

### A focus on peach leaf curl and apricot blossom blight

Among the seven biotic stresses studied, leaf curl in peach and blossom blight in apricot stood out from the others by presenting two distinct typologies: the former being little influenced by environments and G × E interactions and the latter, by contrast, being strongly influenced by environmental effects. Our study provides the first GWAS analysis on these two major stone fruit diseases.

Apricot blossom blight variation was found to be controlled by a high number of environment-dependent QTLs, indicating a complex genetic determinism. Variance decompositions and GWAS analyses pointed toward predominant G × Y effects on this disease. In fact, all QTLs identified with single-environment models were year-specific and respectively 65% and 61% of the QTLs identified with MTMM and meta-GWAS presented allelic effects modulated by year (*i.e.* interactive QTLs). Due to these important fluctuations between years, no high-confidence QTLs could be identified with our method. It has been shown that the development of Monilinia on apricot is tightly linked to the interaction between phenology and meteorological conditions, where temperature and humidity during blossom play a major role [62, 63], knowing the petals being the entry point for the fungus [64, 65]. Our dataset was filtered prior to analyses to avoid strong pheno-climatic bias by taking out blossom blight incidence scores for trees having insufficient flower density and Monilinia climatic index for a given year [66]. Despite decent heritability values obtained for apricot blossom blight (0.51; Fig. 2B), we might need to better account for the epidemiological context to further study the genetic basis of that disease.

In contrast to apricot blossom blight, leaf curl damage was a highly heritable trait underlined by low environmental and G × E effects (Fig. 2 and Supplementary Table S1). This low environmental impact could be partly explained by a homogeneous leaf curl pressure across the targeted geographic areas, but also within fields and along the susceptibility period (leaf emergence stage). On the opposite of blossom blight, where the susceptible stage (*i.e.* flowering) lasts for a few days to a week, the infection period for leaf curl lasts over several weeks [67] meaning that phenology-driven avoidance is rare. In line with the phenotypic analyses, we found a highly stable QTL located on chromosome 4 (identified with all GWAS models tested apart from MTMM). Twenty-three further QTLs have been identified for leaf curl, with an intermediate level of confidence, and mostly driven by location-specific effects (19 location-specific QTLs identified with single-environment models). These results suggest that low susceptibility to leaf curl is governed by a major QTL combined with several minor genes having weaker and environment-specific effects.

In terms of experimental design, we would recommend studying blossom blight incidence over several years in a location with overall high incidence (*e.g.* upper Rhone Valley in France) to capture for each accession a season during which the tree was actually confronted with the risk. Regarding leaf curl, several locations might be necessary only to capture the minor-effect genes that act as a complement to the major QTL. In terms of breeding strategies, MAS seems to be particularly well adapted to leaf curl, whereas for blossom blight, combining improved epidemiological risk prediction with genomic selection would be a promising strategy.

For the remaining disease and pests, the phenotypic response appeared to be more nuanced with no strong typology emerging. Although we highlighted a location effect, the relative classification of accessions is relatively well preserved between sites (*i.e.* rank inversions between accessions are not so common). Regarding shot hole, low heritability values might reflect an overall low pressure of that disease combined with low genetic diversity for this trait (we noticed only a few accessions with high susceptibility). It might be necessary to develop a specific biparental population to identify susceptibility alleles in order to avoid passing them by over the breeding process. On average, 23% of the QTLs identified with single-environment GWAS were ‘environment-shared’. Finally, the majority of ‘interactive’ QTLs identified presented a differential profile, which suggest that some QTLs have effects in some environments and not in others, but true inversions of QTL effects are less common.

### Usefulness of AUDPC and Max variables to study biotic stress response: identification of complementary regions

We decided to compare Max and AUDPC, two key metrics to capture the complexity of disease dynamics in naturally infected fruit tree orchards. The maximum of damage score provides a clear indicator of the absolute vulnerability to biotic stresses but overlooks disease progression over time in different epidemiological contexts. In contrast, AUDPC provided more location- or year-specific QTLs, as this variable is strongly related to the number, frequency, and extent of observations performed during the season and thus covers much better the disease progression. Thus, AUDPC values capture more epidemiological information and is thus generally more variable than Max (Fig. 1, Supplementary Fig. S2).

Although we found high correlations between Max and AUDPC for all biotic stresses studied, heritability was slightly higher for AUPDC (Figs 1 and 2B and Supplementary Fig. S2), which probably contributed to the identification of 31–97 new QTLs with that variable (depending on the model used). Importantly, among the high-confidence QTLs, 32% were only detected using AUDPC. Overall, QTLs detected with Max were more stable while those identified with AUDPC were more location- or year-specific. This underlines the complementary nature of the two metrics. Although widely used in other crops, mostly under controlled infection conditions, the use of AUDPC has increased in recent years for assessing quantitative disease resistance in fruit trees and for evaluating disease management practices [68–70]. Some studies have even highlighted the dynamics of genetic effects during pathogen colonization with a distinction between early and late response and temporal pattern in effects of QTLs [69].

Because AUDPC requires a large effort to provide repeated measures at multiple timepoints of the season, we recommend implementing this metric for diseases for which the onset and duration of infection is critical for production, such as apricot rust, or for polycyclic diseases [71]. Indeed, early summer defoliation caused by rust has a strong impact on flowering capacity next year. We showed that among the high-confidence QTLs identified for apricot rust and rust leaf fall, 25% were AUPDC-specific and only 37% overlapped between AUDPC and Max. Therefore, AUDPC enables better distinction of accessions with a high and continuous level of infection at critical timepoints of the season, a feature not possible with Max.

### High-confidence QTLs reveal the role of basal and host-specific resistance shaping the response to biotic stress in stone fruit trees

Among the QTLs detected with single-environment models, respectively 79%, 77%, and 54% were also detected with GWAS model focusing on the main genetic effect, by the multienvironment GWAS (MTMM), and with Meta-GWAS. In total, these different GWAS models enabled the detection of respectively 38, 79, and 29 QTLs that had not been detected with single-environment GWAS. These findings support combining single- and multienvironment models to improve the statistical power, the reliability, and robustness of GWAS. Similar results have been obtained in the case of QTL detection for drought response in maize where the incorporation of the interaction with the environment improved the power of GWAS and enabled to find QTL that are significant in a broad range of environments [72]. By conducting GWAS with multiple methods, we narrowed down the list of relevant regions to a total of 60 high-confidence QTLs across traits. These QTLs explaining in average 36.8% of the genetic variance, and for apricot rust they also explained 53.6% of the genotype by location interaction variance.

To our knowledge, for most of the pests or diseases studied here, resistance or tolerance mechanisms have never been investigated by GWAS analysis nor by QTL mapping, as is the case for leafhopper, shot hole, blossom blight, and peach and apricot rust. Previous analyses on peach powdery mildew have already identified QTLs and candidate genes (Vr2 and Vr3) located on chromosomes 8 and 2 [73–75], but none of them overlap with the high-confidence QTL identified here. The high-confidence QTL we identified for leaf curl on LG4 was not reported previously. A single-linkage analysis study on leaf curl in an interspecific F1 progeny between a peach cultivar and *P. davidiana*, a peach-related species, reported two consistent QTLs located on linkage groups 3 and 6 [76]. QTLs were also identified in these regions by MTMM and Meta-GWAS but they were not categorized as high confidence. In contrast to a QTL-mapping population where the genetic background is homogenous, major alleles might go undetected due to low frequency in a core collection. This may explain the low overlap between our results and those already reported. The type and number of markers included, the approaches used for detecting QTLs, or the difference in phenotyping techniques might also contribute to divergent results between studies, which, taken together, further highlights the complex genetic determinism of those biotic traits.

Importantly, the majority of candidate genes identified belong to the Leucine-rich repeat (LRR)-containing receptors (LRR-CRs) family gene that represents the largest class of plant resistance genes (R genes) [77, 78]. LRR-CRs can be classified in three main subfamilies: LRR receptor-like kinase (LRR-RLK), LRR receptor-like protein (LRR-RLP), and nucleotide-binding site LRR (NBS-LRR) [79, 80]. In our study we identified candidate genes belonging to all the categories with genes related to i) LRR-RLK identified for apricot rust and rust leaf fall (PruarM.6G017900 and PruarM.6G018400), ii) LRR-RLP identified for apricot rust and rust leaf (PruarM.2G180700, PruarM.4G204800, PruarM.4G199400, PruarM.4G199700, PruarM.4G199900, PruarM.4G204800, and PruarM.4G206100), and iii) NBS-LRR identified, for leafhopper (Prupe.7G065400 and Prupe.7G065500), powdery mildew (Prupe.6G132100), and peach rust (Prupe.2G060400). RLKs and RLPs have been identified to function as cell surface pattern-recognition receptors (PRRs), which detect pathogen-associated molecular patterns (PAMPs) and lead to the activation of the pattern-triggered immunity (PTI), the basal defense responses to pathogens [79, 81]. We also identified a high number of genes annotated as ‘TMV resistance protein N’ (Prupe.7G065300 identified for leafhopper; Prupe.2G057600, Prupe.2G057800, Prupe.2G059000, Prupe.2G059200, and Prupe.2G059400 identified for peach rust; and PruarM.2G283800 for apricot rust leaf fall). The tobacco N gene, conferring resistance to TMV, has been identified as TIR-NBS-LRR protein type [82]. It has been shown that NBS-LRR proteins conferred resistance to diverse pathogens, including fungi, oomycetes, bacteria, viruses, and insects. By recognizing pathogen effector molecules, NBS-LRR proteins lead to the activation of defense responses and play a fundamental role in the effector-triggered immunity (ETI), the second pathogen-sensing mechanism in plants [81]. ETI usually induces programmed cell death at the infection site, known as the hypersensitive response (HR), and thus locally limits pathogen spread. NBS-LRR proteins are highly variable and subject to balancing selection, which could contribute to the high diversity and specificity of these effectors [79]. Thus, our results point toward a prevalent role of host-specific (narrow spectrum) and nonspecific (large spectrum) resistance mechanisms among the diseases studied here. In *Prunus*, other studies have shown the role of LRR-CRRS in plant defense. As such, The *Ma* gene, conferring complete-spectrum resistance to root-knot nematodes (RKNs) was the first TIR_NBS_LRR (TNL) gene from *Prunus* to be cloned [83]. As for them, the *Rm* genes conferring resistance to green peach aphids encode TLN proteins [84]. Recently, Marimon *et. al* identified a candidate gene belonging to the RGA genes—NBS-LRR analogs involved in the recognition and prevention of plant pathogens [85]—for the peach powdery mildew resistance gene Vr3 [74].

### Similar, yet divergent: difference in the architecture of biotic stress response between peach and apricot in the light of their biological features

Relying on similar experimental designs (dense set of genome-wide markers, multisite orchards, phenotyping methods) and on the same analytic pipeline used for both species, our results allow for a coherent comparison of results obtained in peach and apricot. Strikingly, we found a much larger number of QTLs in apricot than in peach, and observed also more large-effect QTLs in peach than in apricot, exemplified with results on leaf curl and blossom blight. These differences might owe to distinct biological features. Indeed, we know that peach and apricot are closely related species, yet they present different reproductive characteristics and demographic history [86, 87]. Endowed with a self-incompatibly system, apricot is a cross-pollinating species [88], whereas peach is a preferentially self-pollinating species [89]. This results for apricot in a high number of effective recombination events and a rapid expected LD decay over the genome, whereas the opposite pattern is expected for peach [90]. In our study, global mean LD blocs over all chromosomes are ~3300 times longer for the peach core collection compared to the apricot core collection. In fact, previous association studies have already highlighted an LD decay over very short distances (100–200 bp) in apricot (based on a population comprising an important part of the material used in presented work) [12, 13]. In a similar way for peach, other studies have found a significant LD decay at 5 kb–1.8 Mb (depending on the population), confirming high levels of LD over long distance for that crop [41, 91]. QTL intervals were therefore particularly small for apricot (max 946 bp), with most of them being defined by the top SNP. Minding a sufficient number of markers, a higher mapping resolution makes association analyses more powerful to target causal polymorphisms in apricot, facilitating the identification of promising candidate genes [92]. For rust tolerance, we identified three genes covering several confidence QTLs in apricot, along with favorable haplotypes (Fig. 6D and Supplementary Fig. S11). Results on apricot strongly contrast with those obtained in peach, where QTLs were up to max 3240 kb in size, and contained 52–528 genes. Using local LD values allowed to refine QTL sizes and to narrow down the list to 23 candidate genes. Overall, the contrasting biological features and demographics of peach and apricot seem to have a strong impact on the genetic architecture of biotic traits in these two crops.

In our study, rust was the only disease able to affect both peach and apricot. Rust diseases have a fungal origin and affect many crops, and typically present a high specificity to their host [93]. *Tranzchelia discolor* (Fuckel) has been shown to attack peach, almond, plum, apricot, and cherry [94, 95]. For both species, a high-confidence QTL was identified on chromosomes 2 and 4. Although the QTLs do not colocalize, they are found in the same chromosome region. Further comparative genomics work was out of scope, but could be undertaken using synteny approaches, such as found in a recent study in the apricot–peach–plum–mei complex [87].

Finally, we identified a total of 123 new QTLs using non-additive models, indicating that non-additive effects play a significant role in biotic stress response for both species. In proportion, more non-additive QTLs were found in peach (56%) than in apricot (39%). Although peach is a self-compatible species with a much low level of heterozygosity compared to apricot [87], this result indicates that both additive and non-additive genetic effects are important in the genetic architecture of the traits under study, as has already been shown for leaf curl susceptibility inheritance [51]. In almond, another cross-pollinating *Prunus* species, non-additive GWAS conducted on agronomic traits led to the identification of 13 QTLs with only one having additive effect [86]. Harnessing non-additive variance might thus be valuable to better exploit genetic variance, which is particularly relevant in clonally propagated crops.

## Conclusion and prospects

This study provides a thorough characterization of the genetic variation underlying resistance and tolerance to several pests or diseases in peach and apricot via observations over multiple environments under natural conditions. By quantifying and exploiting G × E interactions, we found a majority of environment-specific or interactive QTLs. For future experiments, we recommend considering the combination of pathogens that are specifically expressed in each target location to optimize the choice of experimental sites.

Our results emphasize that, while accounting for pedo–climatic adaptation and market requirements, future breeding should focus on adaptation to the biotic profile of the target production area. This might call for the design of target populations of environments (TPE), a common strategy in big crops [96], which could be adapted to distinguish between major biotic environments in stone fruit. Developing new peach and apricot cultivars is a time-consuming process because of the long juvenile stage, large plant size, and the multiyear recording of phenotypic performance [97]. As a next step, the effects of the allelic variants identified in this study need to be validated before incorporating them in MAS strategy to efficiently screen breeding materials and optimize the multitrait and multienvironment selection schemes. Another promising strategy to overcome the limiting aspects of GWAS would be the use of genomic prediction models for a better exploitation of weak effects underlying quantitative resistances and tolerances [98, 99].

Our study highlights the highly contrasted genetic architectures of the seven traits studied. A promising approach could be to target in priority highly heritable traits and to use resulting GWAS-identified QTLs in the early stages of a breeding program, in order to enrich the material with high-effect alleles. In a second stage, for less heritable traits, to use genomic selection to for the exploitation of low-effect loci, which are difficult to detect in GWAS. In any case, considering other protective levers such as prophylaxis will remain crucial to achieve, as a whole, more sustainable fruit production.

## Material and methods

### Plant material and trial description

The plant material and experimental design used in this study were previously described in [100]. To sum up, we used two core collections of respectively 206 peach (*P. persica and 3 relatives: P. davidiana, Prunus mira,* and *Prunus kansuensis*) and 150 apricot (*P. armeniaca*) accessions coming from multiple geographical origins with significant number of accessions of historical and regional importance, landraces, and elite materials. The peach core collection also includes ornamental cultivars, few accessions from closely related species, and interspecific crosses.

All orchards are located in South-East France, in three locations for peach: Étoile-sur-Rhône (44.8°N/ 4.9°E, Drôme), Avignon (43.9°N/4.8°E, Vaucluse), and Torreilles (42.8°N/3.0°E, Pyrénées-Orientales), respectively called ET, AV, and TO; and in two different locations for apricot: Saint-Marcel-lès-Valence (45.0°N/4.9°E, Drôme) and Bellegarde (43.7°N/4.5°E, Gard), respectively called SM and BE. Climate ranges from Mediterranean to semicontinental. Peach and apricot orchards are composed of two and five randomized complete blocks, respectively, each block containing one replicate per genotype. All accessions have been grafted on ‘Montclar® Chanturgue’ peach rootstocks. First leaf emergence was in 2019 for peach and in 2018 for apricot.

The orchards were managed under integrated plant protection during the first 2 years after the plantation with a progressive reduction of phytosanitary protection thereafter. Overall, only punctual treatments were applied if the survival of the orchard was at stake for peach orchards since 2023, and except for the protection against the psyllid vector of the Apricot Chlorotic Leaf Roll (ACLR) in Bellegarde, no protection was applied for apricot orchards since 2020.

### Phenotypic data collection

We monitored the damage caused by four diseases and one pest on peach in 2021, 2022, and 2023 and by two diseases on apricot in 2020, 2021, 2022, and 2023 (Table 2). For the rest of the analysis, the term of environment refers to the combination of a location and a year. All field data were collected using the ‘Field Book’ Android application [101].

**Table 2:** Summary of pests and diseases monitored in this study.

	Pest or disease	Type of organism	Pathogen	Organ	Number of environments observed	Number of environments retained
Peach	Leaf curl	Fungi	*Taphrina deformans*	Leaves	3 (3 locations and 1 year	3 (3 locations and 1 year)
	Leafhopper	Insect	*Asymmetrasca decedens*	Leaves	6 (3 locations and 3 years)	6 (3 locations and 3 years)
	Powdery mildew	Fungi	*Podosphaera pannosa*	Leaves	8 (3 locations and 3 years)	5 (2 locations and 3 years)
	Rust	Fungi	*Tranzchelia* spp.	Leaves	6 (2 locations and 3 years)	5 (2 locations and 3 years)
	Shot hole	Fungi	*Wilsonomyces carpophilus*	Leaves	9 (3 locations and 3 years)	4 (3 locations and 2 years)
Apricot	Blossom and twig blight	Fungi	*Monilinia* spp. (mostly *Monilia* laxa)	Flowers and twigs	4 (1 location and 4 years)	4 (1 location and 4 years)
	Rust	Fungi	*Tranzschelia* spp.	Leaves	6 (2 locations and 4 years)	6 (2 locations and 4 years)

The assessment of the damage caused by all these pests and diseases was based on the visual estimation of symptom incidence (percentage of damaged leaves). Different rating scales were used to best capture the phenotypic expression of each pest or disease (Supplementary Method 1).

Except for blossom blight, which was assessed ~30 days after blooming date and for which the shoots infected by *Monilinia* spp. were removed, repeated scoring was undertaken for the different pests and diseases to monitor the evolution of contamination throughout the seasons in the different orchards.

### Genotyping and resequencing

Genotyping of the peach core collection was performed on 192 individuals using the higher density IRSC 16 K SNP array. This array was designed to represent genome-wide and species-wide diversity while including known genetic polymorphism linked to agronomic traits (https://www.rosaceae.org/Analysis/431). The genotyping dataset was filtered to retain SNPs with call rate per marker >90%, missingness per individual <50%, heterozygosity $\le$98%, and MAF >5%, which resulted in a final set 13 680 markers.

The apricot core collection was sequenced with the Illumina HiSeq 2000 NGS technique. The sequence alignment was performed on the third version of the ‘Marouch’ genome [38] and SNPs were recovered in a vcf format. This dataset was first filtered to retain only SNPs with a missingness per individual <50% and only biallelic SNPs with >10 reads deep/SNP have been conserved. Finally, the dataset was filtered to retain SNPs with call rate per marker >95%, heterozygosity $\le$98%, minor allele frequency (MAF) >5%, and a removal of duplicated SNPs. This dataset consisted of 207 000 markers.

For both datasets, the imputation of missing data was done using the most frequent allele.

For the datasets used in non-additive GWAS, we included two more filtering steps to retain SNPs with three genotypic classes and with a minimum genotypic class frequency >5%. These datasets consisted respectively of 10 082 markers for peach and 25 508 markers for apricot.

### Statistical analysis of phenotypic data

All statistical analyses of phenotypic and genotypic data were performed using the R software version 4.1.2 (R-Core-Team, 2020).

At each date of observation, phenotypic data were adjusted within each environment to correct for spatial environmental effects using information on row and column (Supplementary Method 2). After removing environments with insufficient phenotypic expression, we extracted Max and AUDPC (Supplementary Method 2). These variables were transformed using Box–Cox to correct for heteroscedasticity and non-normality of error terms (Supplementary Method 2). Variance analysis was performed using mixed linear models to quantify the variance associated to genetic, G × E, and non-additive effects (Supplementary Method 3 and Supplementary Method 4).

### Linkage disequilibrium

Intrachromosomic LD was measured with the *r*^2^ value estimated by using VCFTools v0.1.16 [102]. The *r*^2^ was calculated for every combination of SNPs within a sliding window of 10 Mb for peach and of 30 kb for apricot for every chromosome individually. We used a threshold of 0.2 to set the LD decay, which was then represented graphically using Hill and Weir equation [103].

### Genome-wide association studies

Across-environment GWAS based on main additive and non-additive genetic as well as single-environment GWAS (Table 3) were performed using the single-locus linear mixed model of [104] implemented in the R package MM4LMM [105]:


(1) \begin{equation*} Y= X\beta + Q\upsilon + Zu+\varepsilon \end{equation*}

where $Y$ is respectively the vector of G-BLUPs for across-environment GWAS or of environment-specific BLUPs for single-environment GWAS, $X$ is the molecular marker score matrix,$\beta$ is the vector of marker effects, $Q$ is the structure matrix estimated by the SNMF R package [106] (Supplementary Method 5 and Supplementary Result 1),$\upsilon$ is the fixed effect of the structure, $Z$ is an incidence matrix, $u$ is the vector of random background polygenic effects with variance σ^2^_u_ = K σ^2^_G_ (where K is the variance–covariance matrix of G, and σ^2^_G_ is the genetic variance), and ε is the vector of residuals.

**Table 3:** Summary of the different GWAS model used in this study.

**GWAS model name**	**R Package used**	**Objective of the model**
Main genetic effect additive GWAS	MM4LMM	Decipher the main additive genetic effects across environments
Main genetic effect nonadditive GWAS	MM4LMM	Decipher the main non-additive genetic effect across environments
Single-environment GWAS	MM4LMM	Study genetic effects environment by environment
Multienvironment GWAS	MTMM	Assess the effect of G × E interactions on QTL detection and identify genetic factors with common effects, *i.e.* stable marker effects across environments, or interaction effects, *i.e.* differential marker effects across environments
Meta-analysis GWAS	MetaGE	Assess the effect of G × E interactions on QTL detection by a joint analysis of single-environment GWAS results

The estimation of the variance–covariance matrix of G was determined by a genetic relatedness (or kinship) matrix, derived from all SNPs except those on the chromosome containing the SNP being tested as in [107]. According to the model, additive or non-additive kinships were used. They were calculated using the method developed by [108] and the source code published by [109]. For every pest or disease, we tested two different corrections including the kinship (model ‘K’) or both kinship and population structure (model ‘Q + K’). The best model was chosen on a trait-by-trait basis by comparing the likelihood of each null model (*i.e.* with only kinship of kinship + structure effects) using the AIC. We used a ‘modified’ 5% Bonferroni correction described by [110] to define significant associations between phenotypic data and genotypic markers. This threshold is based on the effective number of independent SNPs calculated thanks to the *Meff* equation implemented in the R package poolR [111]. The number of effective markers was 1326 for peach and 1778 for apricot.

To explore non-additive genotype–phenotype associations, we transformed the genotypic datasets following [53, 54], as illustrated in Supplementary Table S4.

Subsequently, we performed single-locus multienvironment GWAS by using the MTMM as described by [26] in order to distinguish common and environment-specific marker–phenotype association (Table 3 and Supplementary Method 6). We finally performed a meta-analysis relying on the results of the single-environment GWAS model by using MetaGE R package [33] (Table 3 and Supplementary Method 7).

### Identification of high-confidence QTLs and variance analysis of G × E and QTL × E

For all GWAS analyses, every QTL region was defined using the position of the top SNP (*i.e.* with the lowest *P*-value) and the estimated LD decay for every chromosome. In other words, QTLs were centered on the top SNP position with an extent of two time-specific chromosomal LD decay. Candidate SNPs distant less than the LD decay’s size were considered as belonging to this QTL. Each QTL name was defined as the associated trait name, followed by the chromosome number and the QTL’s order among all QTLs for this trait along the chromosome.

For every trait, we selected ‘high-confidence’ QTLs that correspond to QTLs significant in at least 50% of the different additive GWAS models tested for the maximum of damage score or for AUDPC. We considered each environment of the single-environment GWAS as separate models as well as the model ‘full’, ‘common’, and ‘gei’ of MTMM.

To assess the biological relevance of the high-confidence QTLs, we followed the approach previously described by [24, 112], which aim at comparing the variance of a multilocus multienvironment mixed model with and without the fixed effects of the QTLs and of the QTLs by environment interaction (Supplementary Method 8). Last, we calculated the indicator ${\gamma}_{q,r}$ to assess the proportion of genetic, G × L interaction, and G × Y interaction variance explained jointly by all detected QTLs for each trait (Supplementary Method 8).

### Candidate gene analysis

Each high-confidence QTL’s region was queried in the *Prunus persica* Whole Genome v2.0 Assembly and Annotation v2.1 [36] or in the in the *Prunus armeniaca* Marouch whole genome v1.0 [38] from the Genome Database for Rosaceae (www.rosaceae.org). SNP gene localization and function were determined using the JBrowse tool (www.rosaceae.org/tools/jbrowse). Their function was then investigated in the literature, and genes involved in traits linked to host defense mechanisms were therefore proposed as putative candidate genes.

For peach, the local LD was calculated between the top SNP of the different ‘high-confidence’ QTLs and all the SNPs belonging in these QTLs by using LDcorSV R package, which takes into account population stratification and the relatedness between genotypes [113]. We used *r*^2^ > 0.2 to filter the putative candidate genes.

For genes that overlapped among several confidence QTLs for apricot, pairwise LD from all physically close SNPs in the region was computed using *r*^2^ calculated with LDcorSV R package [113]. The haplotypes were constructed by considering all top SNPs of the confidence QTLs involved for each G-BLUPs phenotype. Mean phenotypic distributions of the different haplotypes were compared using paired samples Wilcoxon tests for the associated G-BLUPs phenotypes.

## Declaration of Generative Al and Al-assisted technologies in the writing process

The authors declare that they used ChatGPT (OpenAI) to help in rephrasing the abstract and the introduction during the review process. They carefully revised the output of this service and declare taking full responsibility for the entire content of this publication.

## Supplementary Material

Web_Material_uhaf088

## Data Availability

The raw phenotypic and genotypic datasets and the R scripts generated for this study are accessible on the data.gouv public repository at: https://doi.org/10.57745/2HNRN0
