# Supplementary material for: Multi-environment GWAS uncovers markers associated to biotic stress response and genotype-by-environment interactions in stone fruit trees
Source: Hortic Res. 2025 Apr 22;12(7):uhaf088. doi: 10.1093/hr/uhaf088 (PMC12064953; doi:10.1093/hr/uhaf088)
Supplement: Web_Material_uhaf088 [file web_material_uhaf088.zip › Supplementary_Material_Serrie_GWAS_biotic_stress_Prunus.docx]

Supplementary Material

Article Title: **Multi-environment GWAS uncovers markers associated to biotic stress response and genotype-by-environment interactions in stone fruit trees**

Marie Serrie^1^, Vincent Segura^2,3^, Alain Blanc^4^, Laurent Brun^5^, Naïma Dlalah^1^, Frédéric Gilles^1^, Laure Heurtevin^1^, Mathilde Le-Pans^5^, Véronique Signoret^1^, Sabrina Viret^1^, Jean-Marc Audergon^1^, Bénédicte Quilot^1^, Morgane Roth^1*^


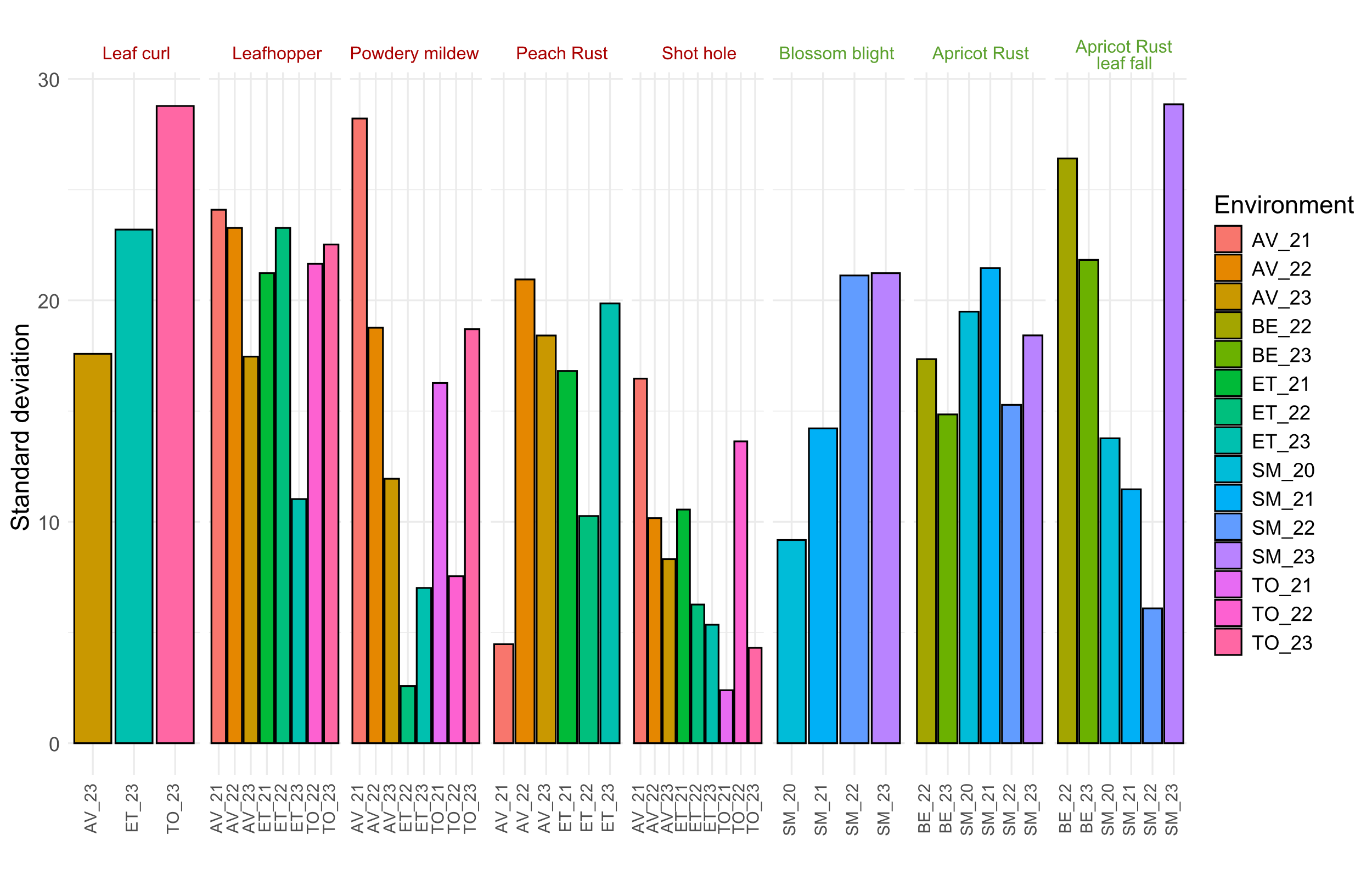


**Supplementary Fig 1.** Standard deviation of Max after spatial correction for the different biotic stresses and in each environment.

*AV: Avignon, ET: Étoile-sur-Rhône, TO: Torreilles, SM: Saint-Marcel-lès-Valence, BE: Bellegarde, 20 : 2020, 21 : 2021, 22 : 2022, 23 : 2023.*

*On the top of the graph, red names are for peach diseases and green for apricot diseases.*


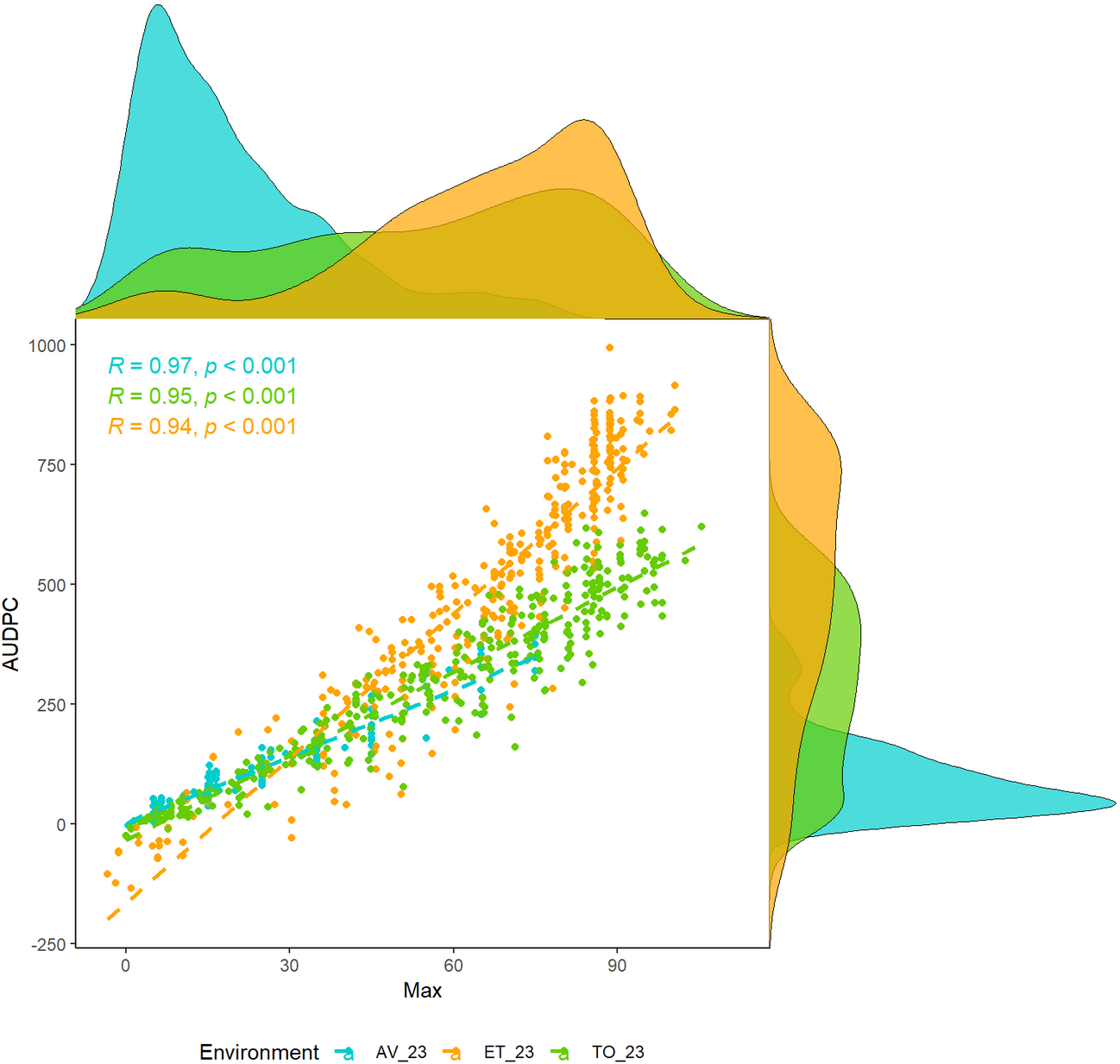

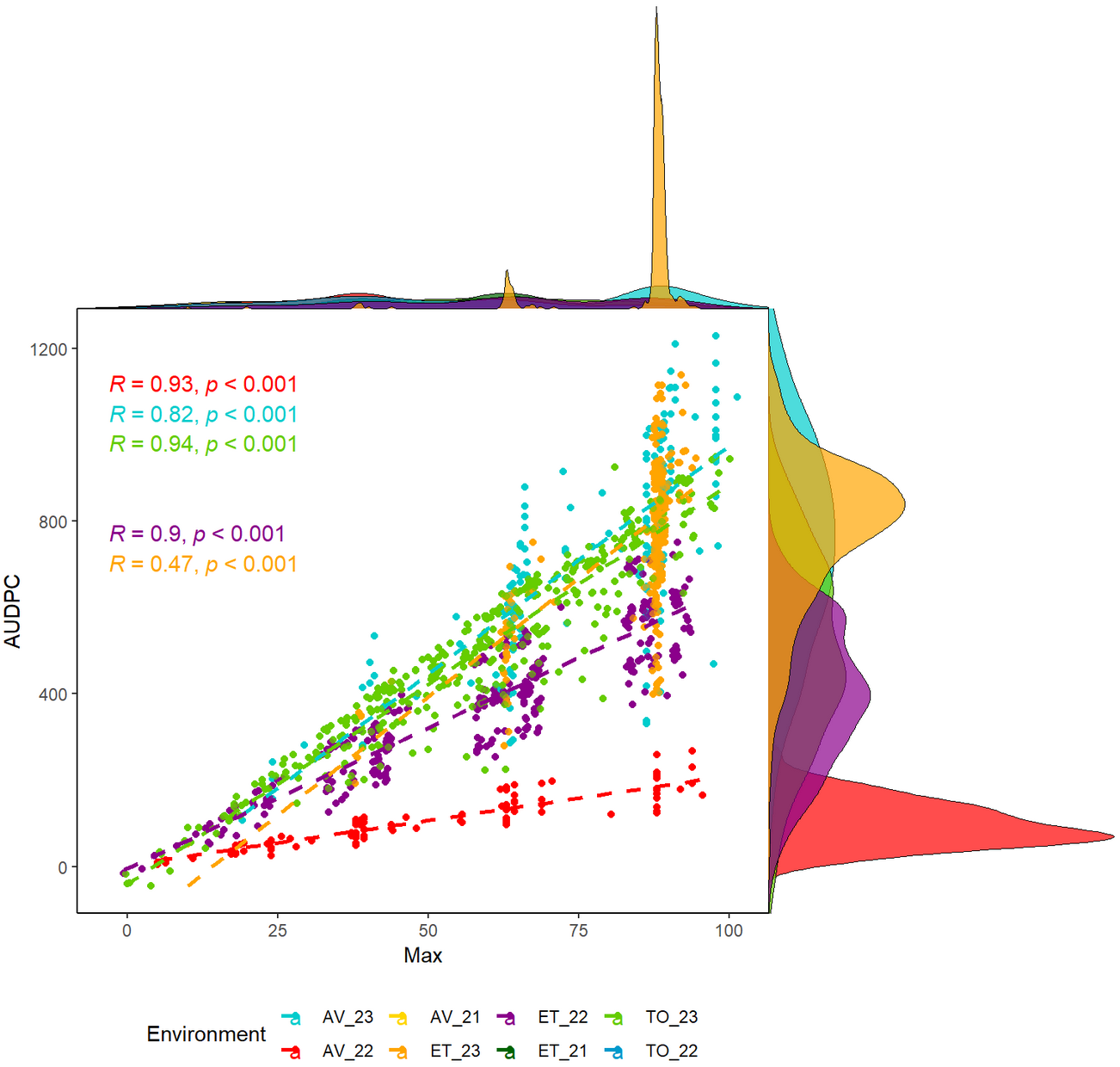

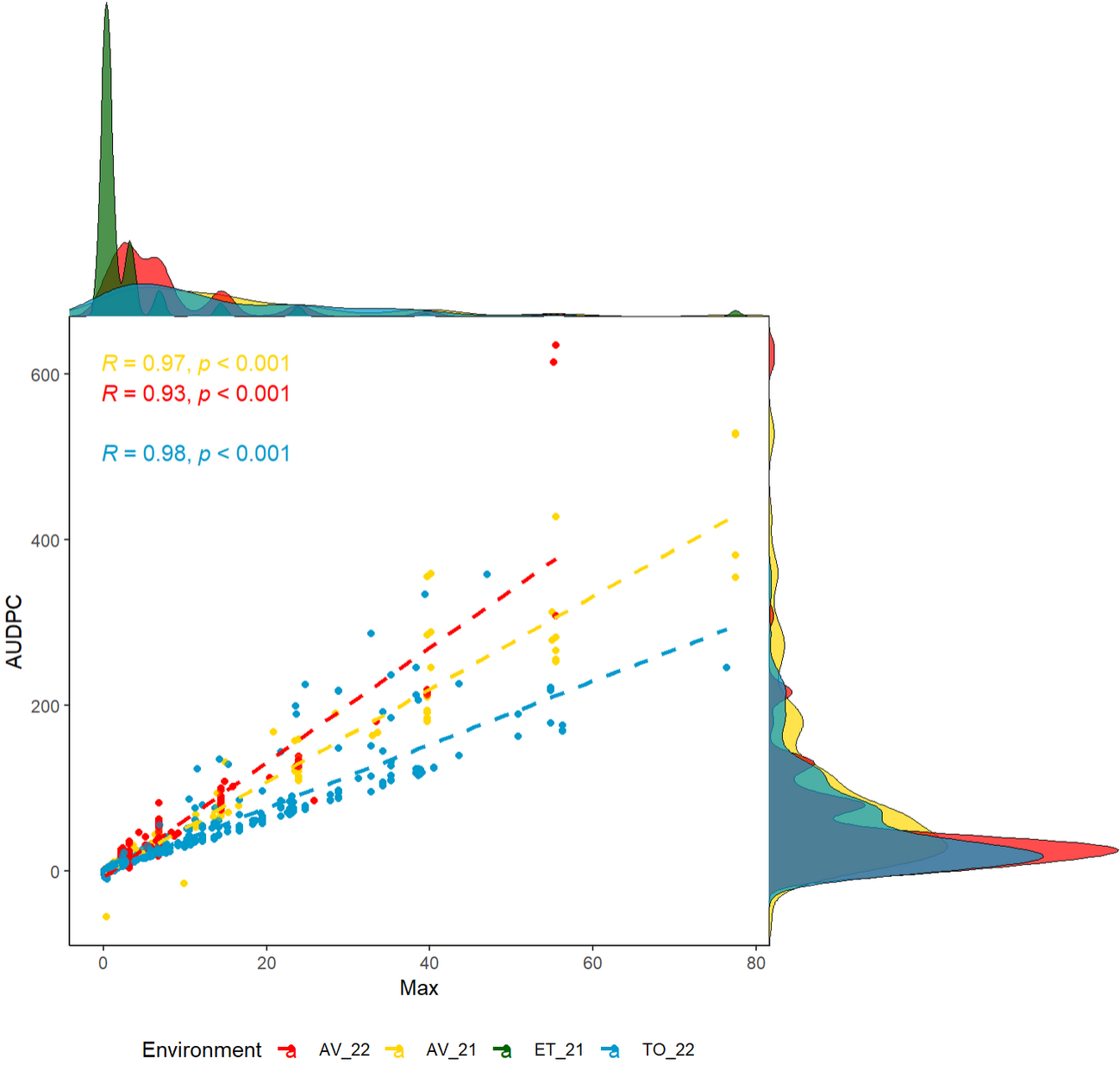

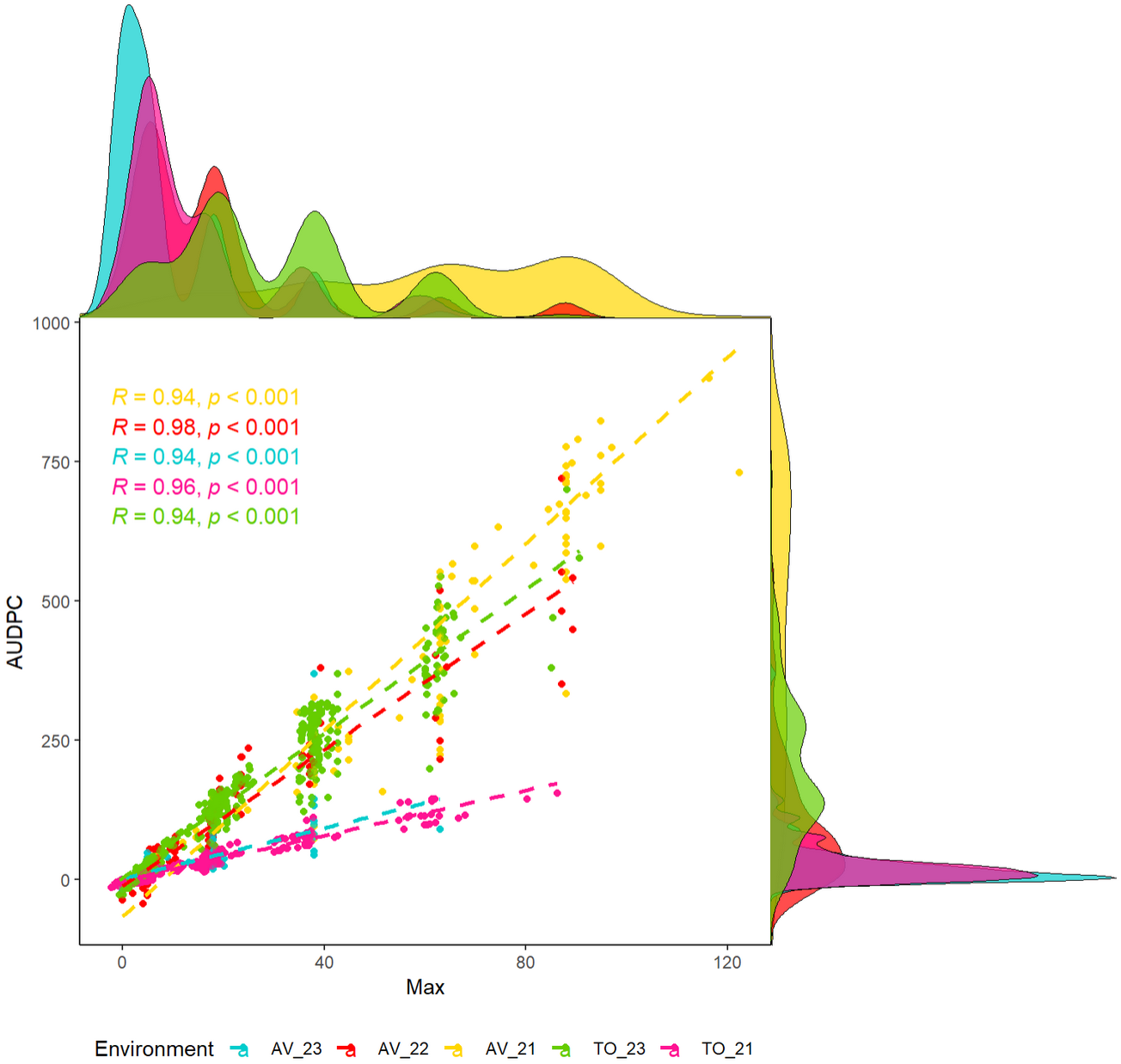


**A**

**B**als

**C**als

**D**als


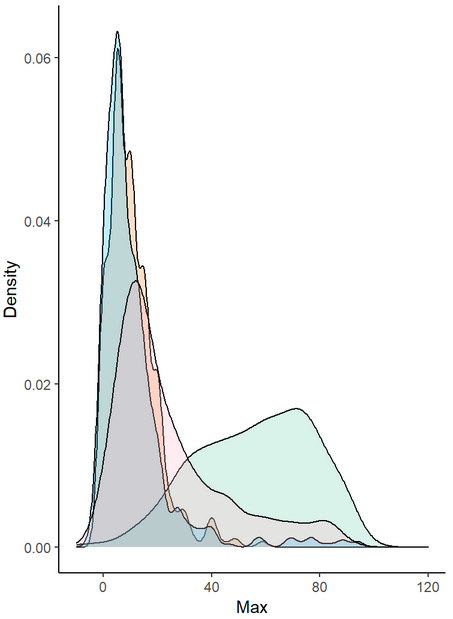

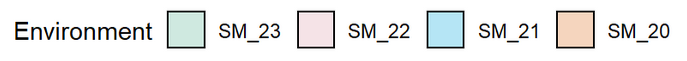

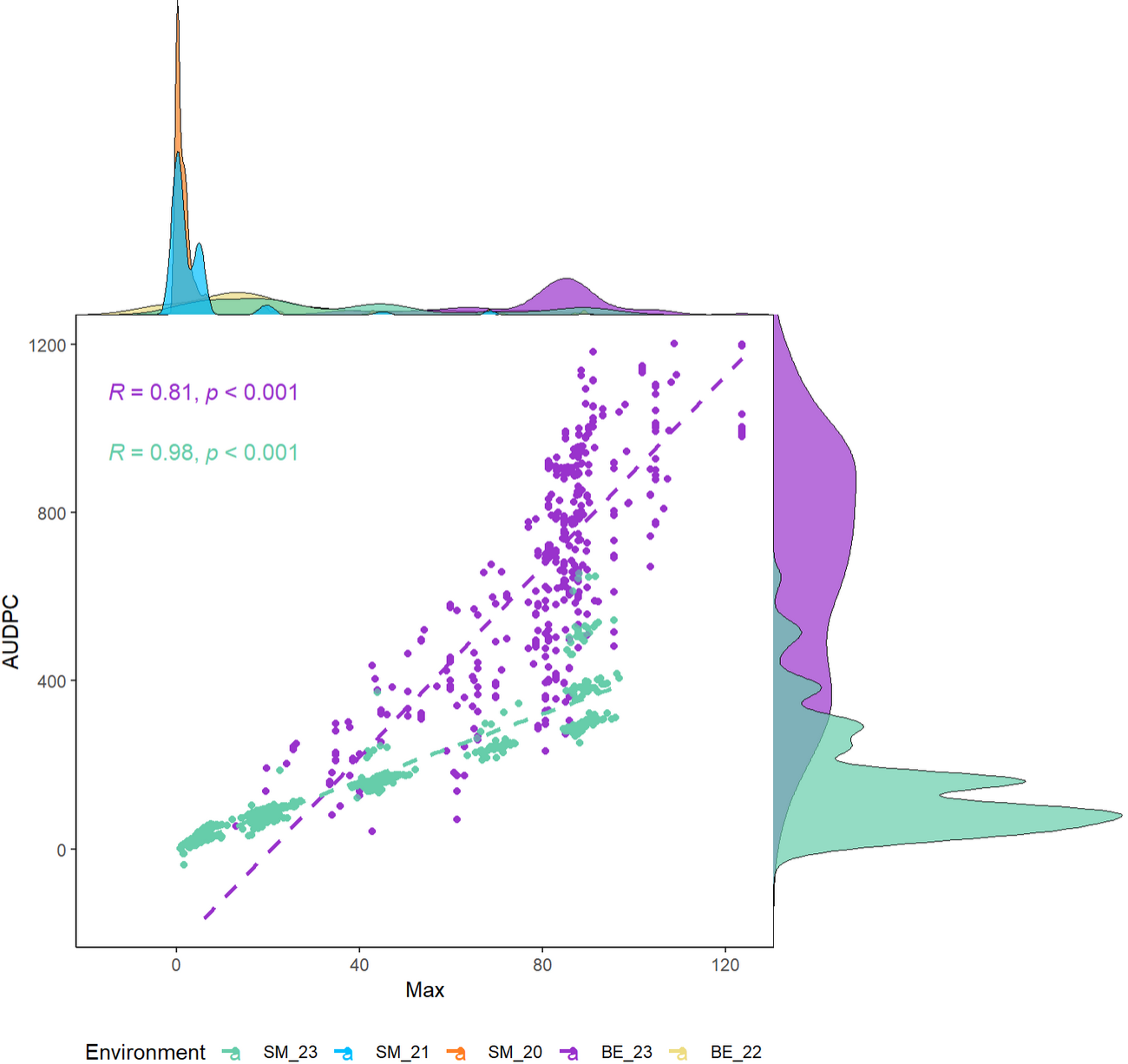


**E**als

**F**als

**Supplementary Fig 2**. Distributions and phenotypic correlation between Max and AUDPC for A) leaf curl B) leafhopper C) powdery mildew D) shot hole E) apricot rust leaf fall and F) Blossom blight (distribution only). Distributions of Max are shown at the top of each graph, while the distributions of AUDPC are shown on the right.

*AV: Avignon, ET: Étoile-sur-Rhône, TO: Torreilles, SM: Saint-Marcel-lès-Valence and BE: Bellegarde.*

**A**

**B**


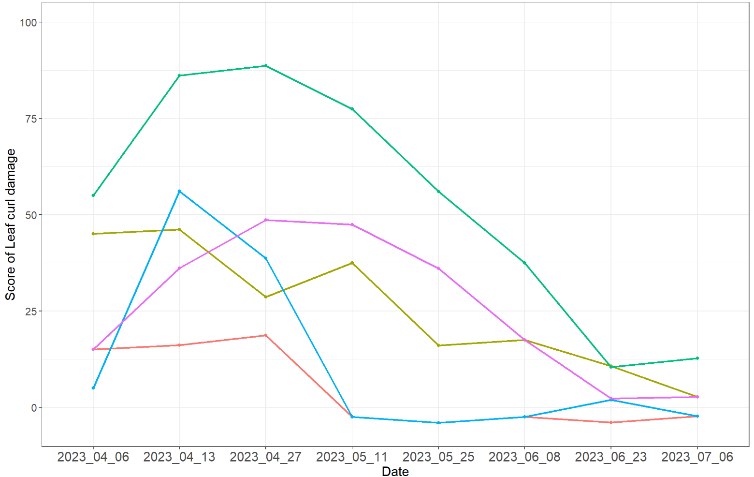


ET_23


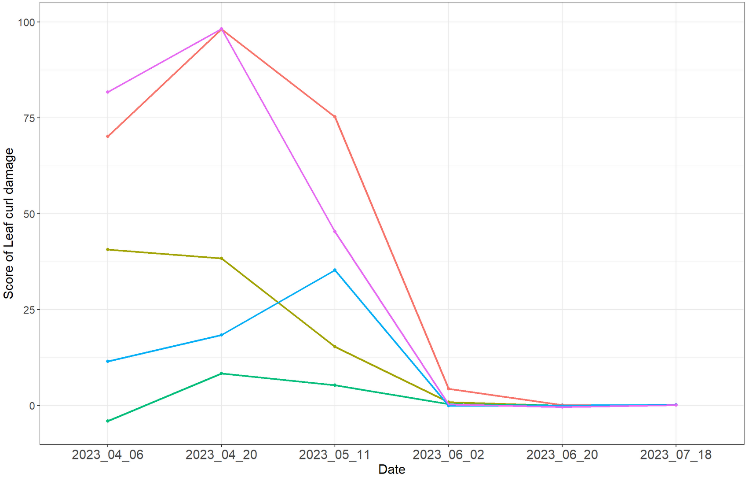


TO_23

BE_23

SM_23


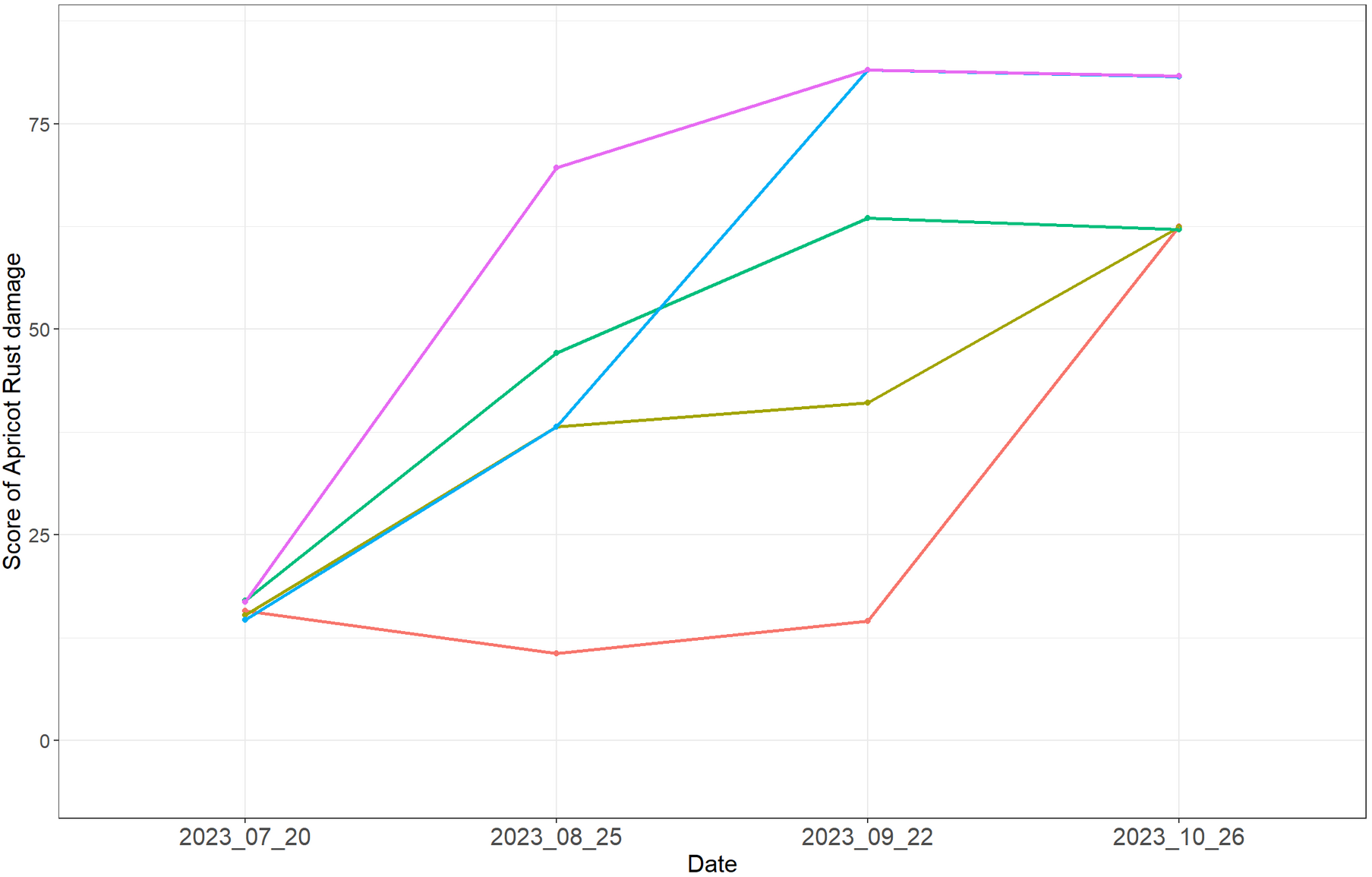

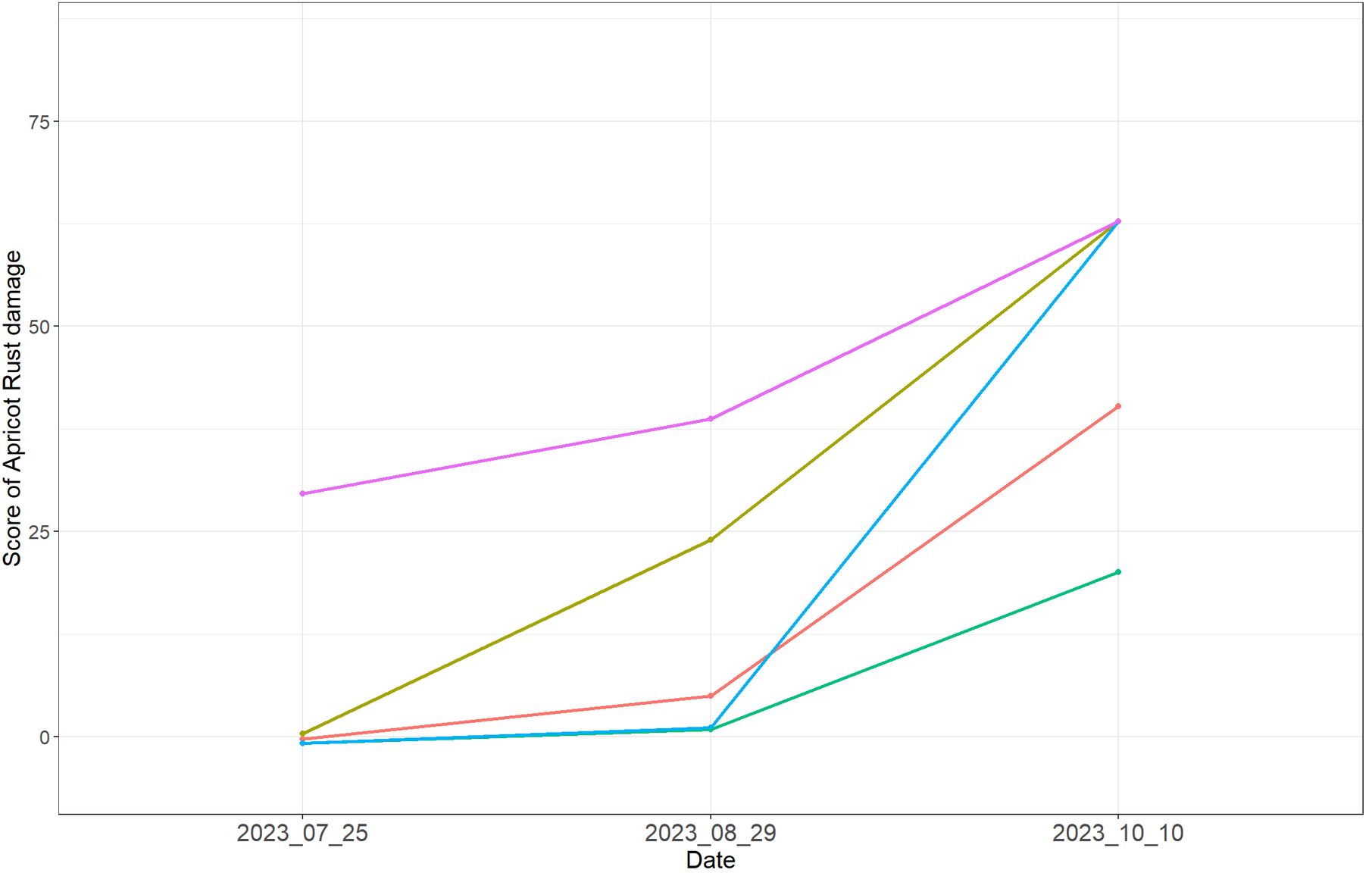


**Supplementary Fig 3**. Evolution of the score of A) leaf curl damage in the orchards of Etoile-sur-Rhône (ET) and Torreilles (TO) in 2023 and of B) apricot rust damage in the orchards of Bellegarde (BE) and Saint-Marcel-Les-Valence (SM) in 2023. These damage scores are adjusted for spatial heterogeneity within each environment and presented for five randomly selected trees.

**Supplementary Table 1.** Genetic correlations of environment-specific BLUPS of Max and AUDPC between the different environments.

*AV: Avignon, ET: Étoile-sur-Rhône, TO: Torreilles, SM: Saint-Marcel-lès-Valence and BE: Bellegarde*

| Biotic stress | Trait |  | Genetic correlation | | | | | | | Mean |
| --- | --- | --- | --- | --- | --- | --- | --- | --- | --- | --- |
| Leaf curl | Max |  | ET_23 | TO_23 |  |  |  |  |  |  |
|  |  | AV_23 | 0.879 | 0.875 |  |  |  |  |  | 0.873 |
|  |  | ET_23 |  | 0.867 |  |  |  |  |  |  |
|  | AUDPC |  | ET_23 | TO_23 |  |  |  |  |  |  |
|  |  | AV_23 | 0.920 | 0.907 |  |  |  |  |  | 0.913 |
|  |  | ET_23 |  | 0.911 |  |  |  |  |  |  |
| Leafhopper | Max |  | AV_22 | AV_23 | ET_21 | ET_22 | ET_23 | TO_22 | TO_23 |  |
|  |  | AV_21 | 0.773 | 0.755 | 0.651 | 0.523 | 0.549 | 0.570 | 0.558 | 0.662 |
|  |  | AV_22 |  | 0.801 | 0.513 | 0.732 | 0.600 | 0.752 | 0.622 |  |
|  |  | AV_23 |  |  | 0.481 | 0.589 | 0.751 | 0.586 | 0.758 |  |
|  |  | ET_21 |  |  |  | 0.676 | 0.705 | 0.594 | 0.561 |  |
|  |  | ET_22 |  |  |  |  | 0.784 | 0.811 | 0.651 |  |
|  |  | ET_23 |  |  |  |  |  | 0.627 | 0.766 |  |
|  |  | TO_22 |  |  |  |  |  |  | 0.785 |  |
|  | AUDPC |  | AV_23 | ET_22 | ET_23 | TO_23 |  |  |  |  |
|  |  | AV_22 | 1 | 0.875 | 0.871 | 0.886 |  |  |  | 0.902 |
|  |  | AV_23 |  | 0.876 | 0.873 | 0.883 |  |  |  |  |
|  |  | ET_22 |  |  | 1 | 0.875 |  |  |  |  |
|  |  | ET_23 |  |  |  | 0.881 |  |  |  |  |
| Powdery mildew | Max |  | AV_22 | AV_23 | TO_21 | TO_23 |  |  |  |  |
|  |  | AV_21 | 0.997 | 0.996 | 0.544 | 0.565 |  |  |  | 0.726 |
|  |  | AV_22 |  | 0.997 | 0.535 | 0.553 |  |  |  |  |
|  |  | AV_23 |  |  | 0.530 | 0.549 |  |  |  |  |
|  |  | TO_21 |  |  |  | 0.990 |  |  |  |  |
|  | AUDPC |  | AV_22 | AV_23 | TO_21 | TO_23 |  |  |  |  |
|  |  | AV_21 | 0.906 | 0.901 | 0.337 | 0.516 |  |  |  | 0.604 |
|  |  | AV_22 |  | 0.918 | 0.346 | 0.475 |  |  |  |  |
|  |  | AV_23 |  |  | 0.300 | 0.432 |  |  |  |  |
|  |  | TO_21 |  |  |  | 0.908 |  |  |  |  |
| Peach rust | Max |  | AV_23 | ET_21 | ET_22 | ET_23 |  |  |  |  |
|  |  | AV_22 | 0.807 | 0.486 | 0.633 | 0.503 |  |  |  | 0.708 |
|  |  | AV_23 |  | 0.592 | 0.649 | 0.704 |  |  |  |  |
|  |  | ET_21 |  |  | 0.904 | 0.900 |  |  |  |  |
|  |  | ET_22 |  |  |  | 0.905 |  |  |  |  |
|  | AUDPC |  | AV_23 | ET_21 | ET_22 | ET_23 |  |  |  |  |
|  |  | AV_22 | 0.944 | 0.550 | 0.612 | 0.590 |  |  |  | 0.704 |
|  |  | AV_23 |  | 0.465 | 0.499 | 0.530 |  |  |  |  |
|  |  | ET_21 |  |  | 0.950 | 0.945 |  |  |  |  |
|  |  | ET_22 |  |  |  | 0.953 |  |  |  |  |
| Shot hole | Max |  | AV_22 | ET_21 | TO_22 |  |  |  |  |  |
|  |  | AV_21 | 0.930 | 0.662 | 0.550 |  |  |  |  | 0.668 |
|  |  | AV_22 |  | 0.646 | 0.542 |  |  |  |  |  |
|  |  | ET_21 |  |  | 0.677 |  |  |  |  |  |
|  | AUDPC |  | AV_22 | TO_22 |  |  |  |  |  | 0.435 |
|  |  | AV_21 | 0.873 | 0.221 |  |  |  |  |  |  |
|  |  | AV_22 |  | 0.212 |  |  |  |  |  |  |
| Blossom blight | Max |  | SM_21 | SM_22 | SM_23 |  |  |  |  |  |
|  |  | SM_20 | 0.645 | 0.404 | 0.548 |  |  |  |  | 0.411 |
|  |  | SM_21 |  | 0.102 | 0.287 |  |  |  |  |  |
|  |  | SM_22 |  |  | 0.478 |  |  |  |  |  |
| Apricot rust | Max |  | BE_23 | SM_20 | SM_21 | SM_22 | SM_23 |  |  |  |
|  |  | BE_22 | 0.731 | 0.715 | 0.749 | 0.697 | 0.739 |  |  | 0.725 |
|  |  | BE_23 |  | 0.638 | 0.609 | 0.550 | 0.717 |  |  |  |
|  |  | SM_20 |  |  | 0.867 | 0.854 | 0.740 |  |  |  |
|  |  | SM_21 |  |  |  | 0.823 | 0.733 |  |  |  |
|  |  | SM_22 |  |  |  |  | 0.711 |  |  |  |
|  | AUDPC |  | SM_23 |  |  |  |  |  |  |  |
|  |  | BE_23 | 0.688 |  |  |  |  |  |  | 0.688 |
| Apricot rust leaf fall | Max |  | BE_23 | SM_20 | SM_21 | SM_23 |  |  |  |  |
|  |  | BE_22 | 0.732 | 0.677 | 0.646 | 0.682 |  |  |  | 0.720 |
|  |  | BE_23 |  | 0.626 | 0.634 | 0.692 |  |  |  |  |
|  |  | SM_20 |  |  | 0.883 | 0.788 |  |  |  |  |
|  |  | SM_21 |  |  |  | 0.836 |  |  |  |  |
|  | AUDPC |  | SM_23 |  |  |  |  |  |  |  |
|  |  | BE_23 | 0.781 |  |  |  |  |  |  | 0.781 |

**A**

**B**als

**C**als

**D**als

**E**als

**F**als


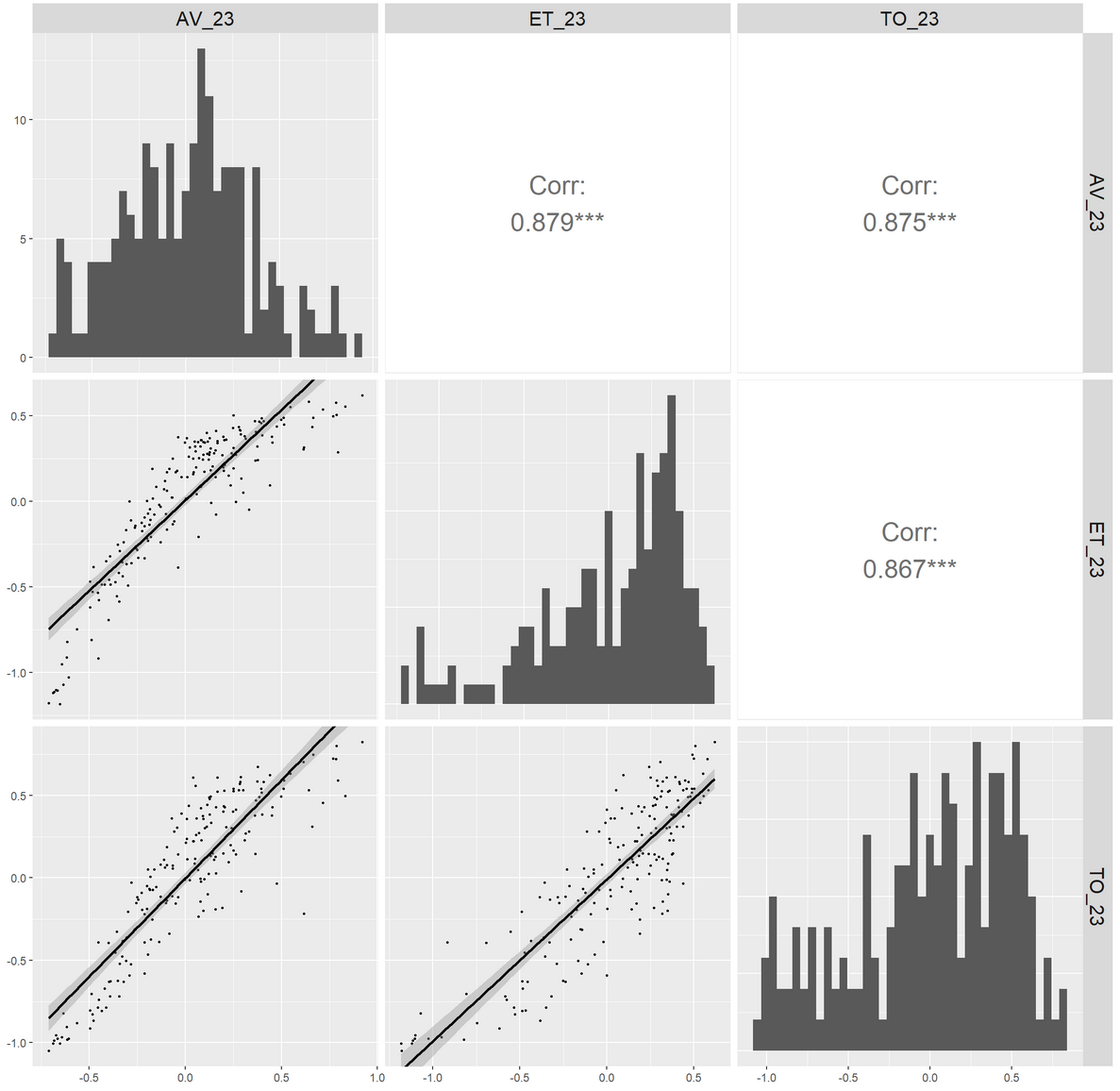

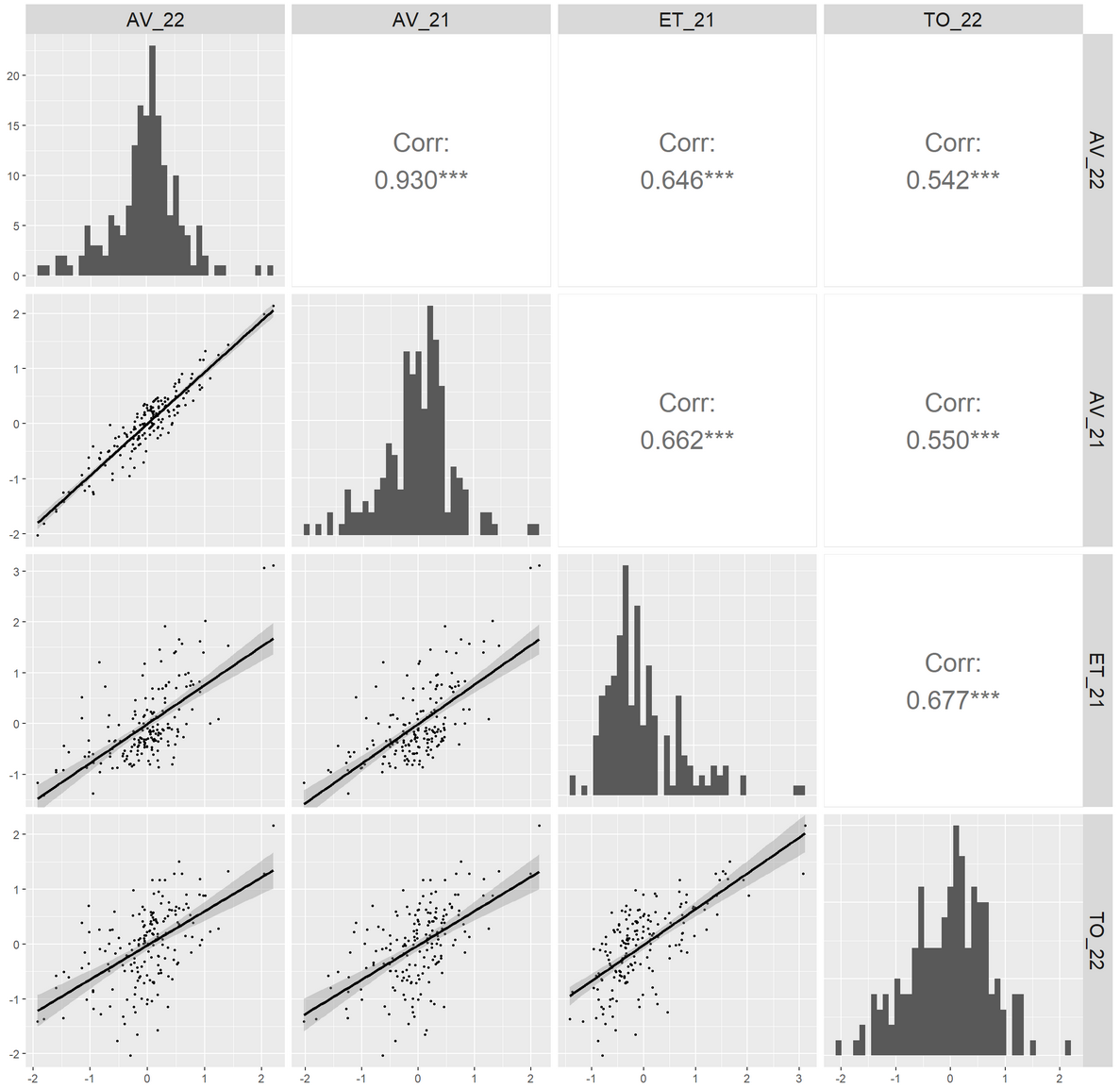

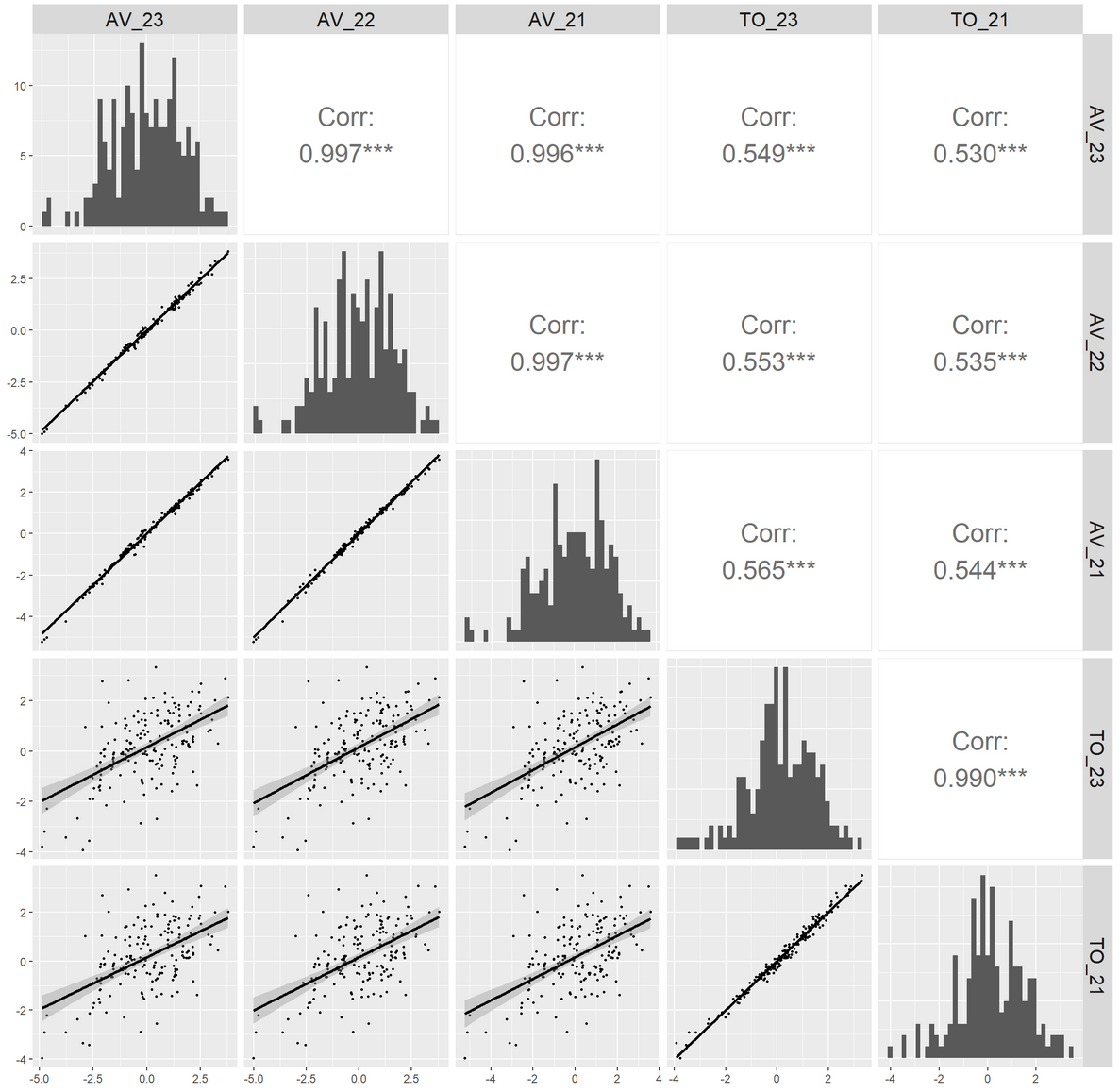

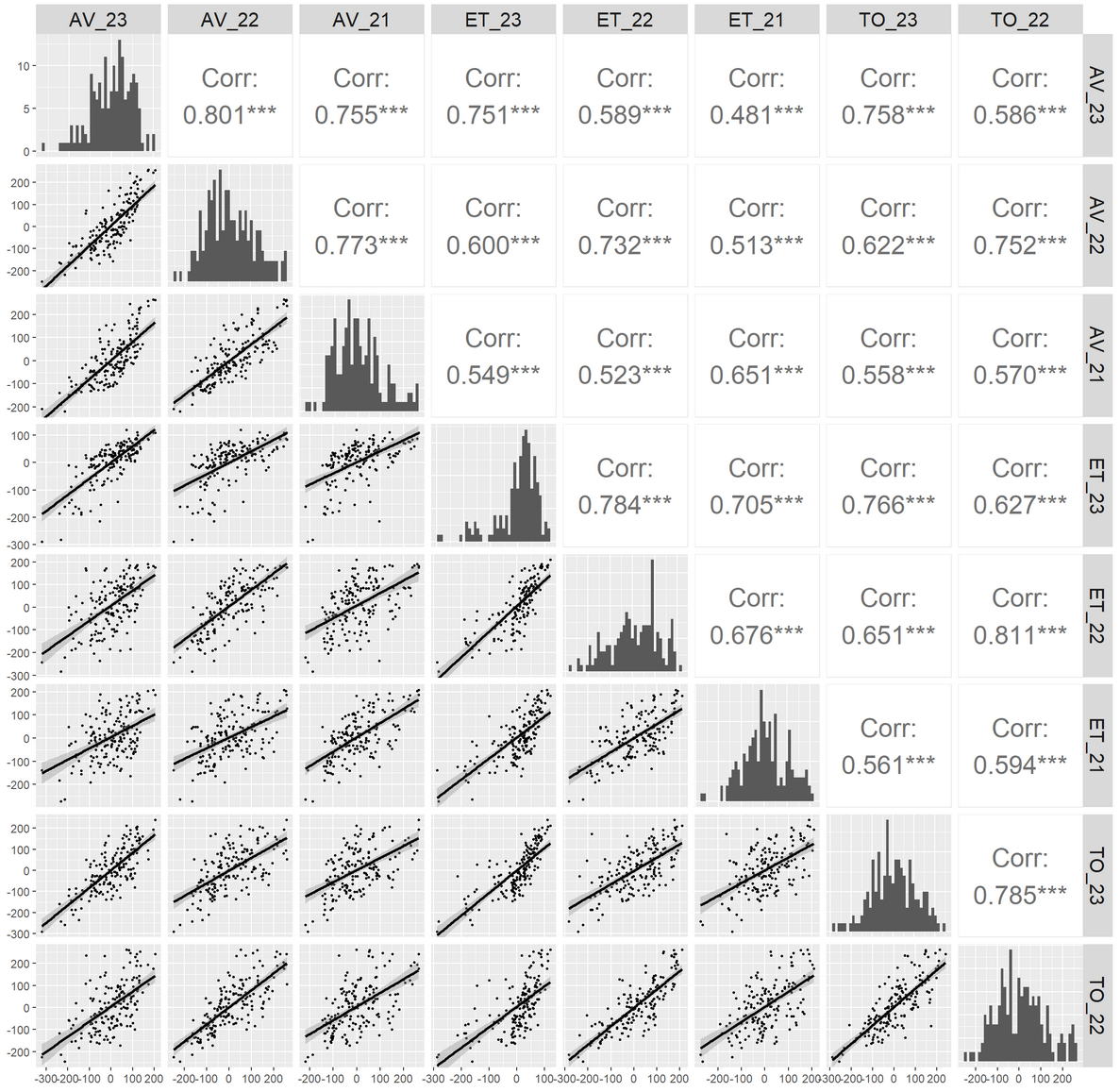

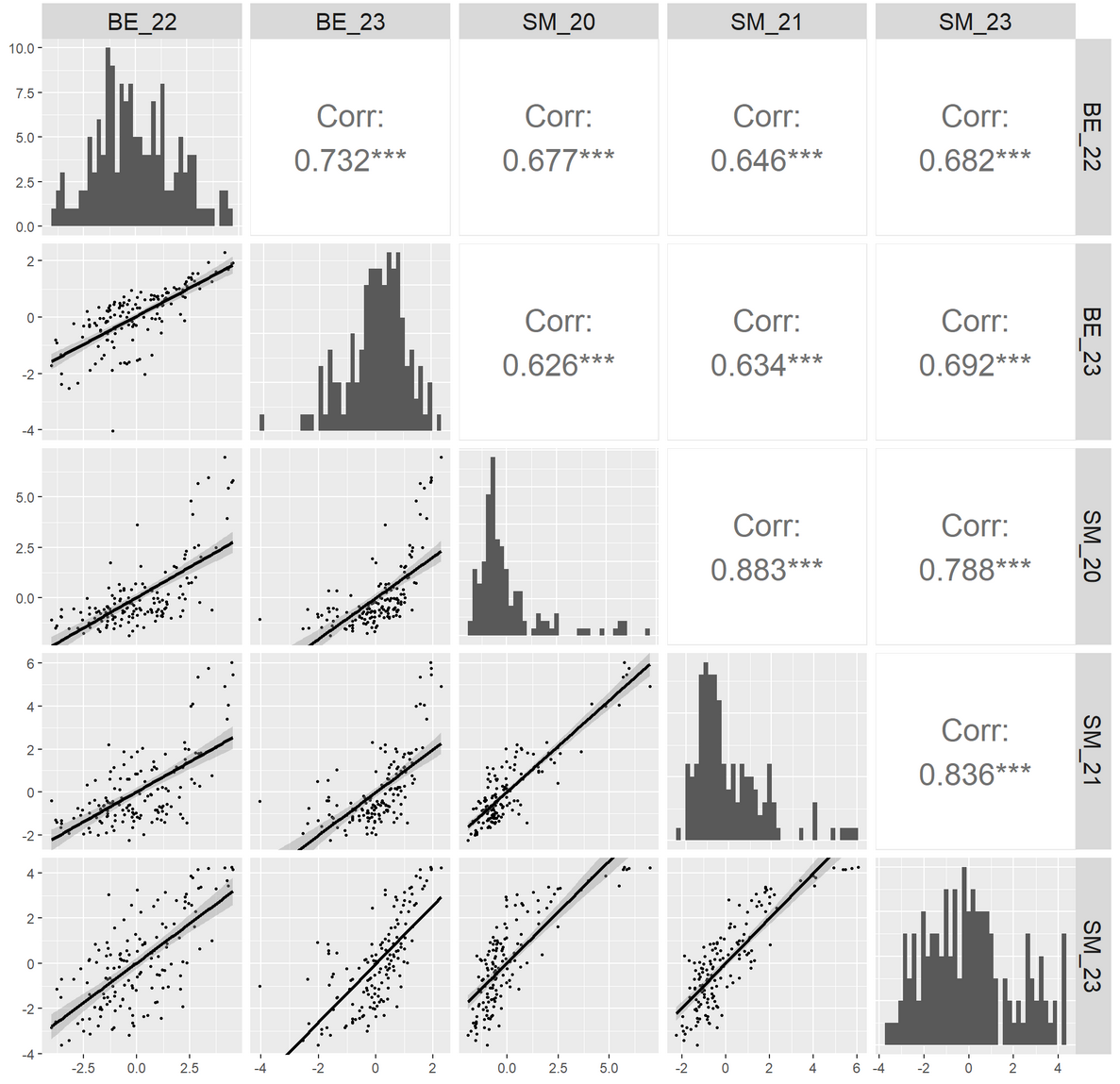

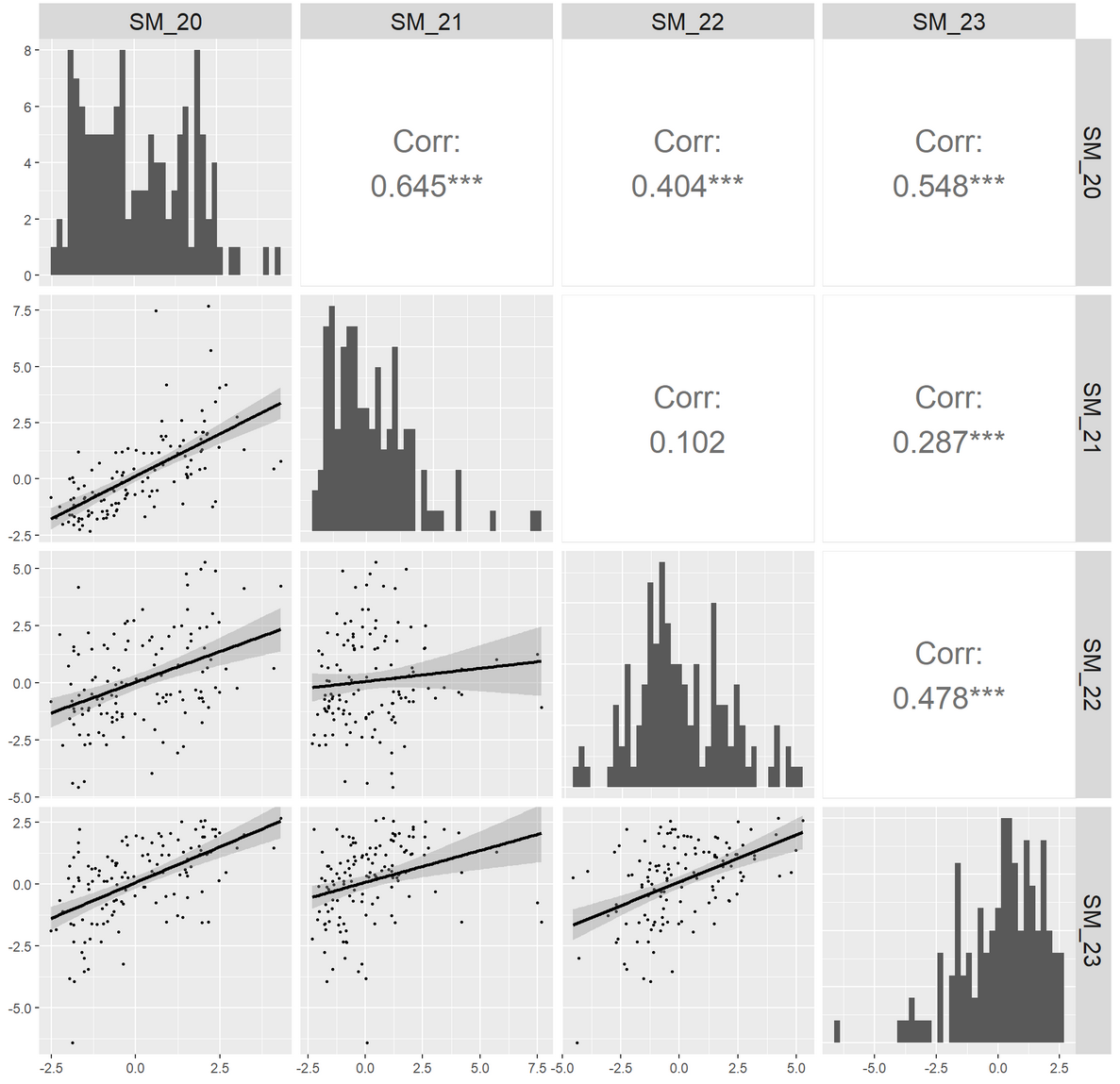

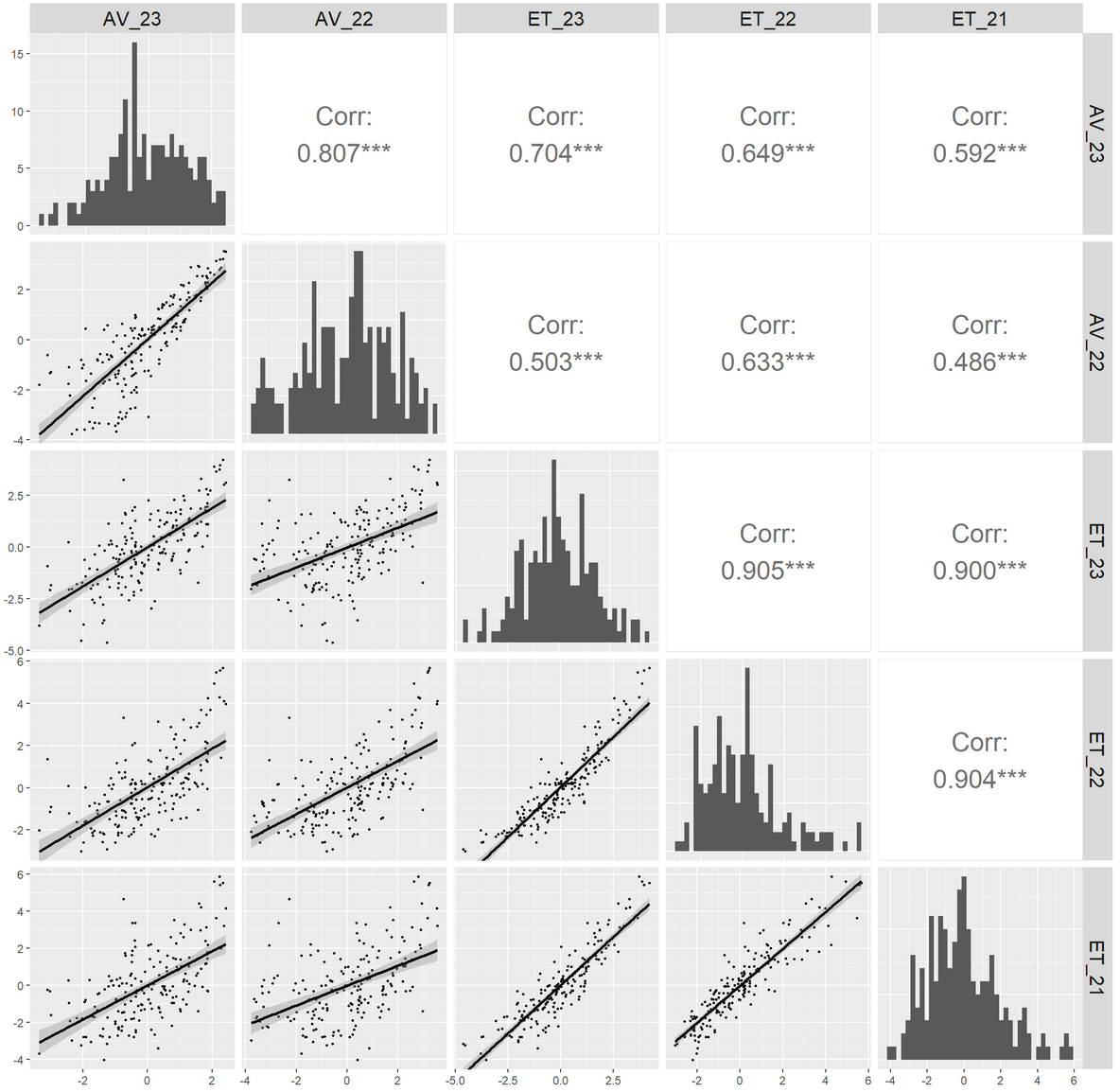

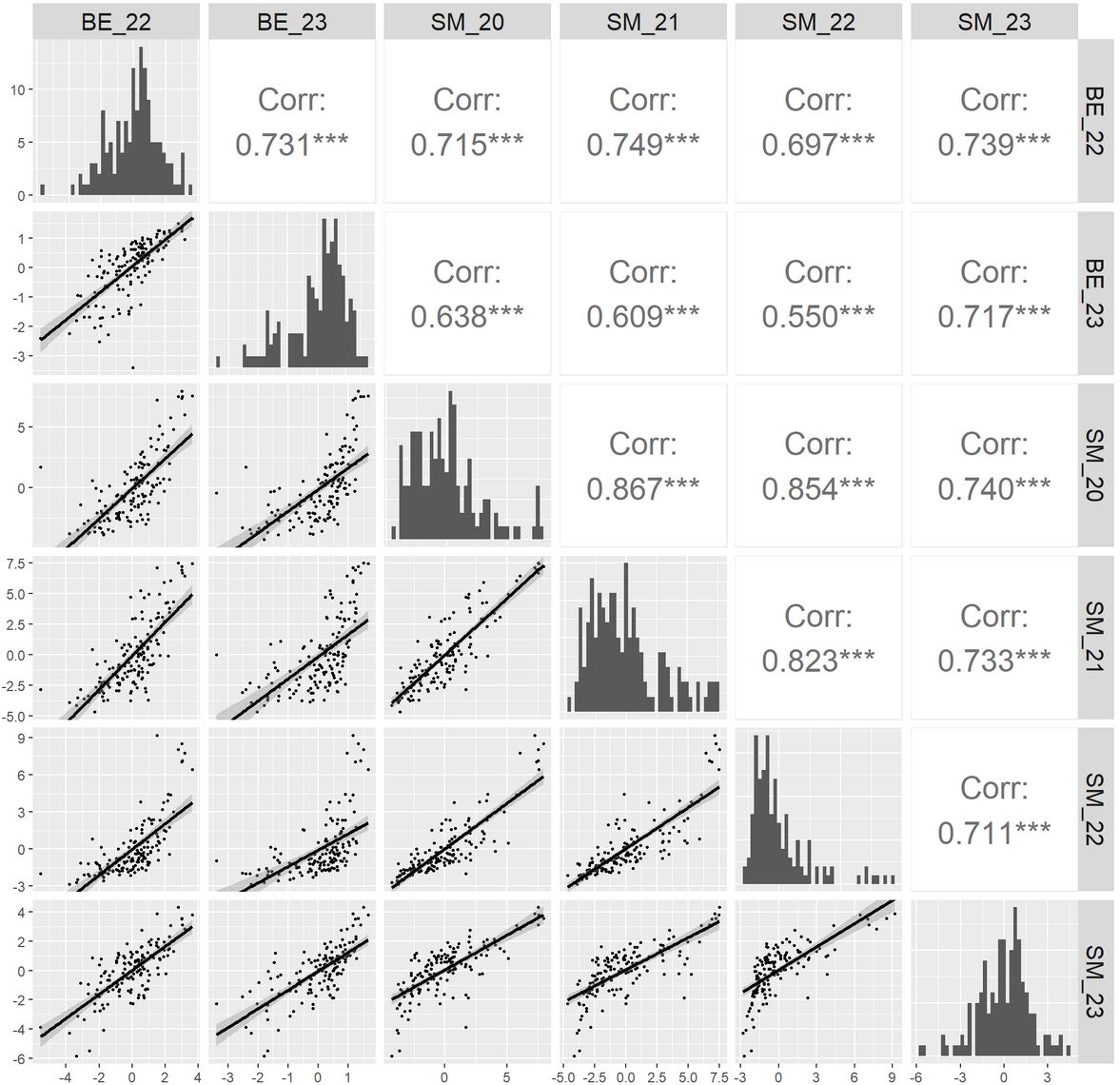


**G**als

**H**als

**Supplementary Fig 4.** Distribution of the environment-specific BLUPs of Max in each environment (in the diagonal) and correlations between the environment-specific BLUPs (values and significance in cells above the diagonal and scatter plots in cells below) for A) leaf curl B) leafhopper C) powdery mildew D) Peach Rust E) shot hole F) blossom blight G) Apricot rust and H) apricot rust leaf fall.


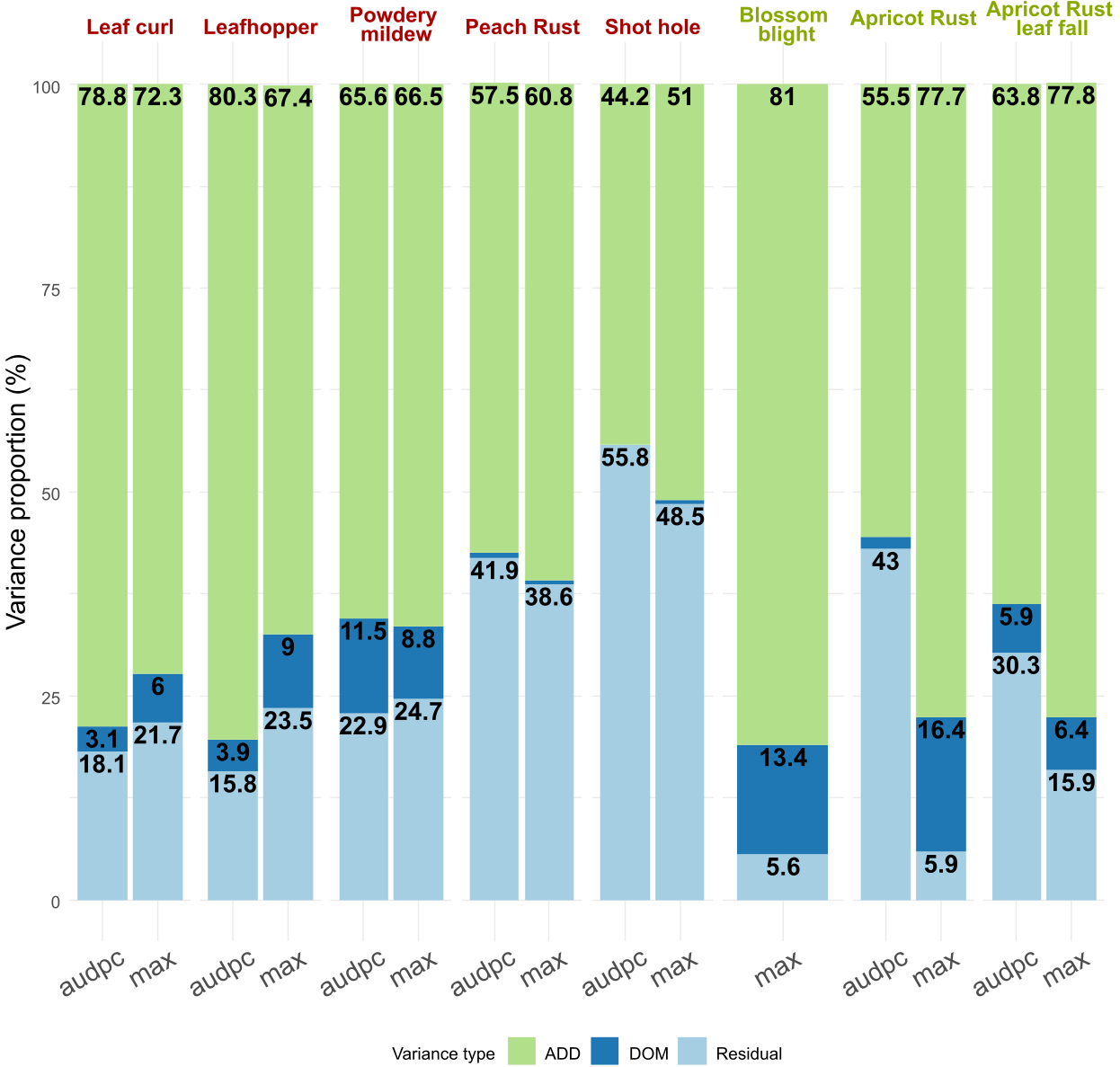


**Supplementary Fig 5.** Variance decomposition across environments for the different pests and diseases using dominant models.

**Supplementary Fig 6.** Linkage disequilibrium (LD) estimated per chromosome through the measurement of the squared correlation coefficients (r^2^) for A) the peach core collection calculated with 13,680 markers and B) the apricot core collection calculated with 41,145 SNPs markers after pruning.

The red dotted lines in both plots refer to the 0.2 threshold. For a better visualization, SNPs have been grouped by distance blocks of 2000 bp for peach and 5 bp for apricot.

**A**als

**B**als


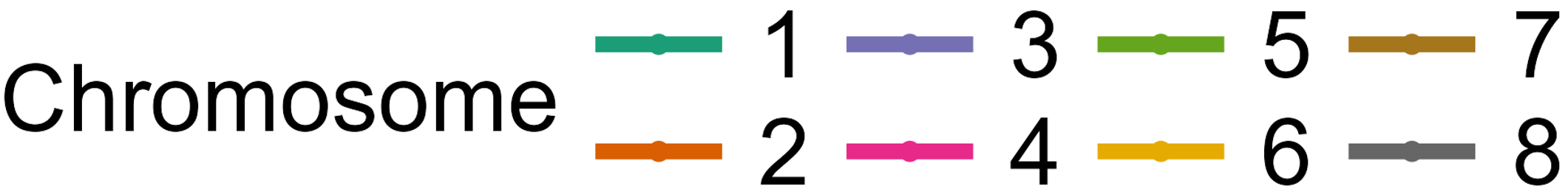

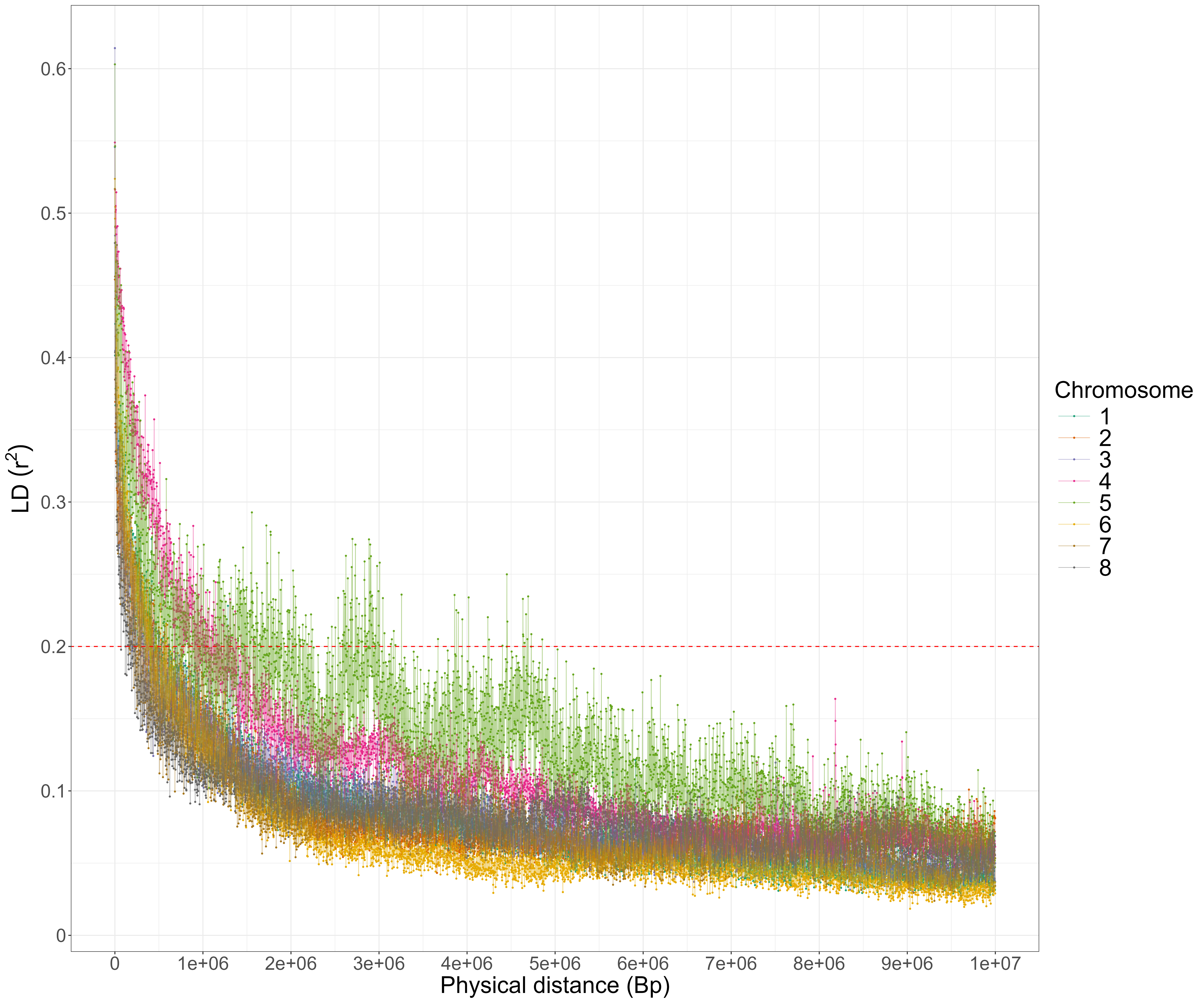

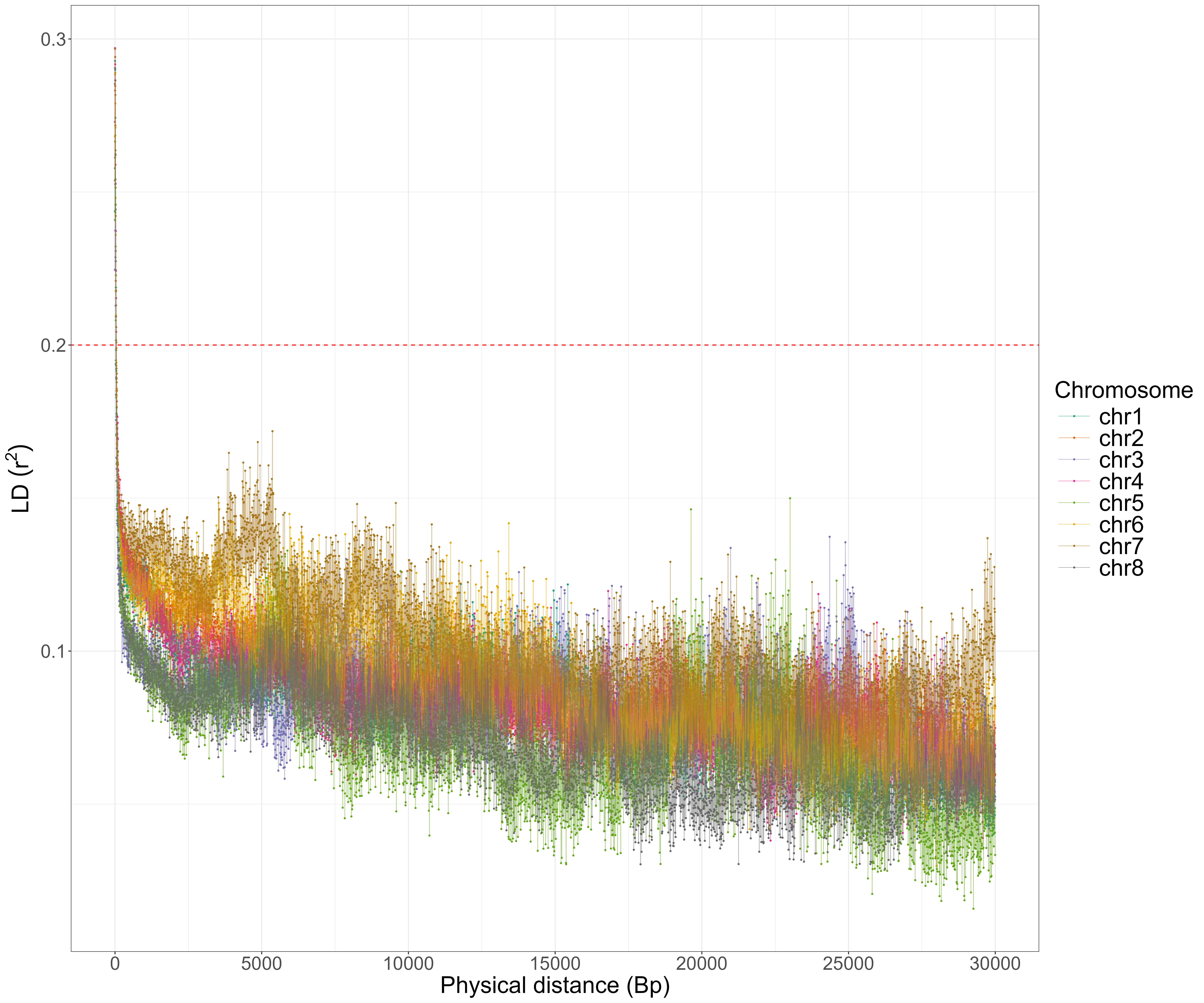


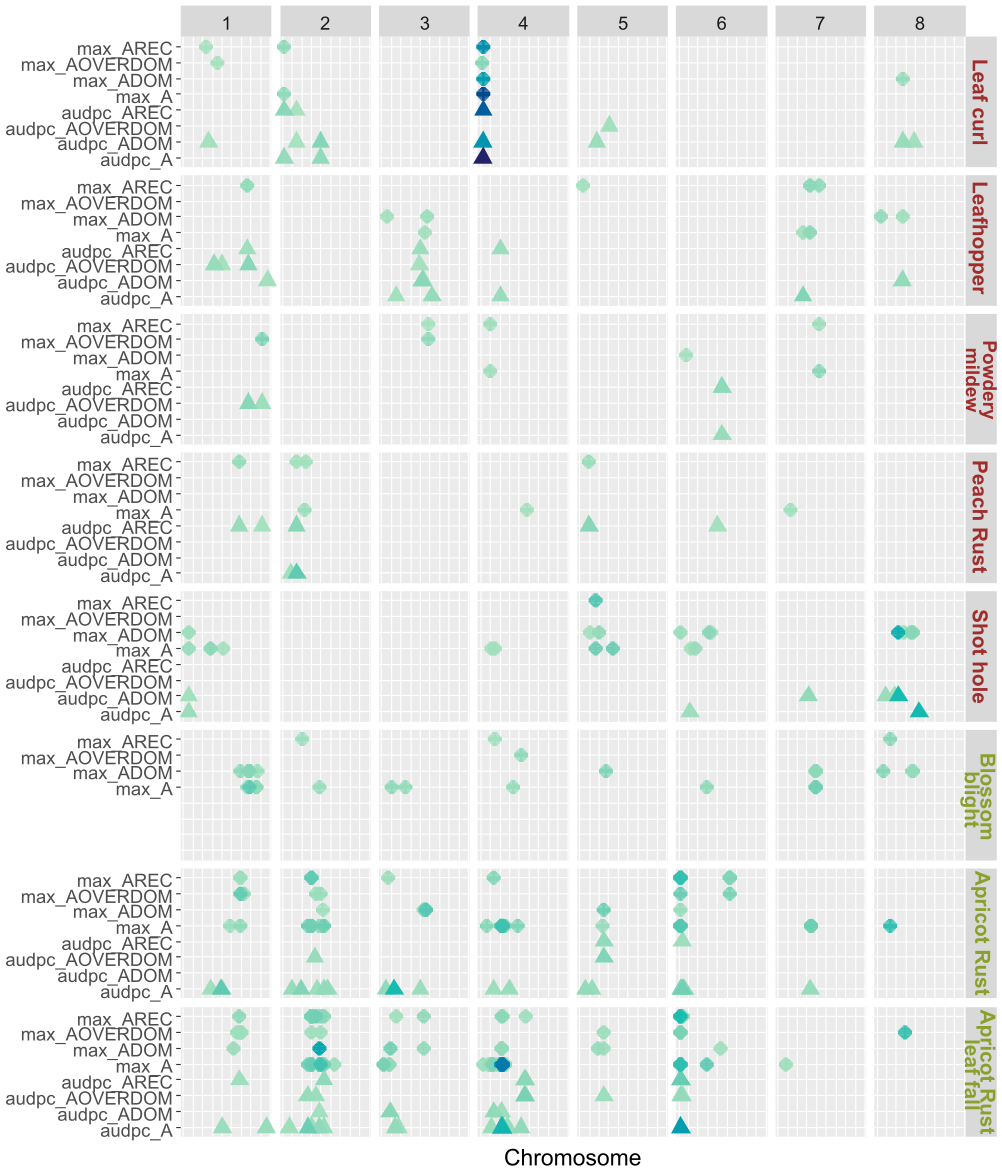


**Supplementary Fig 7.** QTL summary showing the physical position of GWAS significant SNPs. Each horizontal line contains QTLs of one biotic stress, organized by recessive, overdominance, dominance and analysis (called AREC, AVOERDEM, ADOM and A) and either for the maximum of damage score (called max) or the AUDPC. The dot colours are proportional to the -log_10_(P-value) of the QTL top SNP.

**A**


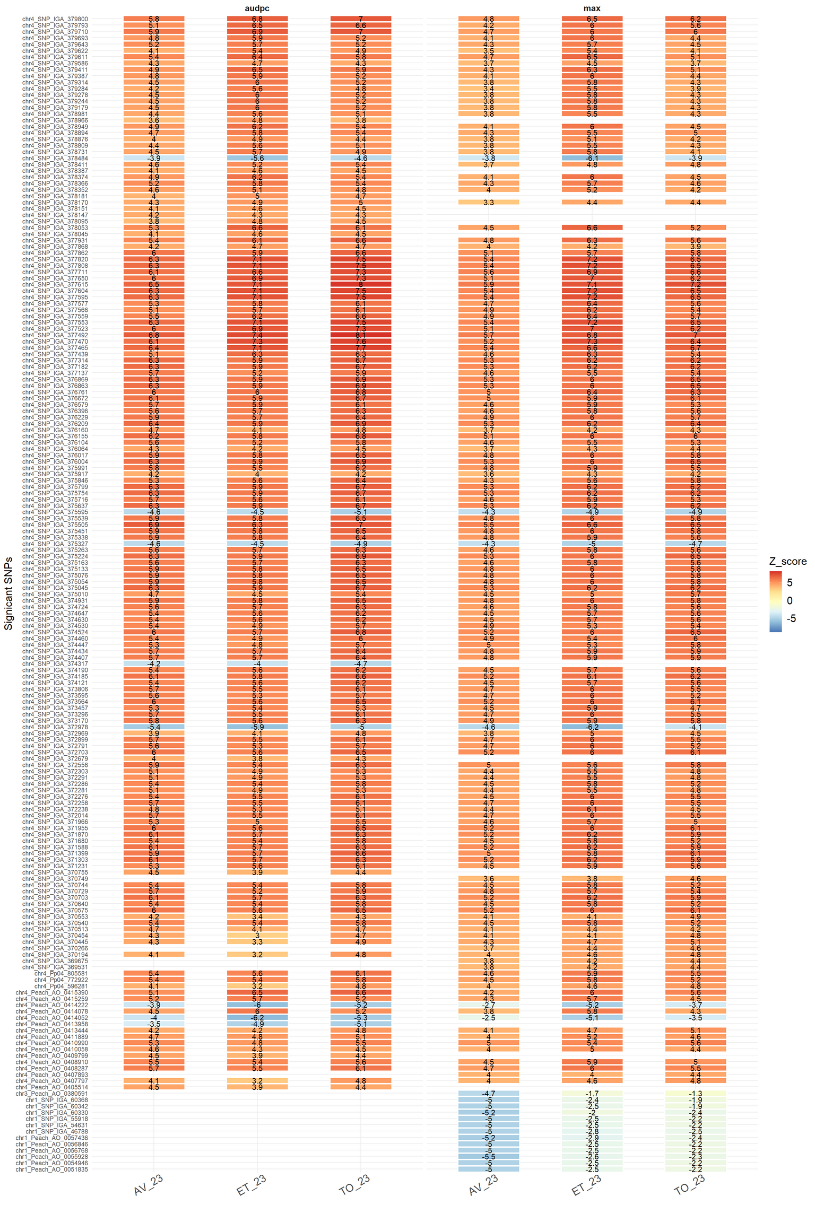


**B**

**C**

**D**

**E**


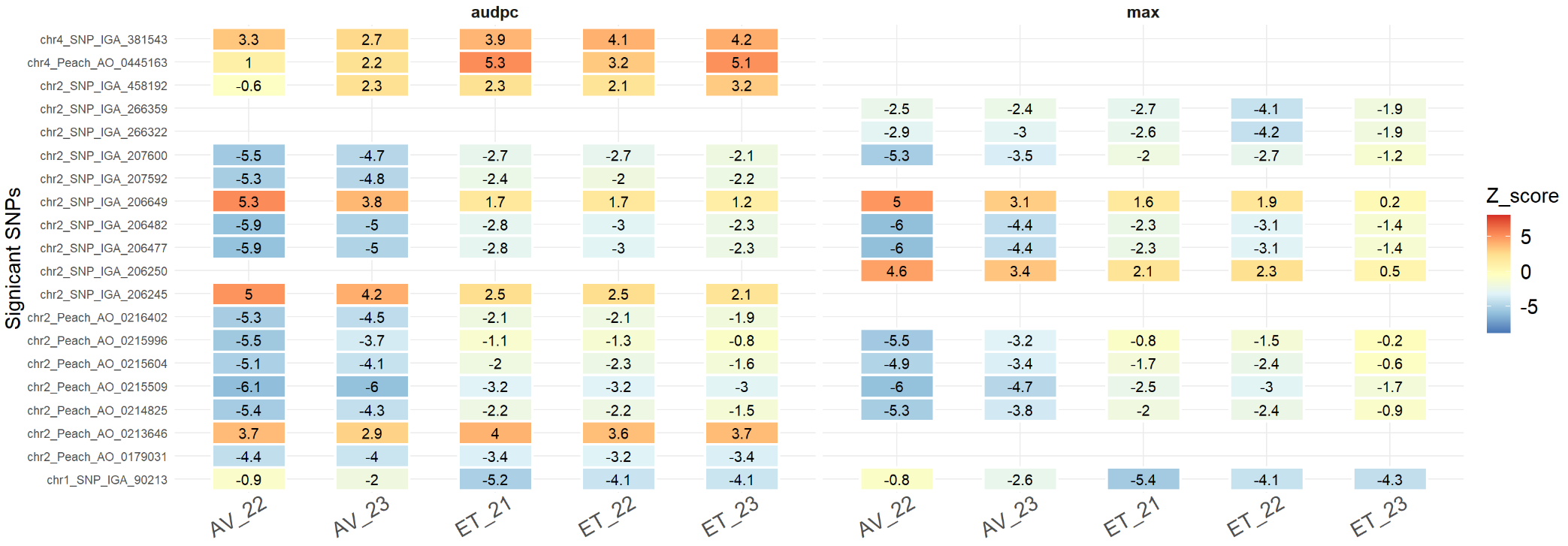

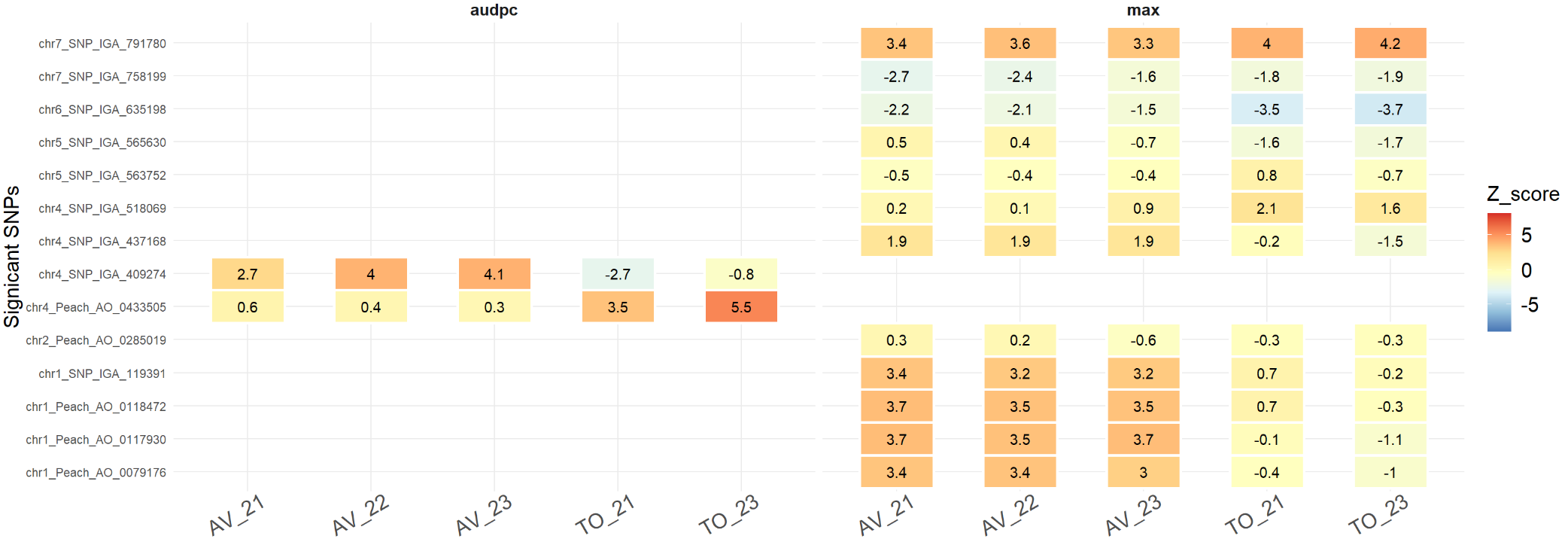

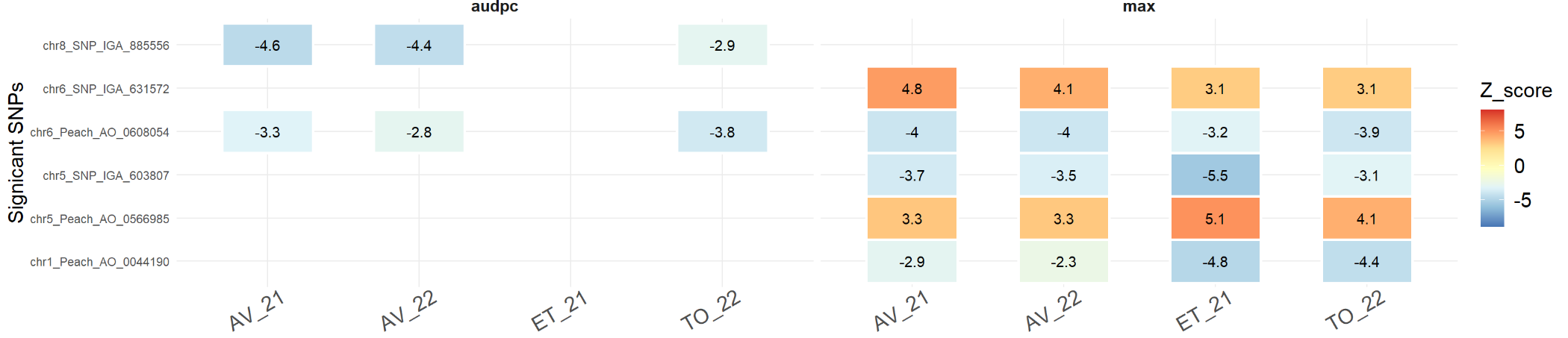

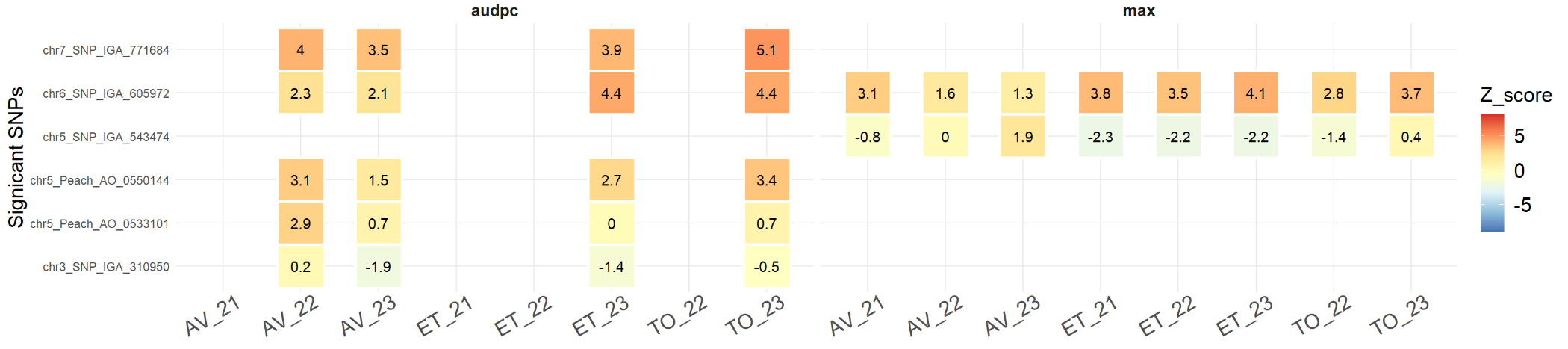


**F**

**G**


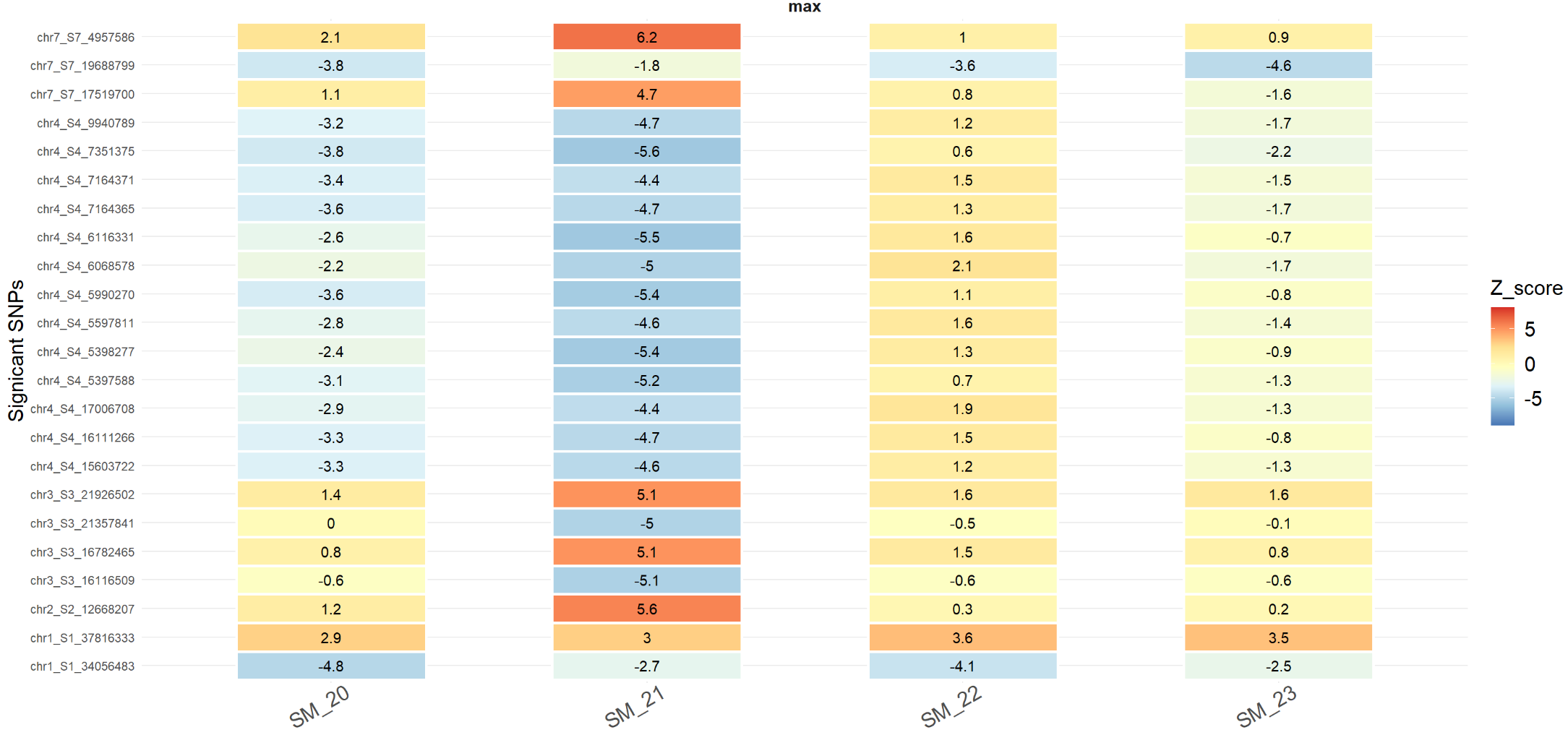

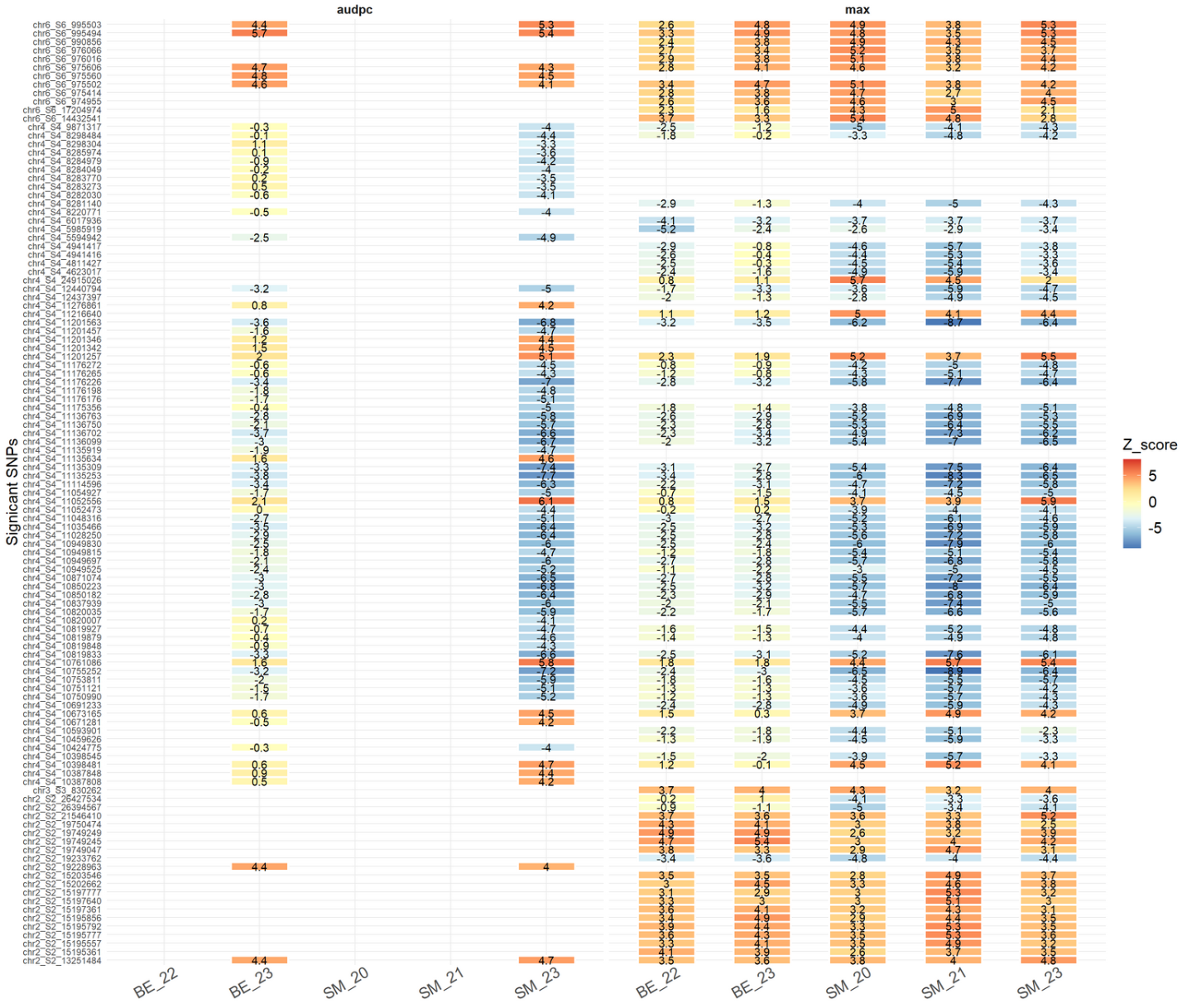


**H**


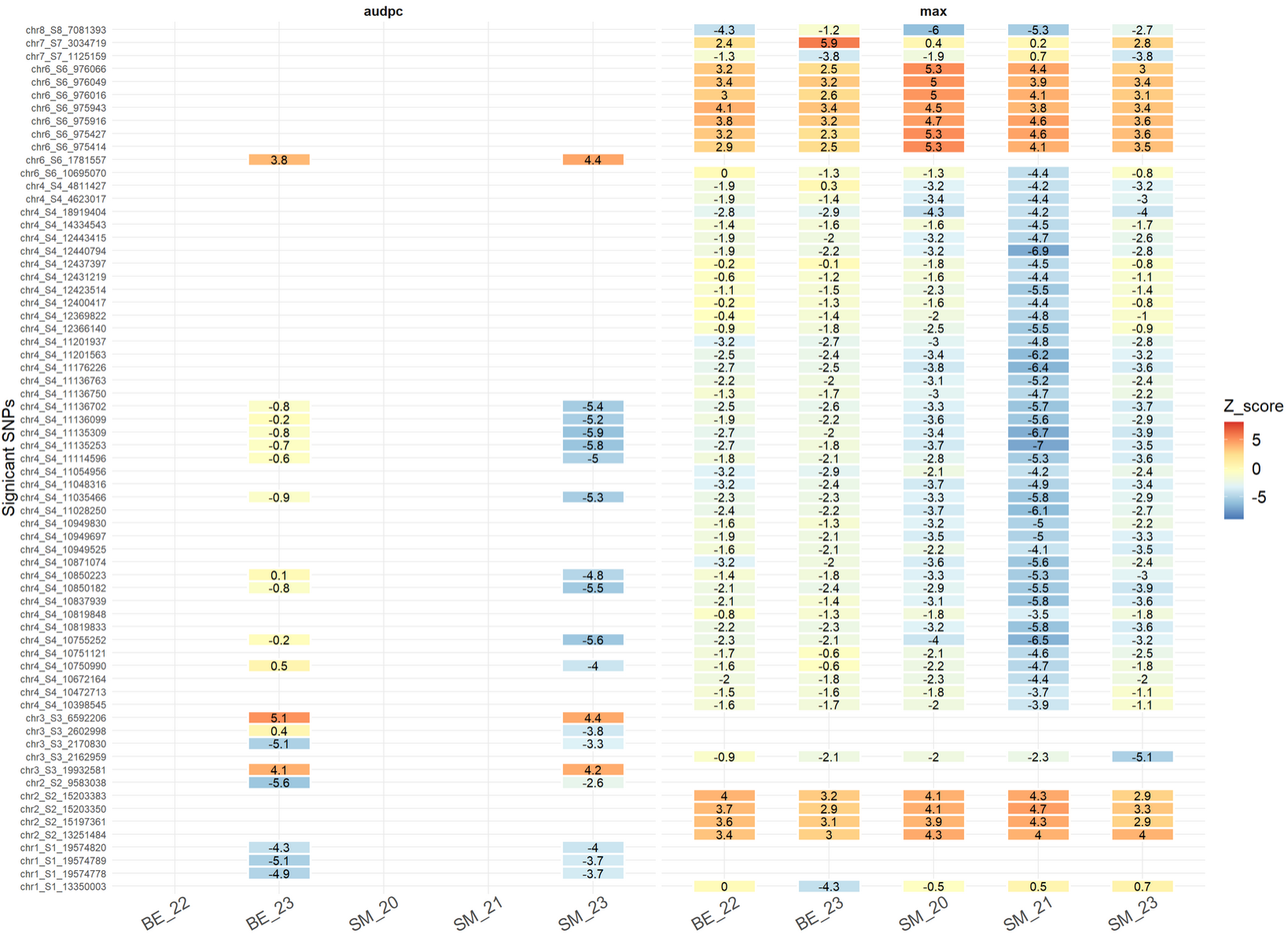


**Supplementary Fig 8**. Heatmap of z-scores of significant SNPs detected with Meta-GWAS for A) leaf curl B) leafhopper C) powdery mildew D) peach rust E) shot hole F) blossom blight G) apricot rust and HG) apricot rust leaf fall. Markers are displayed in rows and environments in columns.

**Supplementary Table 2.** Summary of the high confidence QTLs identified

| Biotic stress | QTL Name | Trait | Detected model | Top SNP | Chr | Pos | -log_10_  (*P*-value) |
| --- | --- | --- | --- | --- | --- | --- | --- |
| Leaf curl | Leaf_curl_4 | AUDPC and Max | Across-environment additive, Single environment AV_2023, ET_2023, TO_2023, Meta-GWAS | SNP_IGA_377492 | 4 | 12553991 | 15.2 |
| Leafhopper | Leafhopper_6 | AUDPC | Single environment ET_2022, ET_2023, TO_2023, Meta-GWAS | SNP_IGA_605972 | 6 | 4410256 | 5.2 |
|  | Leafhopper_7_1 | AUDPC | Single environment ET_2022, ET_2023, AV_2022, | Peach_AO_0740041 | 7 | 10519835 | 5.2 |
|  | Leafhopper_7_2 | AUDPC | Across-environment additive, Single environment TO_2023, Meta-GWAS | SNP_IGA_771684 | 7 | 12938823 | 6.4 |
| Powdery mildew | Powdery_mildew_6 | Max | Single environment AV_2021, AV_2022, AV_2023, Meta-GWAS | SNP_IGA_637345 | 6 | 10618803 | 5.4 |
| Peach Rust | Peach_Rust_2 | AUDPC | Across-environment additive, Single environment AV_2022, AV_2023, Meta-GWAS | Peach_AO_0215509 | 2 | 7459174 | 8.9 |
|  | Peach_Rust_4 | AUDPC | Single environment ET_2021, ET_2022, ET_2023, Meta-GWAS | Peach_AO_0419003 | 4 | 2958070 | 5.2 |
| Shot hole | Shot_hole_6 | Max | Across-environment additive, Single environment AV_2021, AV_2022, Meta-GWAS, MTMM_common | SNP_IGA_631572 | 6 | 8704993 | 5.9 |
|  | Shot_hole_8 | AUDPC | Across-environment additive, Single environment AV_2021, AV_2022, Meta-GWAS | SNP_IGA_885556 | 8 | 21801227 | 7.5 |
| Apricot Rust | Apricot_Rust_1 | AUDPC | Across-environment additive, Single environment BE_2023, Meta-GWAS | S1_19574789 | 1 | 19574789 | 6.5 |
|  | Apricot_Rust_2 | AUDPC | Across-environment additive, Single environment BE_2023, Meta-GWAS | S2_9583038 | 2 | 9583038 | 7.8 |
|  | Apricot_Rust_3.1 | AUDPC | Across-environment additive, Single environment BE_2023, Meta-GWAS | S3_2170830 | 3 | 2170830 | 6.4 |
|  | Apricot_Rust_3.2 | AUDPC | Across-environment additive, Single environment BE_2023, GO_2023, Meta-GWAS | S3_6592206 | 3 | 6592206 | 7.4 |
|  | Apricot_Rust_3.3 | AUDPC | Across-environment additive, Single environment GO_2023, Meta-GWAS | S3_19932581 | 3 | 19932581 | 4.9 |
|  | Apricot_Rust_4.1 | AUDPC and Max | Across-environment additive, Single environment SM_2021, SM_2022, Meta-GWAS, MTMM_full, MTMM_common | S4_10755252 | 4 | 10755252 | 14.5 |
|  | Apricot_Rust_4.2 | AUDPC | Single environment SM_2023, Meta-GWAS, MTMM_full, MTMM_common | S4_10850182 | 4 | 10850182 | 7.5 |
|  | Apricot_Rust_4.3 | AUDPC | Single environment SM_2023, Meta-GWAS, MTMM_full, MTMM_common | S4_11114596 | 4 | 11114596 | 6.3 |
|  | Apricot_Rust_4.4 | Max | Across-environment additive, Single environment SM_2021, SM_2022, Meta-GWAS, MTMM_full, MTMM_common | S4_11135253 | 4 | 11135253 | 11.6 |
|  | Apricot_Rust_4.5 | AUDPC | Single environment SM_2023, Meta-GWAS, MTMM_full, MTMM_common | S4_11135309 | 4 | 11135309 | 8.8 |
|  | Apricot_Rust_4.6 | AUDPC | Single environment SM_2023, Meta-GWAS, MTMM_full, MTMM_common | S4_11136099 | 4 | 11136099 | 7.6 |
|  | Apricot_Rust_4.7 | AUDPC | Single environment SM_2023, Meta-GWAS, MTMM_full, MTMM_common | S4_11136702 | 4 | 11136702 | 7.1 |
|  | Apricot_Rust_4.8 | Max | Across-environment additive, Single environment SM_2021,SM_2021, SM_2022, Meta-GWAS, MTMM_common | S4_11176226 | 4 | 11176226 | 10.6 |
|  | Apricot_Rust_6.1 | Max | Across-environment additive, Single environment SM_2020, SM_2021, SM_2022, Meta-GWAS,  MTMM_common | S6_975427 | 6 | 975427 | 7.0 |
|  | Apricot_Rust_6.2 | Max | Across-environment additive, Single environment SM_2020, SM_2021, SM_2022, Meta-GWAS,  MTMM_common | S6_976066 | 6 | 976066 | 6.8 |
|  | Apricot_Rust_8 | Max | Across-environment additive, Single environment SM_2020, SM_2021, SM_2022, BE_2022, Meta-GWAS, MTMM_full,  MTMM_common | S8_7081393 | 8 | 7081393 | 8.7 |
| Apricot Rust Leaf fall | Apricot_Rust_leaf_fall_2.1 | AUDPC and Max | Across-environment additive, Single environment SM_2023, BE_2023, Meta-GWAS, MTMM_full,  MTMM_common | S2_13251484 | 2 | 13251484 | 6 |
|  | Apricot_Rust_leaf_fall_2.2 | Max | Across-environment additive and non-additive, Single environment SM_2021, BE_2023, Meta-GWAS,  MTMM_common | S2_15195792 | 2 | 15195792 | 6.9 |
|  | Apricot_Rust_leaf_fall_2.3 | Max | Across-environment additive, Single environment SM_2020, SM_2023, Meta-GWAS,  MTMM_common | S2_19233762 | 2 | 19233762 | 6.0 |
|  | Apricot_Rust_leaf_fall_2.4 | Max | Across-environment additive, Single environment SM_2021, SM_2023, BE_2022, Meta-GWAS, | S2_19749245 | 2 | 19749245 | 7.3 |
|  | Apricot_Rust_leaf_fall_2.5 | Max | Across-environment additive, Single environment BE_2023, Meta-GWAS, MTMM_full,  MTMM_common | S2_19750367 | 2 | 19750367 | 4.9 |
|  | Apricot_Rust_leaf_fall_3 | Max | Across-environment additive, Single environment SM_2020, SM_2021, Meta-GWAS,  MTMM_common | S3_830262 | 3 | 830262 | 5.8 |
|  | Apricot_Rust_leaf_fall_4.1 | AUDPC | Single environment SM_2023, Meta-GWAS, MTMM_full, MTMM_gei | S4_8298484 | 4 | 8298484 | 6.8 |
|  | Apricot_Rust_leaf_fall_4.2 | Max | Across-environment additive and non-additive, Single environment SM_2020, SM_2021, SM_2023, Meta-GWAS,  MTMM_common | S4_10691233 | 4 | 10691233 | 8.4 |
|  | Apricot_Rust_leaf_fall_4.3 | Max and AUDPC | Across-environment additive, Single environment SM_2020, SM_2021, SM_2023, Meta-GWAS, MTMM_full | S4_10753811 | 4 | 10753811 | 8.5 |
|  | Apricot_Rust_leaf_fall_4.4 | Max and AUDPC | Across-environment additive, Single environment SM_2020, SM_2021, SM_2023, Meta-GWAS, MTMM_full, MTMM_common | S4_10755252 | 4 | 10755252 | 18.2 |
|  | Apricot_Rust_leaf_fall_4.5 | Max | Across-environment additive and non-additive, Single environment SM_2020, SM_2021, SM_2023, Meta-GWAS,  MTMM_full | S4_10761086 | 4 | 10761086 | 8 |
|  | Apricot_Rust_leaf_fall_4.6 | Max and AUDPC | Across-environment additive and non-additive, Single environment SM_2020, SM_2021, SM_2023, Meta-GWAS,  MTMM_full, MTMM_common | S4_10819833 | 4 | 10819833 | 13.6 |
|  | Apricot_Rust_leaf_fall_4.7 | Max and AUDPC | Across-environment additive, Single environment SM_2020, SM_2021, SM_2023, Meta-GWAS,  MTMM_full, MTMM_common | S4_10837939 | 4 | 10837939 | 12.8 |
|  | Apricot_Rust_leaf_fall_4.8 | Max and AUDPC | Across-environment additive, Single environment SM_2020, SM_2021, SM_2023, Meta-GWAS,  MTMM_full, MTMM_common | S4_10850223 | 4 | 10850223 | 14.8 |
|  | Apricot_Rust_leaf_fall_4.9 | Max and AUDPC | Across-environment additive, Single environment SM_2020, SM_2021, SM_2023, Meta-GWAS, MTMM_common | S4_10871074 | 4 | 10871074 | 12.2 |
|  | Apricot_Rust_leaf_fall_4.10 | Max and AUDPC | Across-environment additive, Single environment SM_2020, SM_2021, SM_2023, Meta-GWAS, MTMM_full, MTMM_common, MTMM_gei | S4_10949830 | 4 | 10949830 | 14.4 |
|  | Apricot_Rust_leaf_fall_4.11 | Max and AUDPC | Across-environment additive, Single environment SM_2020, SM_2021, SM_2023, Meta-GWAS, MTMM_full, MTMM_common | S4_11028250 | 4 | 11028250 | 12.1 |
|  | Apricot_Rust_leaf_fall_4.12 | Max and AUDPC | Across-environment additive, Single environment SM_2020, SM_2021, SM_2023, Meta-GWAS, MTMM_full, MTMM_common | S4_11035466 | 4 | 11035466 | 11.4 |
|  | Apricot_Rust_leaf_fall_4.13 | Max | Across-environment additive, Single environment SM_2020, SM_2021, SM_2023, Meta-GWAS, MTMM_common | S4_11048316 | 4 | 11048316 | 8.8 |
|  | Apricot_Rust_leaf_fall_4.14 | AUDPC | Across-environment additive, Single environment SM_2023, Meta-GWAS, MTMM_full, MTMM_gei | S4_11052556 | 4 | 11052556 | 9.1 |
|  | Apricot_Rust_leaf_fall_4.15 | Max and AUDPC | Across-environment additive, Single environment SM_2020, SM_2021, SM_2023, Meta-GWAS, MTMM_full, MTMM_common | S4_11114596 | 4 | 11114596 | 12.2 |
|  | Apricot_Rust_leaf_fall_4.16 | Max and AUDPC | Across-environment additive, Single environment SM_2020, SM_2021, SM_2023, Meta-GWAS, MTMM_full, MTMM_common | S4_11135253 | 4 | 11135253 | 16 |
|  | Apricot_Rust_leaf_fall_4.17 | Max and AUDPC | Across-environment additive, Single environment SM_2020, SM_2021, SM_2023, Meta-GWAS, MTMM_full, MTMM_common | S4_11136099 | 4 | 11136099 | 11.6 |
|  | Apricot_Rust_leaf_fall_4.18 | Max and AUDPC | Across-environment additive, Single environment SM_2020, SM_2021, SM_2023, Meta-GWAS, MTMM_full, MTMM_common | S4_11136702 | 4 | 11136702 | 12.4 |
|  | Apricot_Rust_leaf_fall_4.19 | AUDPC | Single environment SM_2023, Meta-GWAS, MTMM_full, MTMM_gei | S4_11175356 | 4 | 11175356 | 8.4 |
|  | Apricot_Rust_leaf_fall_4.20 | Max and AUDPC | Across-environment additive, Single environment SM_2020, SM_2021, SM_2023, Meta-GWAS, MTMM_full, MTMM_common | S4_11176226 | 4 | 11176226 | 13.9 |
|  | Apricot_Rust_leaf_fall_4.21 | Max | Across-environment additive, Single environment SM_2020, SM_2021, SM_2023, Meta-GWAS, MTMM_full | S4_11201257 | 4 | 11201257 | 7.5 |
|  | Apricot_Rust_leaf_fall_4.22 | Max and AUDPC | Across-environment additive, Single environment SM_2020, SM_2021, SM_2023, Meta-GWAS, MTMM_full, MTMM_common | S4_11201563 | 4 | 11201563 | 17.5 |
|  | Apricot_Rust_leaf_fall_4.23 | Max | Across-environment additive, Single environment SM_2021, SM_2023, Meta-GWAS, MTMM_full | S4_12440794 | 4 | 12440794 | 8.5 |
|  | Apricot_Rust_leaf_fall_6.1 | Max | Across-environment additive, Single environment SM_2020, SM_2023, Meta-GWAS, MTMM_full | S6_974955 | 6 | S6_974955 | 5.3 |
|  | Apricot_Rust_leaf_fall_6.2 | Max and AUDPC | Across-environment additive, Single environment SM_2020, SM_2023, BE_2023, Meta-GWAS, MTMM_common | S6_975502 | 6 | 975502 | 6.7 |
|  | Apricot_Rust_leaf_fall_6.3 | Max | Across-environment additive, Single environment SM_2020, SM_2023, BE_2023, Meta-GWAS, MTMM_common | S6_976066 | 6 | 976066 | 6.6 |
|  | Apricot_Rust_leaf_fall_6.4 | Max | Across-environment additive and non-additive, Single environment SM_2020, SM_2021, BE_2023, Meta-GWAS, MTMM_common | S6_990856 | 6 | 990856 | 7.2 |
|  | Apricot_Rust_leaf_fall_6.5 | Max and AUDPC | Across-environment additive and non-additive, Single environment SM_2020, SM_2023, BE_2023, Meta-GWAS, MTMM_full, MTMM_common | S6_995494 | 6 | 995494 | 9.2 |
|  | Apricot_Rust_leaf_fall_6.6 | Max | Across-environment additive, Single environment SM_2020, SM_2021, Meta-GWAS, MTMM_common | S6_14432541 | 6 | 14432541 | 7.2 |


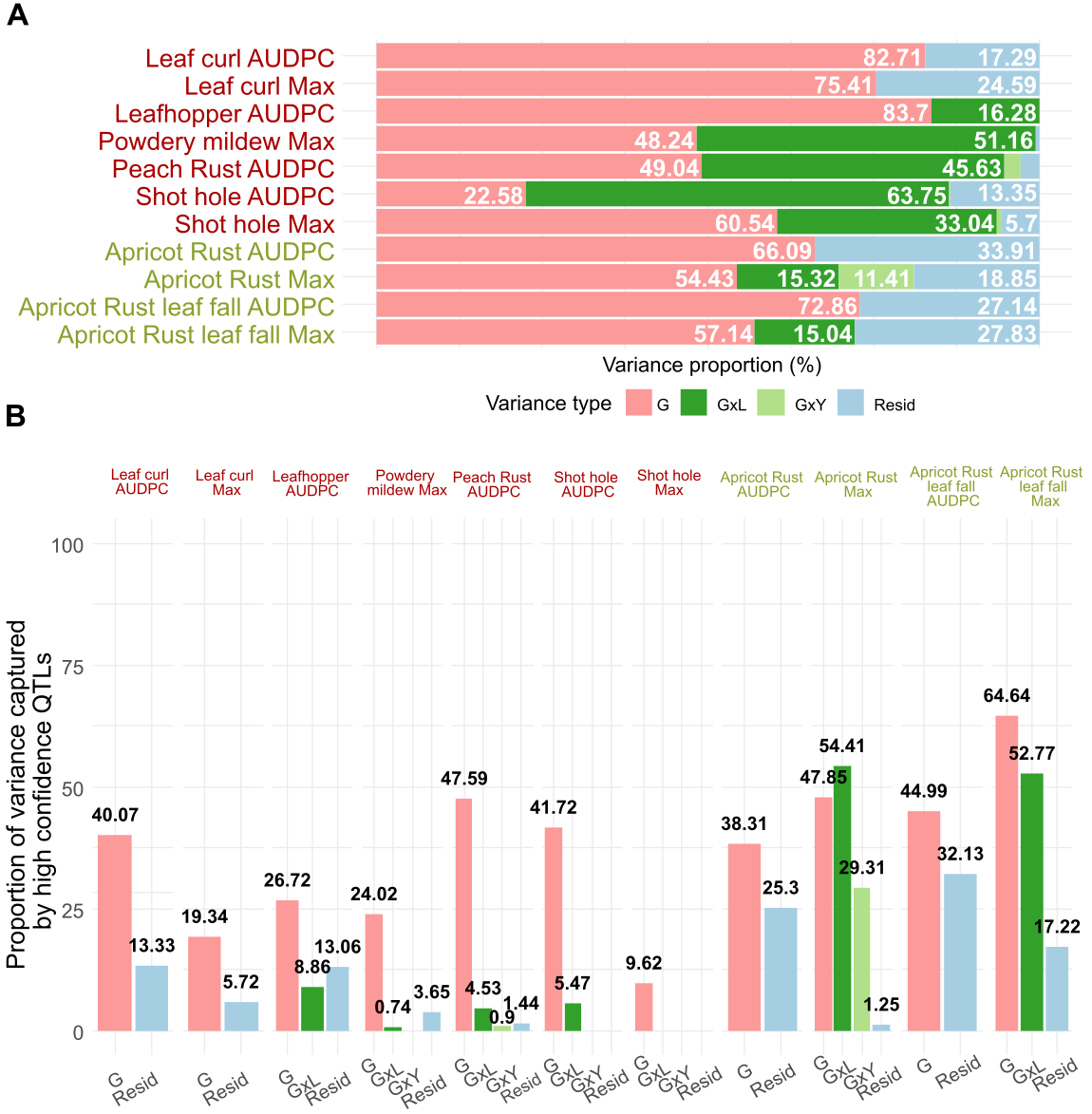


**Supplementary Fig 9.** A) Stacked bar plots with the variance of the random effects of G, G×L interaction, G×Y interaction and residuals, calculated from the model following Eq. 10. B) Proportions of variance of G, G×L interaction and G×Y interaction effects that were captured by the high confidence QTLs for the different pests and diseases, as computed from Eq.12.

**Supplementary Table 3.** List of genes with relevant functions for the high confidence QTLs

| Biotic stress | Trait | QTL Name | Candidate genes | Function |
| --- | --- | --- | --- | --- |
| Leaf curl | AUDPC and Max | Leaf_curl_4 | Prupe.4G009300 | disease resistance RPP13-like protein 4 |
|  |  |  | Prupe.4G026800 | LEA hydroxyproline-rich glycoprotein family |
|  |  |  | Prupe.4G026900 |  |
|  |  |  | Prupe.4G027000 |  |
|  |  |  | Prupe.4G027300 | Cysteine-rich receptor-like protein kinase |
|  |  |  | Prupe.4G027400 |  |
|  |  |  | Prupe.4G034300 | Disease resistance RPP13-like protein 4 |
|  |  |  | Prupe.4G038500 | Disease resistance protein At4g27220 |
|  |  |  | Prupe.4G048100 | Concanavalin A-like lectin protein kinase family protein |
| Leafhopper | AUDPC | Leafhopper_6 | Prupe.6G068800 | Stress signalling (protein phosphatase 2C) |
|  |  |  | Prupe.6G071800 | Plant adaptation to stresses (RING-type protein) |
|  |  |  | Prupe.6G071900 |  |
|  | AUDPC | Leafhopper_7_1 | Prupe.7G062200 | Disease resistance protein RGA2 |
|  |  |  | Prupe.7G062300 | Disease resistance protein RGA3 |
|  |  |  | Prupe.7G062500 | Disease resistance protein RGA1 |
|  |  |  | Prupe.7G062600 |  |
|  |  |  | Prupe.7G062700 |  |
|  |  |  | Prupe.7G062900 | LRR and NB-ARC domains-containing disease resistance protein |
|  |  |  | Prupe.7G063000 |  |
|  |  |  | Prupe.7G063100 | Leucine-rich receptor-like protein kinase family protein |
|  |  |  | Prupe.7G065300 | TMV resistance protein N |
|  |  |  | Prupe.7G065400 | Disease resistance protein (TIR-NBS-LRR class) |
|  |  |  | Prupe.7G065500 |  |
|  |  |  | Prupe.7G070800 | Knottin scorpion toxin-like domain-containing protein |
|  |  |  | Prupe.7G070900 |  |
|  |  |  | Prupe.7G071000 |  |
|  |  |  | Prupe.7G071100 |  |
|  |  |  | Prupe.7G071200 |  |
|  |  |  | Prupe.7G071300 |  |
|  |  |  | Prupe.7G071400 |  |
|  | AUDPC | Leafhopper_7_2 | Prupe.7G095800 |  |
| Powdery mildew | Max | Powdery_mildew_6 | Prupe.6G132100 | Disease resistance protein (TIR-NBS-LRR class) |
| Peach Rust | AUDPC | Peach_Rust_2 | Prupe.2G057600 | TMV resistance protein N |
|  |  |  | Prupe.2G057800 |  |
|  |  |  | Prupe.2G058400 | Ethylene-activated signalling pathway |
|  |  |  | Prupe.2G058400 |  |
|  |  |  | Prupe.2G059000 | TMV resistance protein N |
|  |  |  | Prupe.2G059200 |  |
|  |  |  | Prupe.2G059400 |  |
|  |  |  | Prupe.2G060200 | Ethylene-responsive transcription factor |
|  |  |  | Prupe.2G060400 | Disease resistance protein (TIR-NBS-LRR class) |
|  | AUDPC | Peach_Rust_4 | Prupe.4G035200 | sulphate assimilation |
|  |  |  | Prupe.4G038500 | Disease resistance protein At4g27220 |
|  |  |  | Prupe.4G041100 | Disease resistance-like protein CSA1 |
|  |  |  | Prupe.4G046600 | Zinc finger LSD1-type protein |
|  |  |  | Prupe.4G048100 | L-type lectin-domain receptor kinase |
|  |  |  | Prupe.4G051500 | Plant-type hypersensitive response |
|  |  |  | Prupe.4G053700 | MLO-like protein |
|  |  |  | Prupe.4G054500 | Transcription factor VOZ1 |
|  |  |  | Prupe.4G063300 | LRR receptor-like serine/threonine-protein kinase |
|  |  |  | Prupe.4G063500 |  |
|  |  |  | Prupe.4G063700 |  |
|  |  |  | Prupe.4G071500 | Serine-type endopeptidase activity |
|  |  |  | Prupe.4G072600 | eIF4e-like |
|  |  |  | Prupe.4G078600 | C2 domain-containing protein |
|  |  |  | Prupe.4G081000 | Calcyclin-binding protein |
|  |  |  | Prupe.4G082500 | Jasmonate ZIM containing protein |
|  |  |  | Prupe.4G082600 | Aldolase-type TIM barrel family protein |
|  |  |  | Prupe.4G083300 | Ornithine aminotransferase |
| Shot hole | AUDPC | Shot_hole_6 | Prupe.6G115400 | Protein argonaute 5 |
|  |  |  | Prupe.6G120300 | A/B barrel domain-containing protein At5g22580 |
|  |  |  | Prupe.6G120600 | MLO-like protein |
|  | Max | Shot_hole_8 | Prupe.8G247900 | RPH1 (resistance to phytophthora 1) |
|  |  |  | Prupe.8G257400 | MLO-like protein |
|  |  |  | Prupe.8G259700 | Co-chaperone protein |
|  |  |  | Prupe.8G262600 | Poly [ADP-ribose] polymerase |
|  |  |  | Prupe.8G264700 | Proline-rich protein 3 (response to ethylene) |
|  |  |  | Prupe.8G264800 |  |
| Apricot Rust | AUDPC | Apricot_Rust_2 | PruarM.2G180700 | disease resistance family protein, LRR family protein |
|  | AUDPC | Apricot_Rust_3.2 | PruarM.3G108000 | F-box family protein |
|  | AUDPC | Apricot_Rust_4.2 | PruarM.4G199900 | Leucine-rich receptor-like protein kinase family protein |
|  | Max | Apricot_Rust_4.4 | PruarM.4G204800 | Leucine-rich receptor-like protein kinase family protein |
|  | AUDPC | Apricot_Rust_4.5 |  |  |
|  | AUDPC | Apricot_Rust_4.6 |  |  |
|  | AUDPC | Apricot_Rust_4.7 |  |  |
|  | AUDPC | Apricot_Rust_6.1 | PruarM.6G017900 | Receptor-like protein kinase 1 |
|  |  | Apricot_Rust_6.2 |  |  |
| Apricot Rust Leaf fall | AUDPC and Max | Apricot_Rust_leaf_fall_2.1 | PruarM.2G251100 | Disease resistance protein RGA2 |
|  | Max | Apricot_Rust_leaf_fall_2.2 | PruarM.2G283800 | TMV resistance protein N |
|  | Max | Apricot_Rust_leaf_fall_2.3 | PruarM.2G352900 | WD-40 repeat family protein |
|  | Max | Apricot_Rust_leaf_fall_2.4 | PruarM.2G359800 | Cysteine-rich receptor-like kinase |
|  | Max | Apricot_Rust_leaf_fall_2.5 | PruarM.2G359900 | Cysteine-rich receptor-like kinase |
|  | AUDPC | Apricot_Rust_leaf_fall_4.1 | PruarM.4G156300 | Lectin protein kinase family protein |
|  | Max and AUDPC | Apricot_Rust_leaf_fall_4.6 | PruarM.4G199400 | Leucine-rich receptor-like protein kinase family protein |
|  | Max and AUDPC | Apricot_Rust_leaf_fall_4.7 | PruarM.4G199700 | Leucine-rich receptor-like protein kinase family protein |
|  | Max and AUDPC | Apricot_Rust_leaf_fall_4.8 | PruarM.4G199900 | Leucine-rich receptor-like protein kinase family protein |
|  | Max and AUDPC | Apricot_Rust_leaf_fall_4.10 | PruarM.4G201600 | BURP domain-containing protein |
|  | Max and AUDPC | Apricot_Rust_leaf_fall_4.16 | PruarM.4G204800 | Leucine-rich receptor-like protein kinase family protein |
|  | Max and AUDPC | Apricot_Rust_leaf_fall_4.17 |  |  |
|  | Max and AUDPC | Apricot_Rust_leaf_fall_4.18 |  |  |
|  | Max | Apricot_Rust_leaf_fall_4.21  Apricot_Rust_leaf_fall_4.22 | PruarM.4G206100 | Leucine-rich receptor-like protein kinase family protein |
|  | Max and AUDPC |  |  |  |
|  | Max | Apricot_Rust_leaf_fall_6.1  Apricot_Rust_leaf_fall_6.2  Apricot_Rust_leaf_fall_6.3 | PruarM.6G017900 | Receptor-like protein kinase 1 |
|  | Max and AUDPC |  |  |  |
|  | Max |  |  |  |
|  | Max and AUDPC | Apricot_Rust_leaf_fall_6.5 | PruarM.6G018400 | Receptor-like protein kinase 1 |


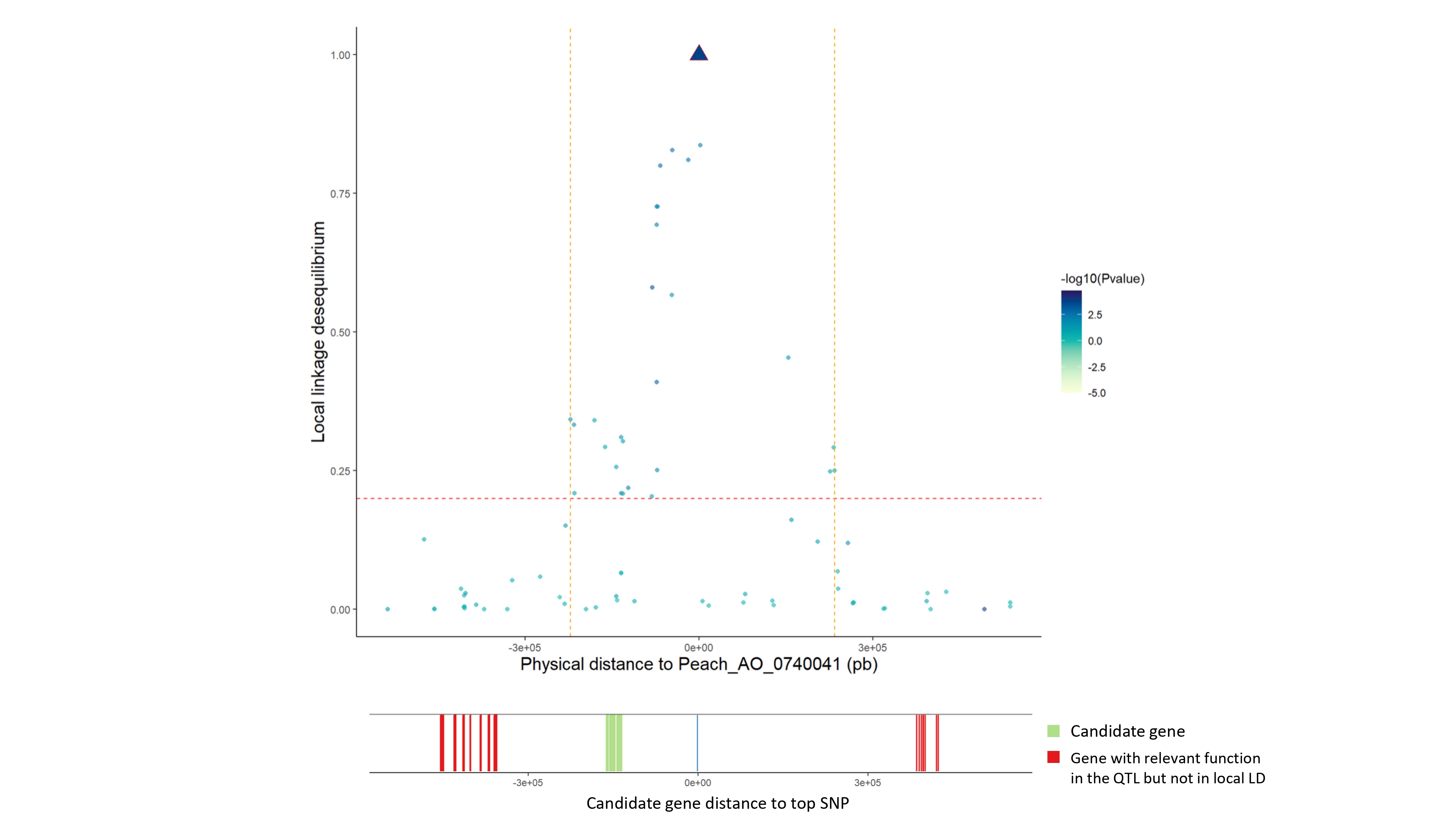

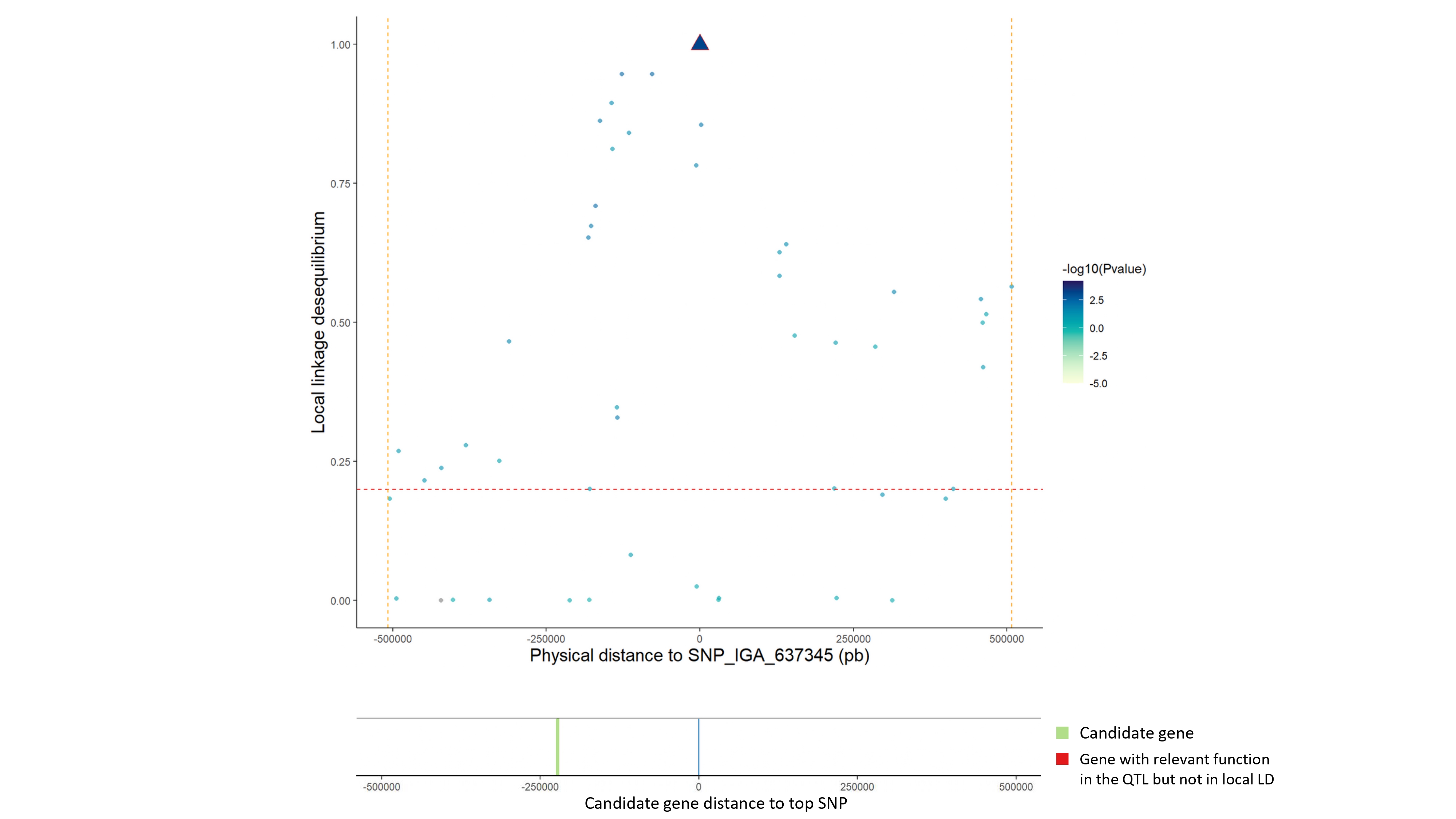

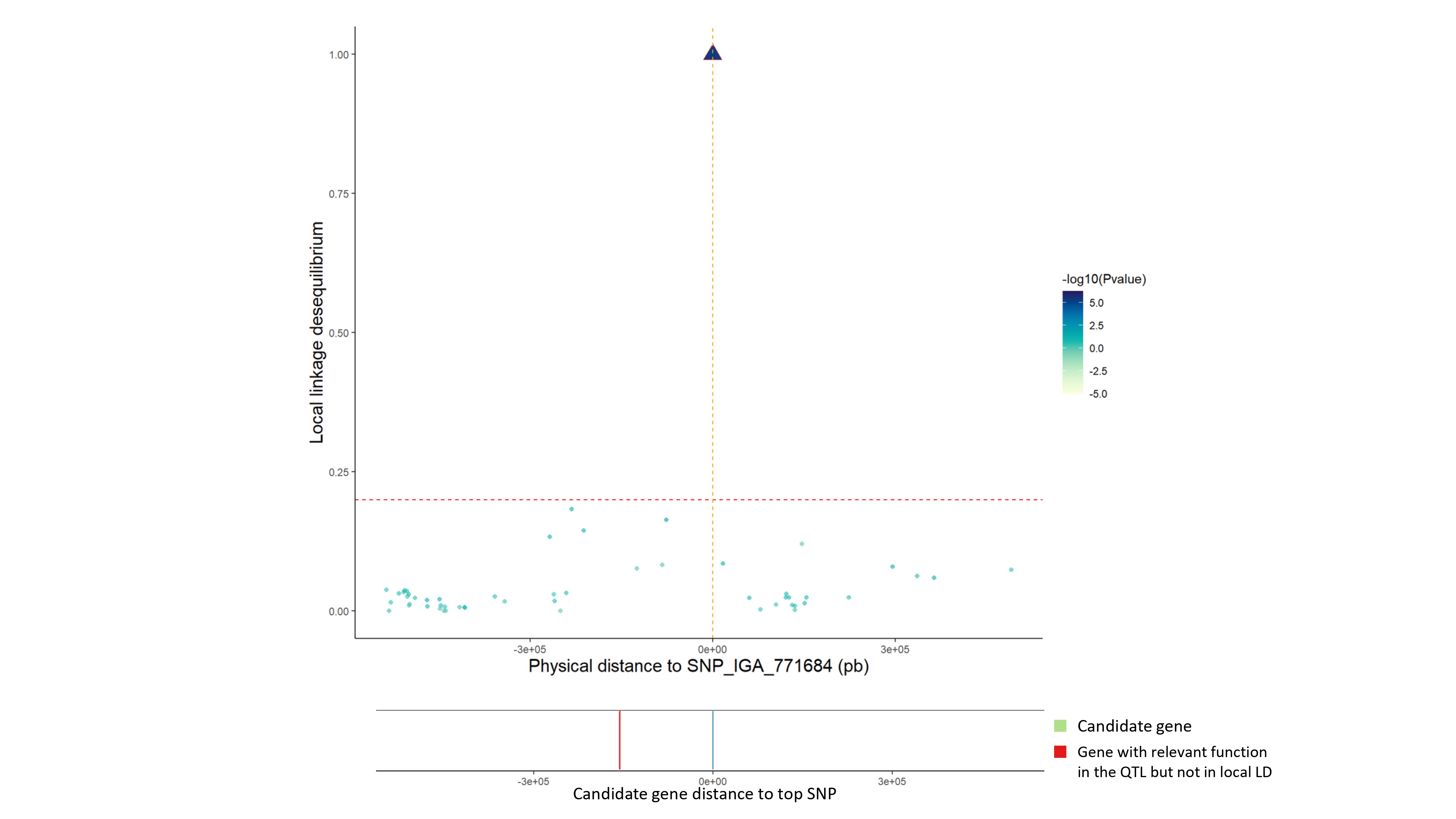

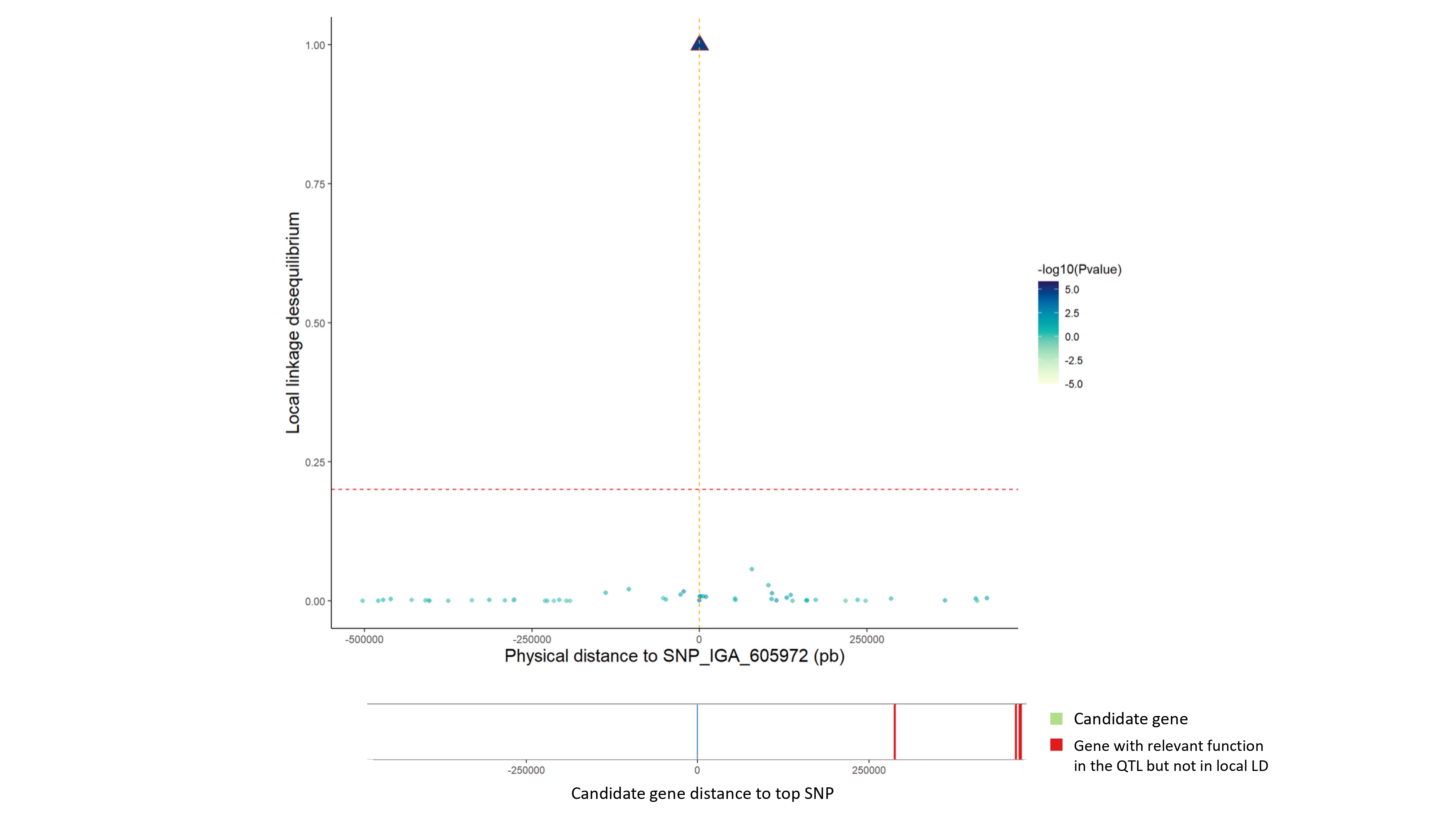


**A**

**C**als

**D**als

**B**als

**F**als

**E**als


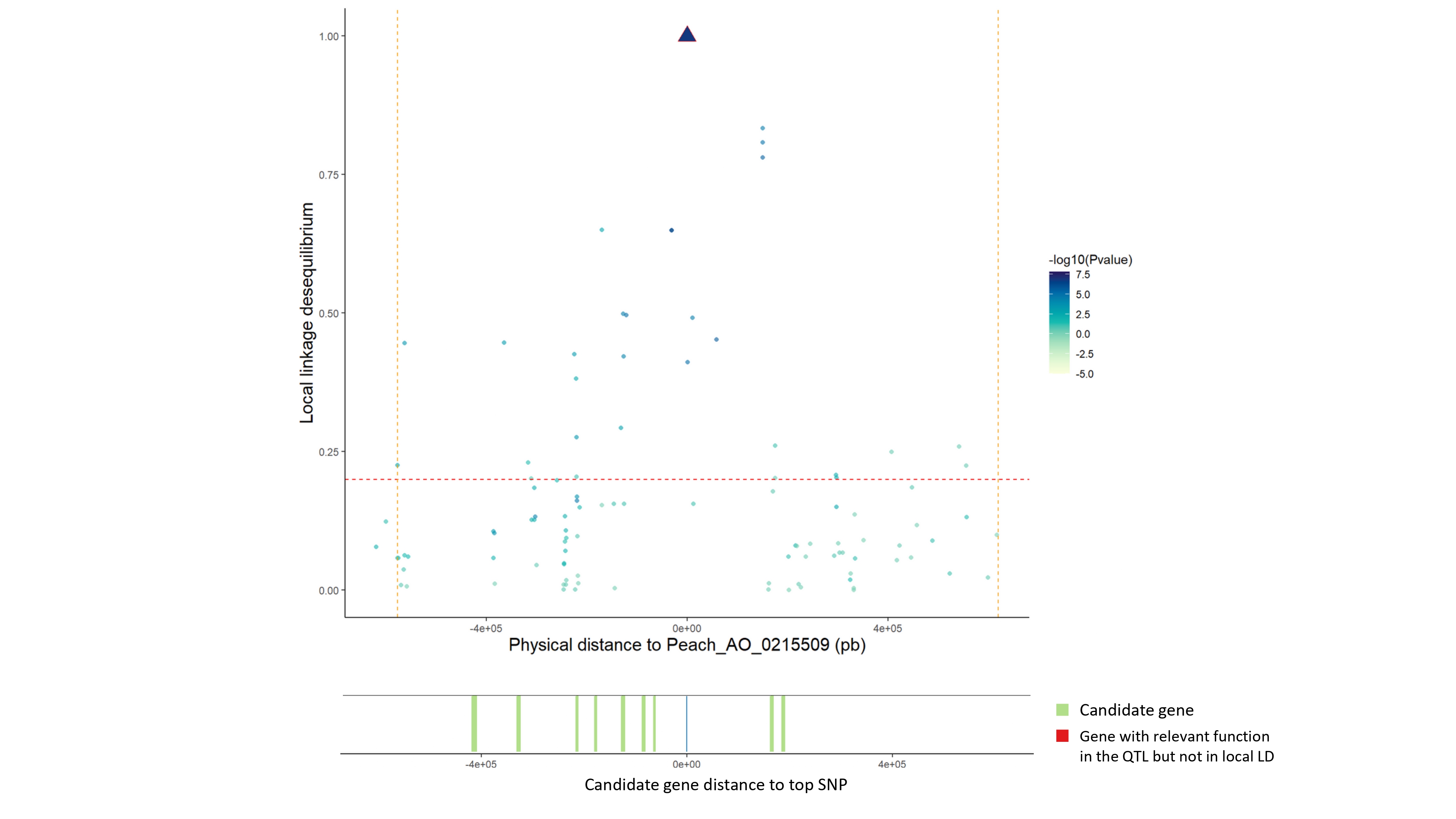

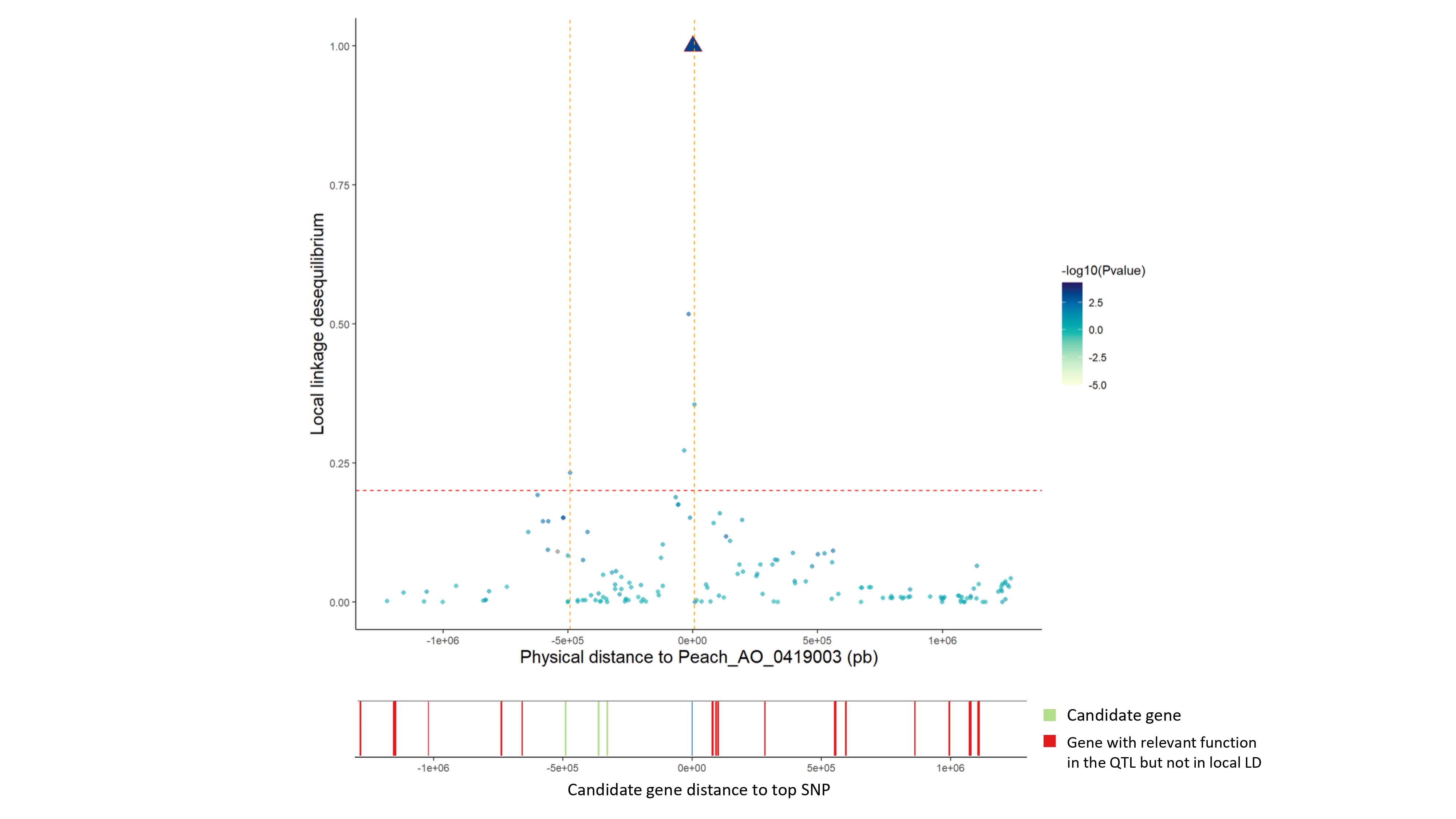


**Supplementary Fig 10.** Representation of the local LD between the top SNP of the studied QTL and all other SNPs of the QTL (top) and candidate genes located in the QTL (bottom) in peach for A) Leafhopper_6, B) Leafhopper_7_1, C) Leafhopper_7_2, D) Powdery_mildew_6, E) Peach_Rust_2, F) Peach_Rust_4, G) Shot_hole_6 and H) Shot_hole_8.

For the top part of the figure, the X-axis represents the physical distance from the top SNP, while the Y-axis represents the r2, corrected by the relatedness and structure, between SNPs and the top SNP. Each dot represents a SNP which is coloured according the P-value of Meta-GWAS. The vertical orange dot lines represent the QTL interval readjusted using the local LD. In the lower part of the figure, genes with relevant functions located in the QTL, candidate genes are in green and genes with relevant functions located in the QTL but not in LD with the top SNP are in red


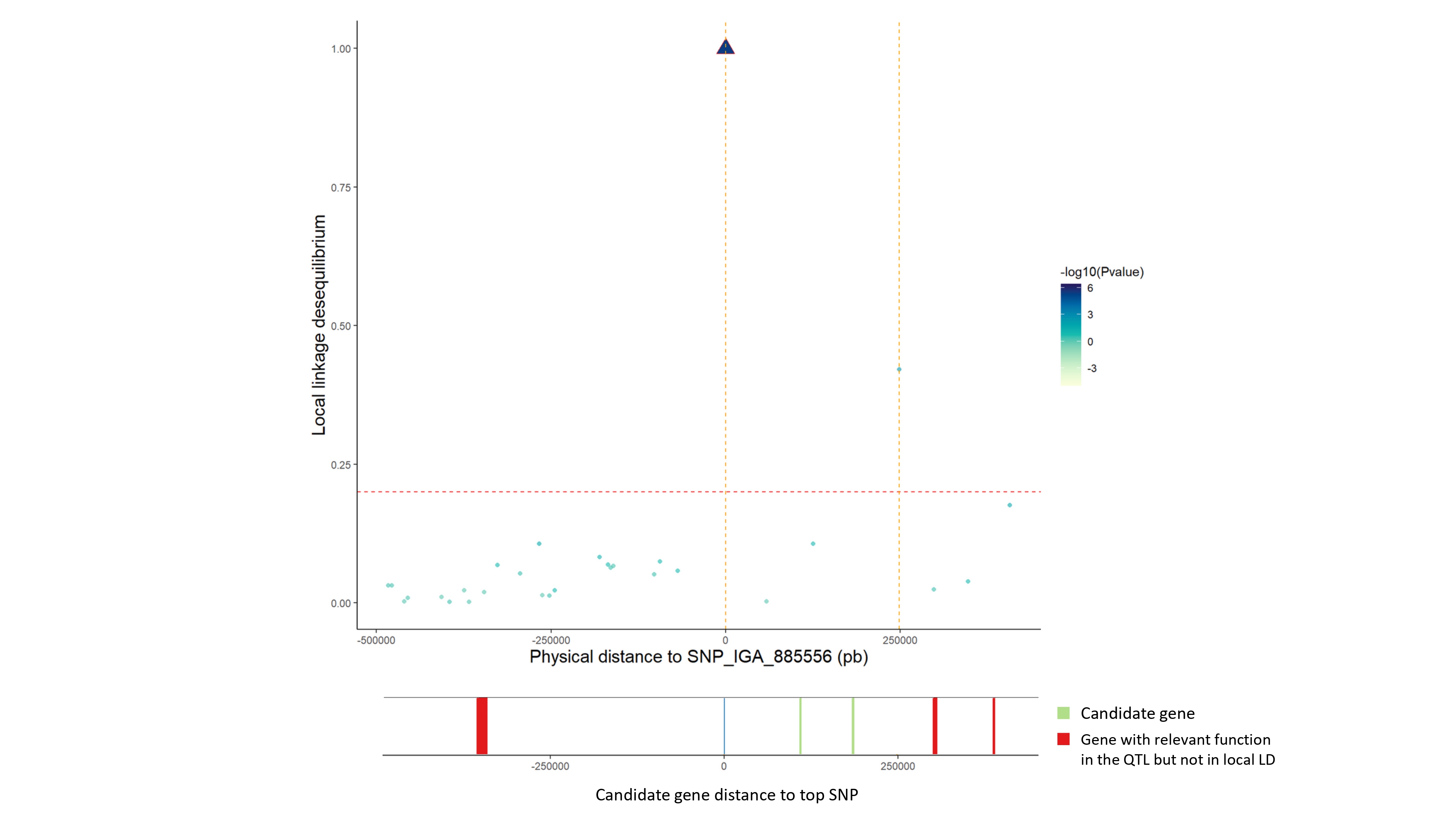

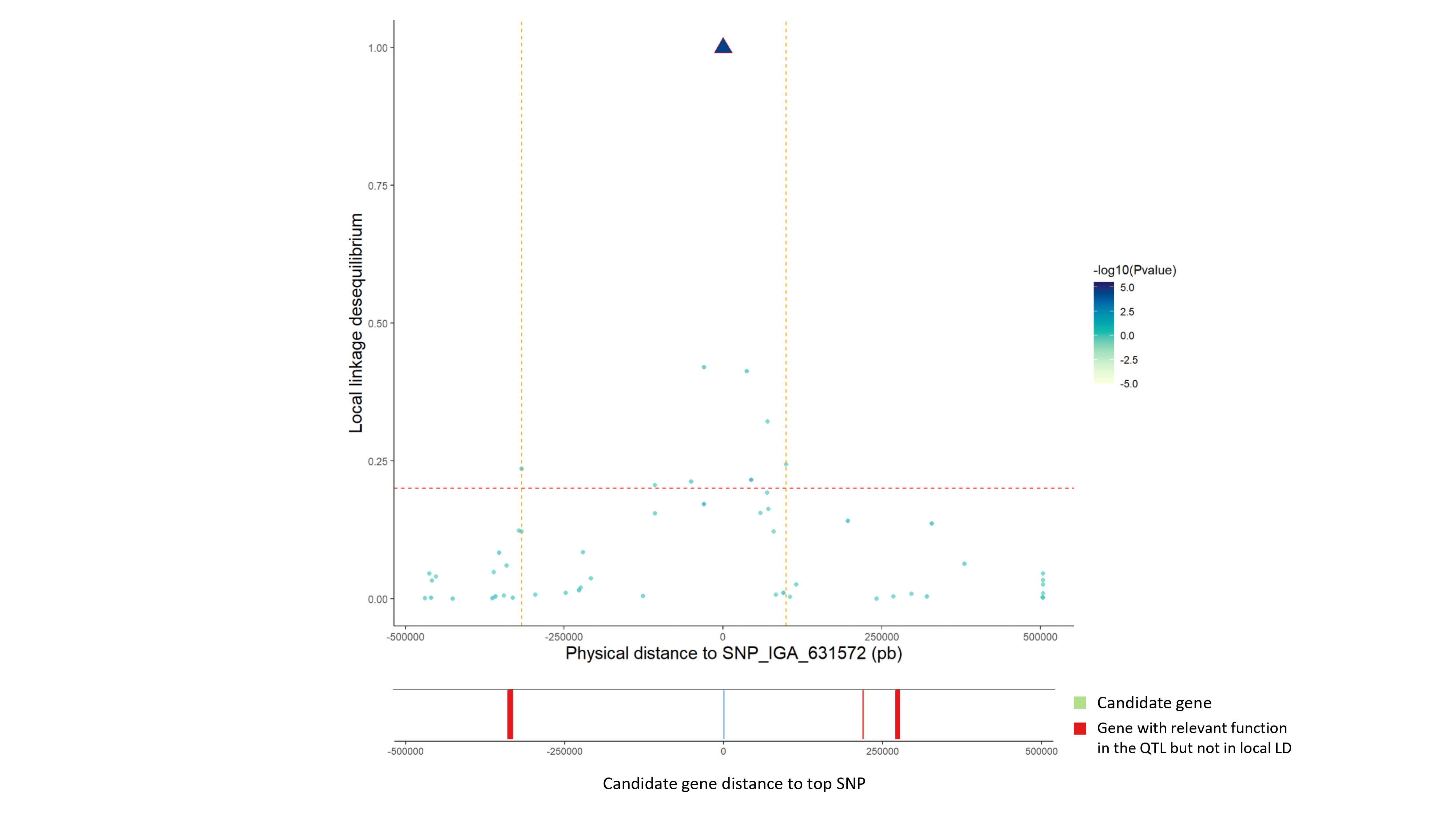


**H**als

**G**als


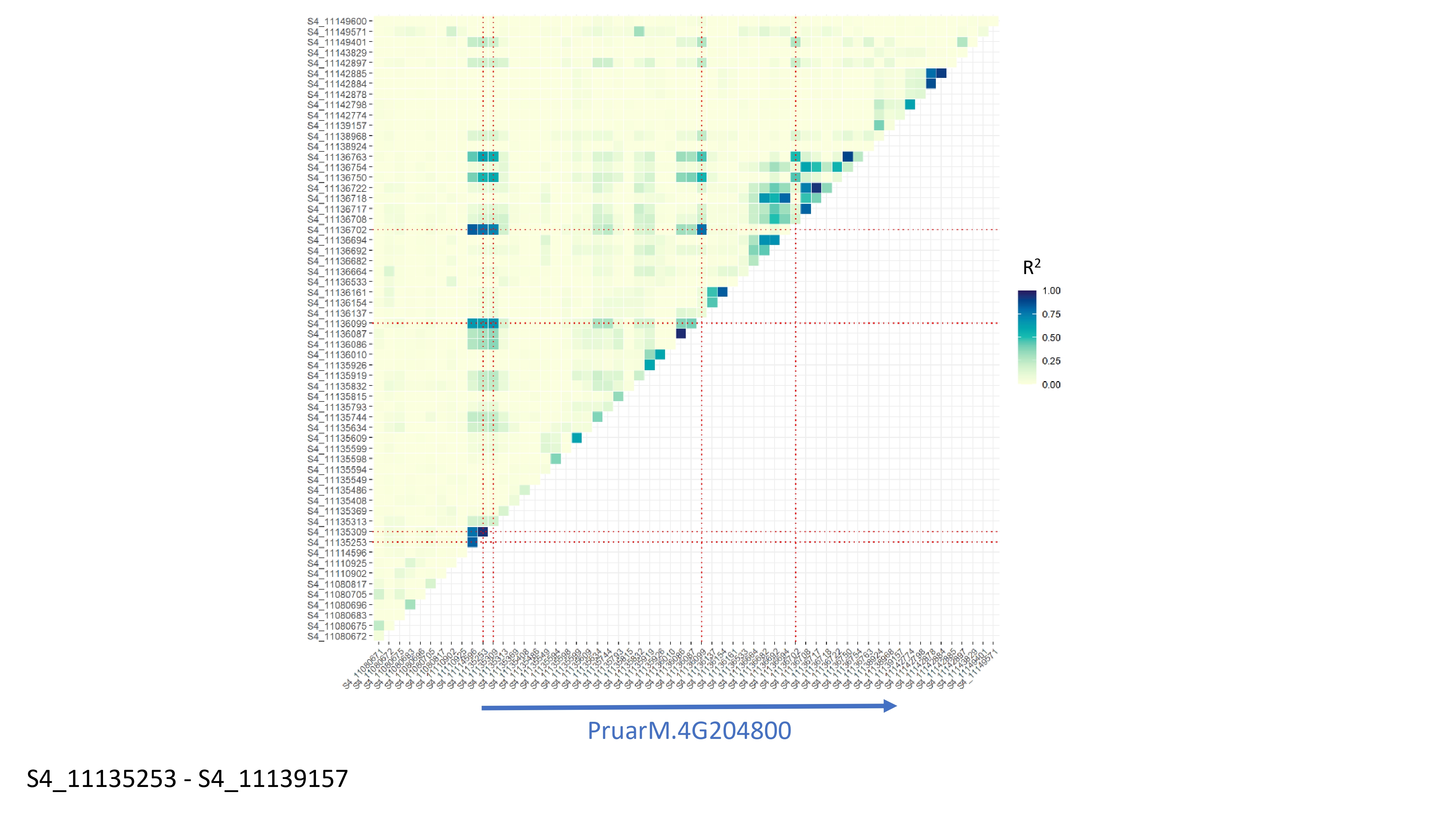

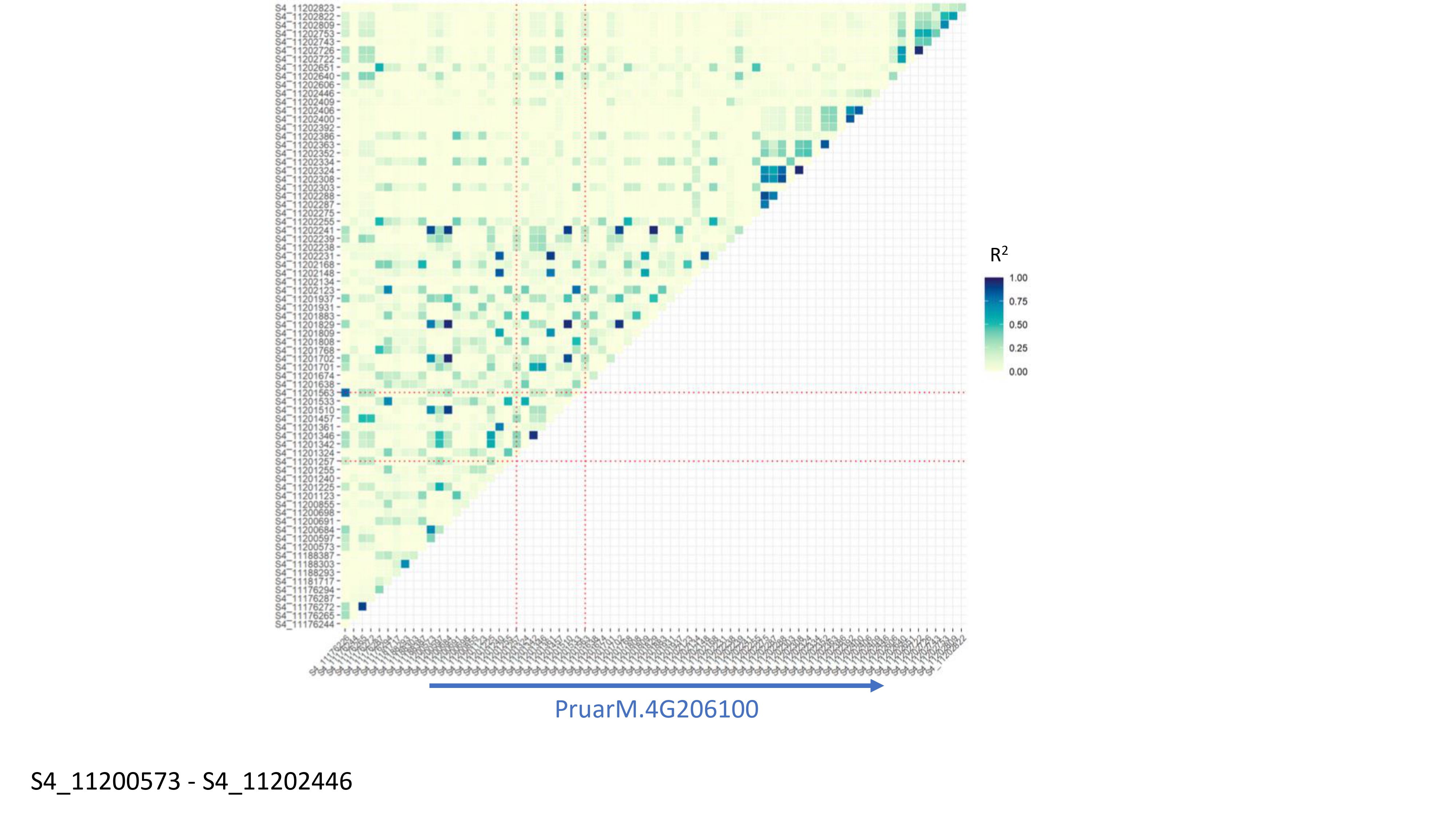

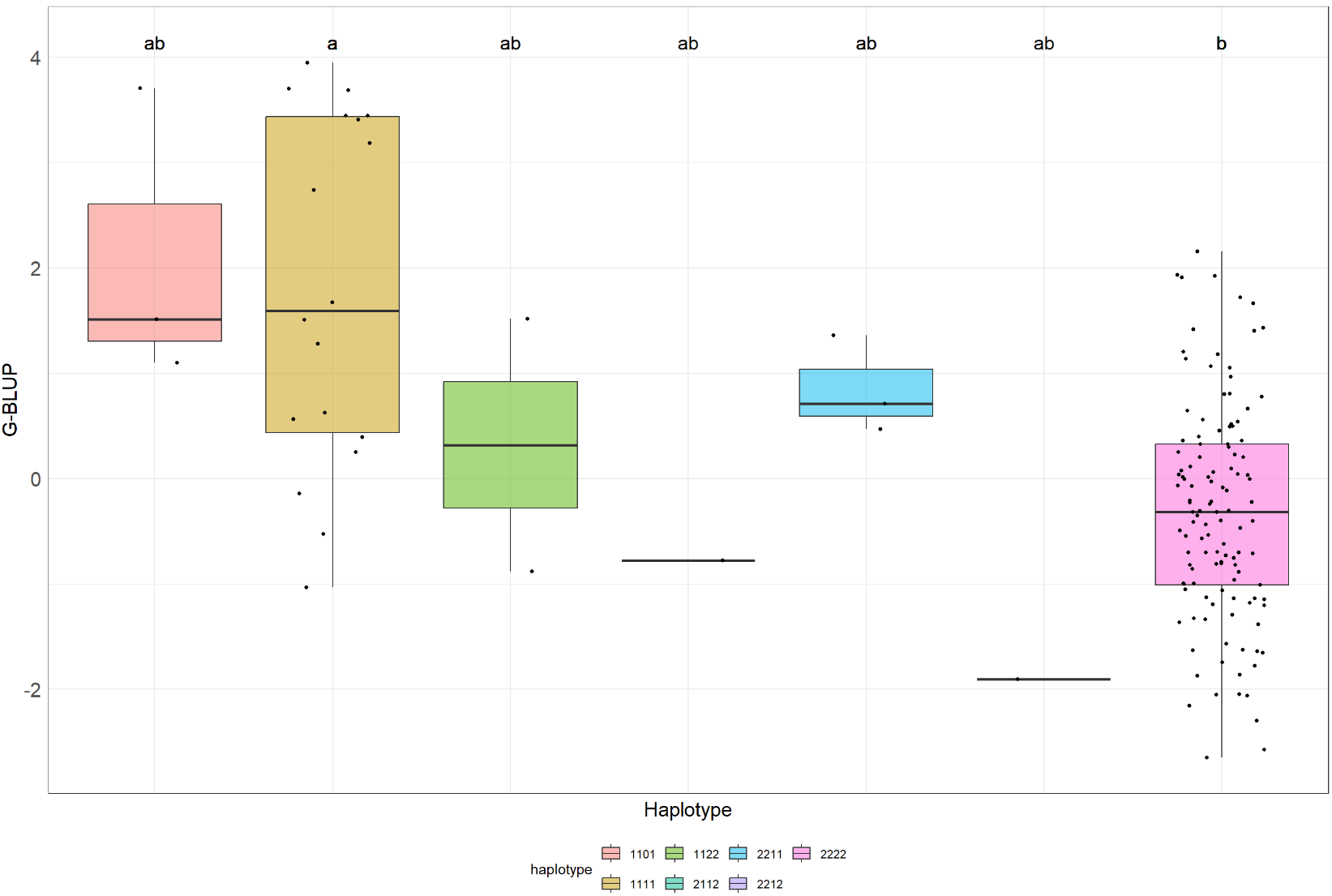

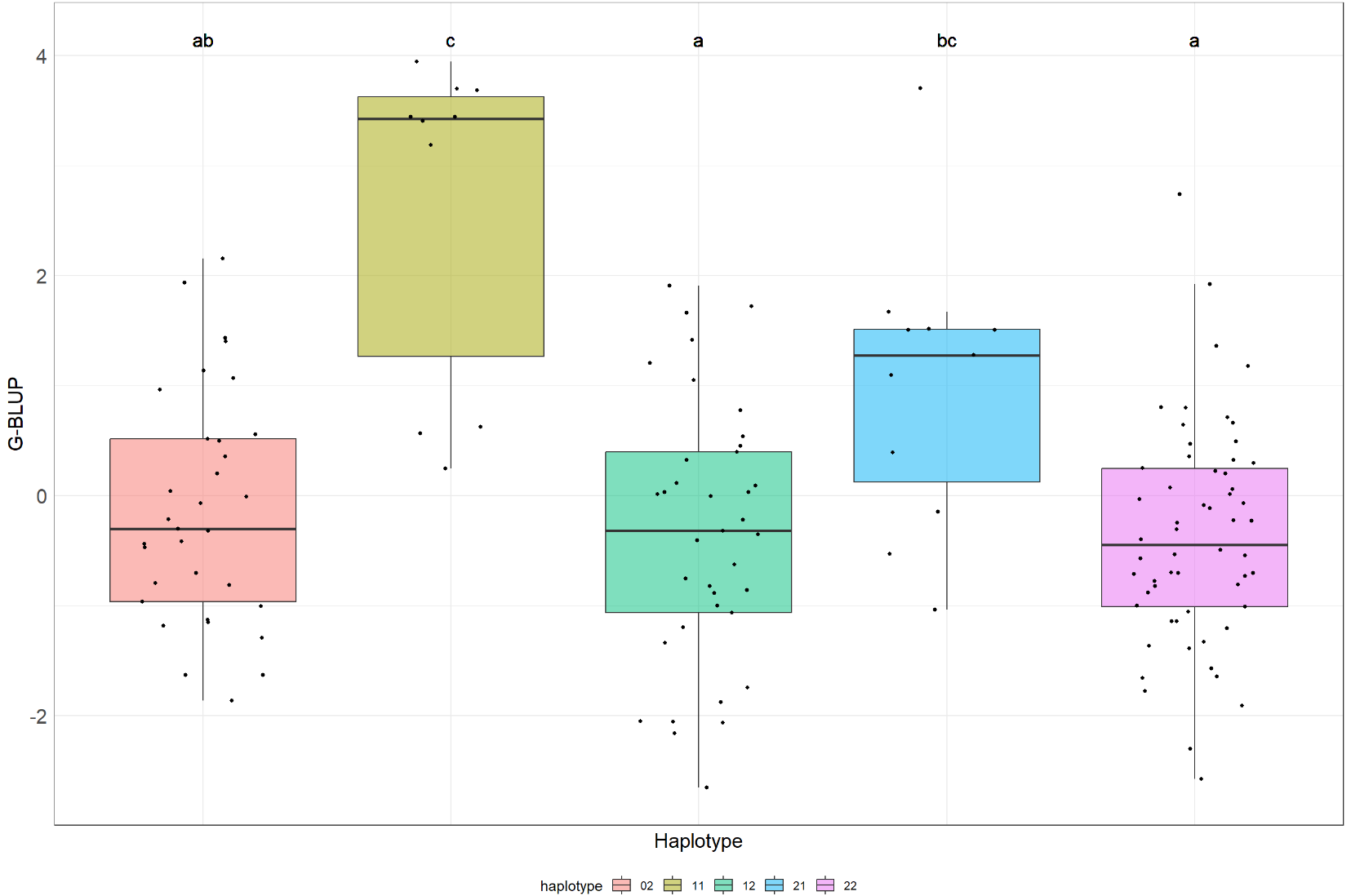


**A**als

**C**als

**D**als

**B**als

**Supplementary Fig 11.** For the top part of the figure, heatmaps of pairwise LD estimates within the genomic window around candidate gene A) ‘PruarM.4G204800’ and B) ‘PruarM.4G206100’. Top SNPs of High confidence QTLs are shown with red dot line. In the lower part of the figure, G-BLUPs distributions according to the haplotype constituted by the SNPs C) ‘S4_11135253’, ‘S4_11135309’, ‘S4_11136099’, ‘S4_11136702’and D) ‘S4_11201257’, ‘S4_11201563’. Statistical differences between groups were computed by pairwise Wilcoxon tests.

**Supplementary Table 4**. Equivalence between allelic state and numerical transformation of genotypic dataset for the different additive and non-additive models. a2 correspond to the reference allele and a1 to the alternative allele. Note that dominant and recessive models can correspond to both dominant and recessive genotype-phenotype interaction, but that we had to differentiate the effects of a dominant reference allele or a dominant alternative allele for GWAS analysis.

| Allelic state | Additive model | Dominant model | Recessive model | Overdominance model |
| --- | --- | --- | --- | --- |
| a1a1 | 0 | 0 | 0 | 0 |
| a1a2 | 1 | 2 | 0 | 1 |
| a2a2 | 2 | 2 | 2 | 0 |

Supplementary Methods

**Supplementary Method 1. Pests and disease rating scales**

The assessment of the damages caused by all pests and diseases was based on the visual estimation of symptom incidence (percentage of damaged leaves). Different rating scales were used to best capture the phenotypic expression of each pest or disease (Table 1 to Table 5). For some pests and diseases, the damages were scored with two variables describing the incidence (percentage of damaged leaves) and the severity (average percentage of symptoms per affected leaf) of symptoms (Table 3 and 4). The product between intensity and severity gives an integrative trait that was considered in the following analysis. Each rating class has an equivalent in percentage in terms of the range of organs affected (for example, a ‘2’ for powdery mildew indicates that between 1% and 10% of leaves show symptoms.). Therefore, for the following analyses, these damage scores were transformed in percentage values (corresponding to the centre of each class) in order to work with quantitative data which can be comparable between each pests or diseases monitored.

**Table 2.** Rating scale used for leafhopper and powdery mildew

| Note | **Incidence**  **(percentage of damaged leaves)** |
| --- | --- |
| 0 | 0 % (no symptoms) |
| 1 | 1 à 10 % |
| 2 | 11 à 20 % |
| 3 | 21 à 30 % |
| 4 | 31 à 40 % |
| 5 | 41 à 50 % |
| 6 | 51 à 60 % |
| 7 | 61 à 70 % |
| 8 | 71 à 80 % |
| 9 | 81 à 90 % |
| 10 | > 90 % |

**Table 1.** Rating scales used for leaf curl

| Note | **Incidence**  **(percentage of damaged leaves)** |
| --- | --- |
| 1 | 0 % (no symptoms) |
| 2 | 1 à 10 % |
| 3 | 11 à 25 % |
| 5 | 26 à 50 % |
| 7 | 51 à 75 % |
| 9 | > 75 % |

**Table 3.** Rating scale used for peach rust and shot hole

| Note | **Incidence**  **(percentage of damaged leaves)** | **Severity**  **(average percentage of symptoms per affected leaf)** |
| --- | --- | --- |
| 1 | 0 % (no symptoms) | 0 % (no symptoms) |
| 2 | 1 à 10 % | 1 à 10 % of leaf area |
| 3 | 11 à 25 % | 11 à 25 % |
| 5 | 26 à 50 % | 26 à 50 % |
| 7 | 51 à 75 % | 51 à 75 % |
| 9 | > 75 % | > 75 % |

**Table 4**. Rating scale used for apricot rust

| Note | **Incidence**  **(percentage of damaged leaves)** | **Severity**  **(average percentage of symptoms per affected leaf)** |
| --- | --- | --- |
| 0 | 0 % (no symptoms) | 0 % (no symptoms) |
| 1 | ≤ 10 % | ≤ 10 % |
| 2 | >10 et ≤ 30 % | >10 et ≤ 30 % |
| 3 | >30 et ≤ 60 % | >30 et ≤ 60 % |
| 4 | >60 et ≤ 80 % | >60 et ≤ 80 % |
| 5 | >80 % | >80 % |

| Note | **Fall**  **(percentage of leaf fall due to rust)** |
| --- | --- |
| 0 | 0 % (no symptoms) |
| 1 | ≤ 10% |
| 2 | >10% et ≤ 30 % |
| 3 | >30% et ≤ 60 % |
| 4 | >60% et ≤ 80 % |
| 5 | >80 % |

**Table 5**. Rating scale used for apricot rust leaf fall

For blossom blight, the assessment was performed around 30 days after blooming date, in such a way that all symptoms are expressed and well visible before the development of leaves. All shoots infected by *Monilinia* spp. were pruned after the observation. The incidence of blossom blight was estimated by assessing the proportion of the length of flowering branches dried out by *Monilinia* spp. in relation to the total length of flowering branches on the tree.

**Supplementary Method 2. Spatial correction within environments**

At each date of observation, phenotypic data were adjusted within each environment to correct for spatial environmental effects with the following model using R/lme4 [1] :

$$\begin{aligned} Y_{tirc}= \mu+\underline{G_{i}}+\underline{R_{r}}+\underline{C_{c}}+ \underline{\varepsilon_{tirc}}\#(1) \end{aligned}$$

$$with G \sim N\left( 0, \sigma_{g}^{2}I \right)$$

$$R \sim N\left( 0, \sigma_{r}^{2}I \right)$$

$$C \sim N\left( 0, \sigma_{c}^{2}I \right)$$

$$\varepsilon\sim N\left( 0, \sigma_{\varepsilon}^{2}I \right)$$

where $Y_{tirc}$ the phenotypic value of tree $t$ from accession$i$ measured at row $r$ and column $c$*,* μ the overall mean, $G_{i}$is the effect of accession$i$, $R_{r}$the effect of row $r$,$C_{c}$ the effect of column $c$ and$\varepsilon_{tirc}$ the error. All effects are assumed to be random.

The AIC [2] of the model (1) and of the null model (with only the genotype effect) was calculated and the model with the lowest AIC was conserved. If the model (1) was the best, the best linear unbiased predictors (BLUPs) of the row and column effects were subtracted from the raw phenotypic values to obtain corrected field performances. Hereafter we note $\bar{Y}_{tily}$the corrected field performance of tree $t$ from accession $i$ in location $l$ and year $y$. Spearman correlation [3] was calculated on this corrected phenotypic value at the scale of individual (*i.e.* trees) for each environment and each pests and diseases observed.

We used two approaches to account for the kinetic observations performed within environments (except blossom blight). The first approach aimed at catching the potential susceptibility of each tree by retaining the maximum damage score for each pests and diseases monitored (latter called Max). The second approach is more integrative and aims at catching the phenotypic trajectories of each individual tree using the Area under the Disease Progress Curve (AUDPC). AUDPC is the area that is determined by the sum of trapezes under the curve and was calculated according to [4] and using R/agricolae. Of note, AUDPC could only be calculated for trees which have been monitored several times during the season and at each date of observation.

The initial examination of the phenotypic distribution of Max after spatial correction revealed important variation of standard deviations depending on the biotic stress and on the environment, with few environments presenting particularly low values for specific pest or disease (Supplementary Fig 1). Indeed, depending on the environment, inoculum pressure can fluctuate considerably and some environments could be non-favourable for disease development. We decided to consider only environments where the pests or diseases were sufficiently expressed and where the phenotypic diversity of symptoms expression was high enough, *i.e.* environment for which the standard deviation of Max after spatial correction was higher than 9. In total, 10 combinations of phenotype by environments have been excluded from analysis among the 51 available (Supplementary Fig 1).

Prior to genetic analyses, Max and AUDPC variables were transformed by using a Box-Cox transformation [5] to correct for heteroscedasticity and non-normality of error terms. Hereafter we note $Trans \bar{Y}_{tily}$the transformed field performance of tree $t$ from accession $i$ in location $l$ and year $y$.

**Supplementary Method 3. Phenotypic analysis: variance partition, heritability and BLUPs computation**

A multi-environment mixed model integrating the effect of the environment ($Env$) (as fixed factor), the Genotype (G), the Genotype by Location (G x L), the Genotype by Year (G x Y) and the Genotype by Location by Year (G x L x Y) interactions (as random factors) was fitted to determine the contribution of the genetic and environmental factors to the observed variations. Performed with R/lme4 [1], this model can be described as follows:

$$\begin{aligned} Trans \bar{Y}_{tily}= \mu+{Env}_{ly}+ \underline{G_{i}}+ \underline{{GL}_{i,l}}+\underline{{GY}_{iy}}+\underline{{GLY}_{ily}}+ \underline{\varepsilon_{tily}}\#(2) \end{aligned}$$

$$with G_{i}\sim N\left( 0, \sigma_{G}^{2} \right), {GL}_{il}\sim N\left( 0, \sigma_{GL}^{2} \right),{GY}_{iy}\sim N\left( 0, \sigma_{GL}^{2} \right), {GLY}_{ily}\sim N\left( 0, \sigma_{G\mathrm{LY}}^{2} \right) and \varepsilon_{tily}\sim N\left( 0, \sigma_{\varepsilon}^{2} \right)$$

where $Trans \bar{Y}_{tily}$ is the transformed and corrected phenotypic value of tree $t$ from accession$i$, in location $l$ , and year$y$ *,* μ the overall mean, ${Env}_{ly}$ the fixed effect of environment $l\times y$, $G_{i}$the random effect of the accession$i$,${GL}_{il}$ the interaction term between location $l$and accession$i$, ${GY}_{iy}$ the interaction term between year $y$ and accession $i$ , ${GLY}_{iy}$ the interaction term between location$l$ , year $y$ and accession $i$ and$\varepsilon_{tily}$the random independent and identically distributed residual term.

REML estimates of genetic ($\sigma_{G}^{2}$), interaction between genotype and location ($\sigma_{GL}^{2}$), interaction between genotype and year of observation ($\sigma_{GY}^{2}$), interaction between genotype, location and year of observation ($\sigma_{GLY}^{2}$) and residual ($\sigma_{\varepsilon}^{2}$) variances were computed from this multi-environment mixed model. Broad-sense heritability H^2^ for each studied trait, was calculated as follows [6,7]:

$$\begin{aligned} H^{2}=\frac{\sigma_{G}^{2}}{\sigma_{G}^{2}+ \frac{\sigma_{GL}^{2}}{n_{L}}+\frac{\sigma_{GY}^{2}}{n_{Y}}+\frac{\sigma_{G\mathrm{LY}}^{2}}{n_{LY}}+\frac{\sigma_{\varepsilon}^{2}}{n_{R}}} \#\left( 3 \right) \end{aligned}$$

where n_L_, n_Y_, n_LY_,and n_R_ are the harmonic means for respectively the number of locations per accession, the number of years per accession, the number of environments per accession and the number of replications per environment per accession.

Best unbiased linear predictors (BLUPs) were estimated from this multi-environment model to reflect global genetic effects (G-BLUPs) and environment-specific effects (environment-specific BLUPs, incorporating predictors of G, G x L, G x Y and G x L x Y random effects). These predictors were then used as phenotypic input data for the genome-wide association studies (GWAS).

For each pest or disease, genetic correlations between pairs of environments were calculated using the Pearson correlations between environment-specific BLUPs.

**Supplementary Method 4. Variance partition for non-additive effects**

Before performing non-additive GWAS, variance decomposition was done to estimate the proportion of additive and non-additive genetic variance components. We used the model proposed by [8] and [9] to partition accessions genetic variance into additivity and dominance deviation terms. Variance decomposition was performed on G-BLUPs values with the MM4LMM R package [10].

**Supplementary Method 5. Population structure and relatedness analyses**

Prior to the structure analysis of the apricot core collection, we performed a pruning procedure of the imputed and filtered SNPs matrix which was based on the linkage disequilibrium. We used PLINK 2.0 (<https://www.cog-genomics.org/plink/2.0/>) with the following parameters: "--indep-pairwise 50 5 0.2”. This resulted in the selection of 41,145 independent SNP markers (linkage disequilibrium <0.2).

The additive allelic dosage at each marker was used to determine the genetic structure of the apricot core collection. To this aim, three complementary analyses were performed: i) principal component analysis (PCA, R-package ade4, [11]), ii) discriminant analysis of principal components (DAPC, R-package Adegenet, [12]) and iii) sparse non-negative matrix factorization (SNMF, R-package LEA,[13]). For DAPC and SNMF analyses, we tested a range of hypothetical group number from 1 to 10 (K value). To determine the optimum number of genetic groups, we used the K-means and the Evanno method [14] on DAPC and SNMF results respectively.

**Supplementary Method 6. Multi-environment GWAS**

We performed single locus multi-environment GWAS by using the multi-trait mixed model (MTMM) as described by [15] in order to distinguish common and environment-specific marker–phenotype association. This model can be described as follows:

$$\begin{aligned} Y=\left[ \begin{matrix} y_{1} \\ \ldots\\ y_{n} \end{matrix} \right]=\sum_{i=1}^{n} s_{i}\mu_{i} +X\beta+(X \times S )\alpha+ \upsilon\#(4) \end{aligned}$$

Where $y_{k}$ is the vector of environment-specific BLUPs for the environment $k$ ,$s_{i}$ is a vector having 1 for values belonging to the $i$’th environment and 0 otherwise,$\mu_{i}$ is the mean environmental effect , $X$ is the molecular marker score matrix,$\beta$ is the vector of main marker effects, $S$ is a design matrix for environmental effects resembling all $s_{i}$ vectors, α is the vector of interaction effects between markers and environments and$\upsilon\sim N(o,\sum_{G} \otimes K+R)$ is a random variable comprising of both random genetic and residual effects. $K$ is a kinship matrix, $R$ is a diagonal block matrix and $\sum_{G}$is a between environment variance-covariance matrix calculated as follows:

$$\begin{aligned} \sum_{G} = \left[ \begin{matrix} \sigma_{11}^{2} & \cdots& \sigma_{n1}^{2} \\ \vdots& \ddots& \vdots\\ \sigma_{1n}^{2} & \cdots& \sigma_{nn}^{2} \end{matrix} \right]\#(5) \end{aligned}$$

$\sum_{G}$ was calculated using the unstructured covariance specification in BreedR allowing for unequal genotype variances and unique covariances for each pair of environments [16].

Generalized least square (GLS) F-tests were used to estimate the genome-wide marker effect. Depending on what kind of SNP we were interested, three different F-tests were performed:

- The ‘full model’ which tested the model with both $\beta\neq0$ and $\alpha\neq0$ against a null model where $\beta=0$ and $\alpha=0$ to identify SNPs with both stable and interaction effects in the different environment;
- The ‘common model’ which tested the model with $\beta\neq0$ and $\alpha= 0$ against a null model where $\beta=0$ and $\alpha=0$ to identify SNPs with the same effect in all environments;
- The ‘gei model’ which tested the model with both $\beta\neq0$ and $\alpha\neq0$ against a reduced model where $\beta\neq0$ and $\alpha=0$, to identify SNPs having distinct effects in the environments.

**Supplementary Method 7. Meta-analysis GWAS**

Based on different single-environment GWAS studies testing the associations between a set of $M$ markers and a phenotype of interest, the first step of the MetaGE procedure relies on the calculation of Z-scores ($Z_{m}$) from the effect and the *P*-values of individual markers in the single-environment GWAS.

Since our dataset is obtained under natural environmental conditions, we used the random procedure from the MetaGE package which accounts for the heterogeneity of the QTL effects across environments and the correlation between the single-environment GWAS. This procedure allows coping with unbalanced and incomplete datasets without any further data imputation.

This model can be described as follows:

$$\begin{aligned} Z_{m}= \mu_{m}\mathbb{1}_{K}+ A_{m}+ E_{m} \#(6) \end{aligned}$$

$$E_{m}\simƝ(\theta_{K},\Sigma)$$

$$A_{m}\simƝ(\theta_{K},\tau_{m}^{2}\Lambda)$$

Where $Z_{m}$ is the Z score for the marker $m$ over a set of $K$ GWAS, $\mu_{m}$ quantifies the deviation to $H_{0}$ of marker $m$ and is common to all GWAS, $E_{m}$is the vector containing the residual error with $\Sigma$ the inter-environment correlation matrix,$A_{m}$ is the random effect accounting for the heterogeneity of the marker effect with $\tau_{m}^{2}$ and $\Lambda$ respectively the variance and the correlation matrix associated to the random marker effect. Testing the association of the marker $m$ corresponds to testing the null hypothesis of the mean marker effect $\mu_{m}$and the marker effect variance $\tau_{m}^{2}$ being equal to zero.

**Supplementary Method 8. Assessment of the proportion of genetic, G×L interaction and G×Y interaction variance explained jointly by all detected QTLs**

To assess the biological relevance of the high confident QTLs, we followed the approach previously described by [17,18]. A multi-environment mixed model with random effects for genotype (G), genotype by location interaction (G × L) and genotype by year interactions (G × Y) was first fitted to estimate the variance components of random effects by the restricted maximum likelihood (REML):

$$\begin{aligned} Y_{i,l,y}= \mu+{Env}_{l,y}+{PC}_{i}+{(PC\times Env)}_{i,l,y}+ \underline{G_{i}}+ \underline{{GL}_{i,l}}+\underline{{GY}_{i,y}}+ \underline{\varepsilon_{i,l,y}}\#(7) \end{aligned}$$

where $Y_{i,l,y}$ is the environment specific BLUPs of accession$i$, in location $l$ , and year$y$ *,* $\mu$ the overall mean, ${Env}_{l,y}$ the fixed effect of environment $l\times y$; ${PC}_{i}$ are coordinates of accession $i$ projected onto principal component analysis axes built with the kinship matrix K (only the axes with eigenvalues > 1 were retained); ${(PC\times Env)}_{i,l,y}$ are the fixed interaction effects between the genetic structure ${PC}_{i}$ and the environment $l\times y$; and $\varepsilon_{i,l,y}$the random independent and identically distributed residual term.

Then, a multi-locus multi-environment mixed model was fitted by adding the fixed effects of the QTLs and of the QTLs by environment interaction. When the number of QTLs considered is lower than 10, the following formula is used:

$$\begin{aligned} Y_{i,l,y}= \mu+{Env}_{l,y}+{PC}_{i}+{(PC\times Env)}_{i,l,y}+{QTL}_{i}+{(QTL\times Env)}_{i,l,y}+ \underline{G_{i}}+ \underline{{GL}_{i,l}}+\underline{{GY}_{i,y}}+ \underline{\varepsilon_{i,l,y}}\#(8.1) \end{aligned}$$

where${QTL}_{i}$, the fixed effect of QTLs, is the sum of the QTLs main effect.

Otherwise, if the number of QTLs considered is higher than 10, the information of the QTLs was aggregated as proposed by [19] and the following formula is used:

$$\begin{aligned} Y_{i,l,y}= \mu+{Env}_{l,y}+{PC}_{i}+\left( PC\times Env \right)_{i,l,y}+{PCQTL}_{i}+\left( PCQTL\times Env \right)_{i,l,y}+ \underline{G_{i}}+ \underline{{GL}_{i,l}}+\underline{{GY}_{i,y}}+ \underline{\varepsilon_{i,l,y}}\#(8.2) \end{aligned}$$

where${PCQTL}_{i}$, the fixed effect of QTLs, is estimated by the coordinates of the accession $i$ projected onto principal component analysis axes built with a kinship matrix computed with only the set of QTLs considered K (only the axes with eigenvalues > 1 were retained).

Finally, to assess the proportion of genetic, G×L interaction and G×Y interaction variance explained by the QTLs, we compared the estimated variance components obtained in (8.1 or 8.2) with those obtained from (7) from which we calculated the following indicator as proposed by [19]:

$$\begin{aligned} \gamma_{q, r}=\frac{\sigma_{r}^{2}- \sigma_{r}^{2*}}{\sigma_{r}^{2}} \#\left( 9 \right) \end{aligned}$$

$\gamma_{q, r}$ represent the proportion of variance captured by $q$, the set of QTLs, and $r$, the random effect being either G, G×L or G×Y, where $\sigma_{r}^{2}$ is the variance component of the random effect $r$ in (7) and$\sigma_{r}^{2*}$ is the variance component of the random effect $r$ in (8.1 or 8.2).

Supplementary Results

**Supplementary Result 1. Genetic structure and kinship analyses revealed an important structuration of the two core collections**

The genetic structure and relatedness of our peach core collection have been previously studied [20]. To summarize, a genetic structuration into three main groups has been identified with important levels of admixture (50% of the accessions admixed using a threshold of 0.8, and only six strictly assigned to one group). These structuration groups were consistent with the geographical origins of the accessions, with accessions in group 1 predominantly from Europe, accessions in group 2 largely from North America and accessions in group 3 from Asia. Additive kinship analyses have revealed the existence of three clusters of individuals with a higher degree of relatedness, mostly reflecting major breeding pools and similarities within the structural groups. The dominance kinship, reflected only two major groups.

For the apricot core collection, PCA, DAPC and SNMF analyses revealed a structuration in four main groups (Supplementary Fig 12). Groups 1 to 4 consisted in 34, 34, 49 and 32 accessions respectively with SNMF method (Supplementary Fig 12.A). Overall, 96% of the accessions were assigned to the same group using SNMF and DAPC methods, showing a high overlap between these two approaches. Only 9 accessions were strictly assigned to one group, the remaining being admixed between two to four groups with varying degrees. Using a threshold of 0.8 for significant admixture, we can consider that 75% of the apricot core collection is admixed. When looking at the geographical origin of accessions within each group, we found that group 1 is mainly represented by individuals from Central Asia, group 2 mainly by eastern Asia accessions, and groups 3 and 4 mainly by Mediterranean European accessions. Irano-Caucasian and Central European accessions are distributed in the group 1, 3 and 4. Relatedness between the different accessions is low on average, with additive kinship coefficient ranging from -0.12 to 0.45. Coefficients revealed a few clusters with stronger relatedness between accessions, such as between ‘Bergeron’, ‘Avikaline’, ‘Fantasme’, ‘Pêche de Nancy’ and ‘Boucheran’ which all belong to group 4. We observed a wider distribution of additive kinship values compared to the dominance ones with a standard deviation 1.7 times higher.


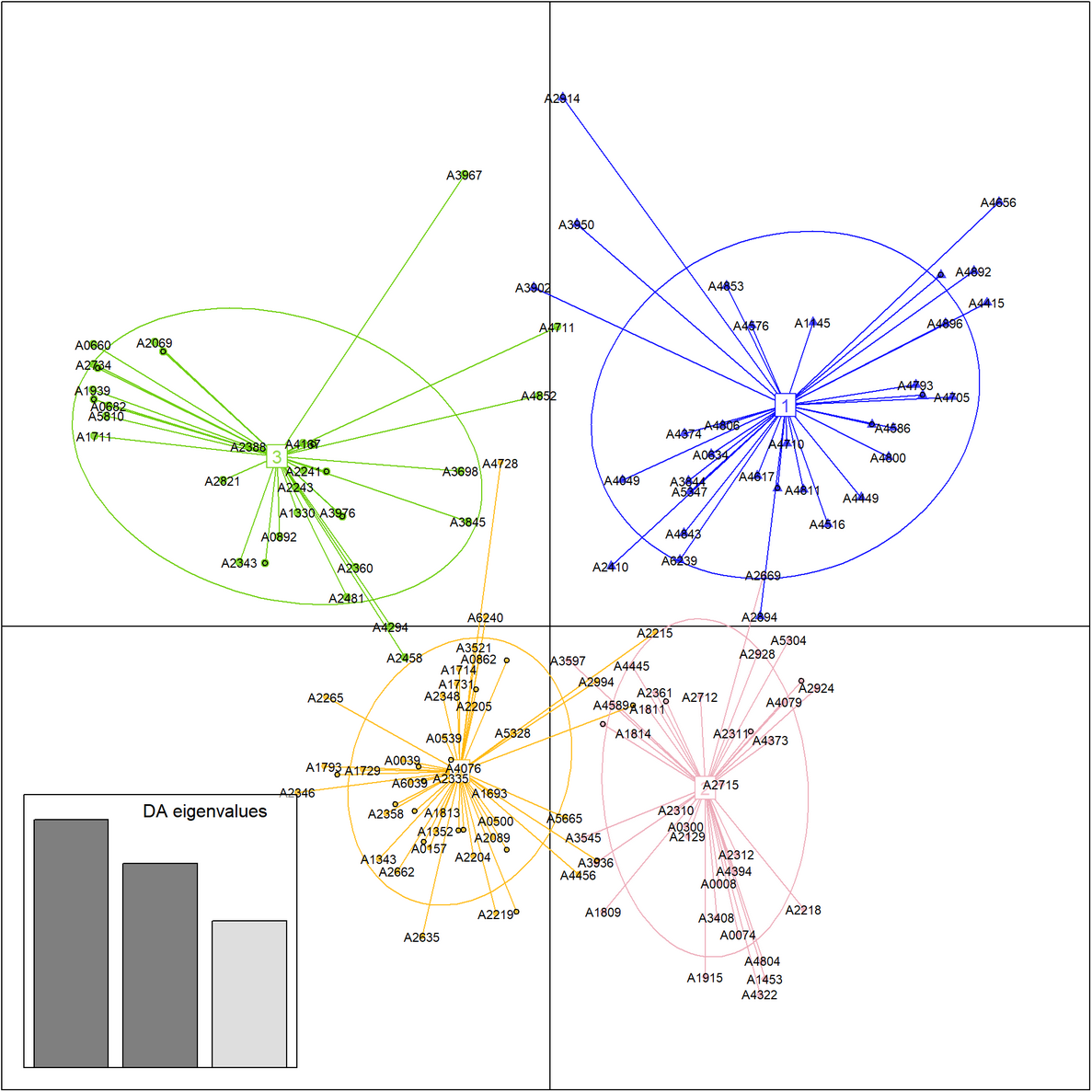

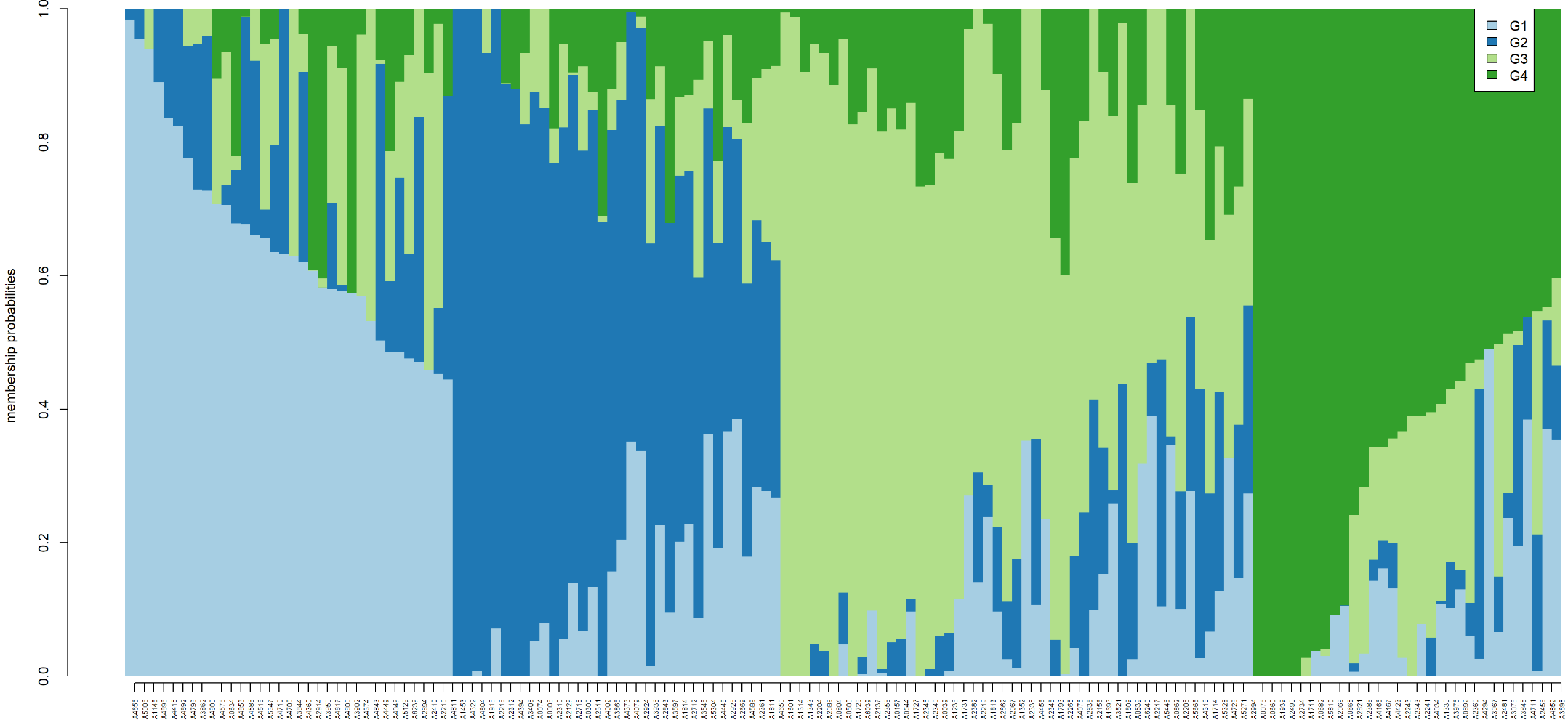


**A**als

**B**als

**Supplementary Fig 12.** Genetic structure of the apricot core collection represented by the 149 individuals genotyped with 41,145 SNPs markers after pruning. A) structure analysis with SNMF and B) discriminant analysis of principal components (DAPC).

References

1. Frichot E, François O. LEA: An R package for landscape and ecological association studies. *Methods in Ecology and Evolution* 2015;**6**:925–9.

2. Akaike H. A new look at the statistical model identification. *IEEE Transactions on Automatic Control* 1974;**19**:716–23.

3. Wissler C. The Spearman Correlation Formula. *Science* 1905;**22**:309–11.

4. Campbell CL, Madden LV. *Introduction to Plant Disease Epidemiology.*, 1990.

5. Box GEP, Cox DR. An Analysis of Transformations. *Journal of the Royal Statistical Society: Series B (Methodological)* 1964;**26**:211–43.

6. Holland JB, Nyquist WE, Cervantes-Martinez CT *et al.* Estimating and Interpreting heritability for plant breeding : an update. *Plant breeding reviews* 2003;**22**.

7. Schmidt P, Hartung J, Rath J *et al.* Estimating Broad-Sense Heritability with Unbalanced Data from Agricultural Cultivar Trials. *Crop Science* 2019;**59**:525–36.

8. Vitezica ZG, Legarra A, Toro MA *et al.* Orthogonal Estimates of Variances for Additive, Dominance, and Epistatic Effects in Populations. *Genetics* 2017;**206**:1297–307.

9. Roth M, Beugnot A, Mary-Huard T *et al.* Improving genomic predictions with inbreeding and nonadditive effects in two admixed maize hybrid populations in single and multienvironment contexts. Yu J (ed.). *Genetics* 2022;**220**:iyac018.

10. Laporte F, Charcosset A, Mary-Huard T. Efficient ReML inference in variance component mixed models using a Min-Max algorithm. *PLOS Computational Biology* 2022;**18**:e1009659.

11. Dray S, Dufour A-B. The ade4 Package: Implementing the Duality Diagram for Ecologists. *Journal of Statistical Software* 2007;**22**:1–20.

12. Jombart T, Devillard S, Balloux F. Discriminant analysis of principal components: a new method for the analysis of genetically structured populations. *BMC Genetics* 2010;**11**:94.

13. Frichot E, Mathieu F, Trouillon T *et al.* Fast and Efficient Estimation of Individual Ancestry Coefficients. *Genetics* 2014;**196**:973–83.

14. Evanno G, Regnaut S, Goudet J. Detecting the number of clusters of individuals using the software structure: a simulation study. *Molecular Ecology* 2005;**14**:2611–20.

15. Korte A, Vilhjálmsson BJ, Segura V *et al.* A mixed-model approach for genome-wide association studies of correlated traits in structured populations. *Nat Genet* 2012;**44**:1066–71.

16. Munoz F. breedR: Statistical methods for forest genetic resources analysts. 2015.

17. van Eeuwijk FA, Bink MC, Chenu K *et al.* Detection and use of QTL for complex traits in multiple environments. *Current Opinion in Plant Biology* 2010;**13**:193–205.

18. Millet EJ, Welcker C, Kruijer W *et al.* Genome-Wide Analysis of Yield in Europe: Allelic Effects Vary with Drought and Heat Scenarios. *Plant Physiology* 2016;**172**:749–64.

19. Djabali Y, Rincent R, Martin M-L *et al.* Plasticity QTLs specifically contribute to the genotype × water availability interaction in maize. *Theor Appl Genet* 2023;**136**:228.

20. Roth M, Serrie M, Dlalah N *et al.* Genetic diversity in a new peach core-collection designed for resilience breeding. *Acta Hortic* 2022:141–8.
